# Structure-based design generated novel hydroxamic acid based preferential HDAC6 lead inhibitor with on-target cytotoxic activity against primary choroid plexus carcinoma

**DOI:** 10.1080/14756366.2019.1613987

**Published:** 2019-05-09

**Authors:** Shaymaa E. Kassab, Samar Mowafy, Aya M. Alserw, Joustin A. Seliem, Shahenda M. El-Naggar, Nesreen N. Omar, Mohamed M. Awad

**Affiliations:** a Pharmaceutical Chemistry Department, Faulty of Pharmacy, Damanhour University, Damanhour, Egypt;; b Pharmaceutical Chemistry Department, Faculty of Pharmacy, Misr International University, Cairo, Egypt;; c Basic Research Unit, Department of Research, Children's Cancer Hospital in Egypt, Cairo, Egypt;; d Biochemistry Department, Faculty of Pharmacy, Modern University for Technology and Information, Cairo, Egypt;; e Department of Pharmacology and Toxicology, Faculty of Pharmacy, Helwan University, Cairo, Egypt;; f Canadian Academy of Research and Development (CARD), Mississauga, ON, Canada

**Keywords:** Preferential HDAC6 inhibitor, acetylated-α-tubulin, on-target activity, benzimidazole, acute promyeloblastic leukemia, choroid plexus carcinoma, cytotoxicity

## Abstract

Histone deacetylase 6 (HDAC6) is an attractive target for cancer therapeutic intervention. Selective HDAC6 inhibitors is important to minimise the side effects of pan inhibition. Thus, new class of hydroxamic acid-based derivatives were designed on structural basis to perform preferential activity against HDAC6 targeting solid tumours. Interestingly, 1-benzylbenzimidazole-2-thio-*N*-hydroxybutanamide **10a** showed impressive preference with submicromolar potency against HDAC6 (IC_50_ = 510 nM). **10a** showed cytotoxic activity with interesting profile against CCHE-45 at (IC_50_ = 112.76 µM) when compared to standard inhibitor Tubacin (IC_50_ = 20 µM). Western blot analysis of acetylated-α-tubulin verified the HDAC6 inhibiting activity of **10a**. Moreover, the insignificant difference in acetylated-α-tubulin induced by **10a** and Tubacin implied the on-target cytotoxic activity of **10a**. Docking of **10a** in the binding site of HDAC6 attributed the activity of **10a** to π-π stacking with the amino acids of the hydrophobic channel of HDAC6 and capture of zinc metal in bidentate fashion. The therapeutic usefulness besides the on-target activity may define **10a** as an interesting safe-lead inhibitor for future development.

## Graphical Abstract

**Figure:**
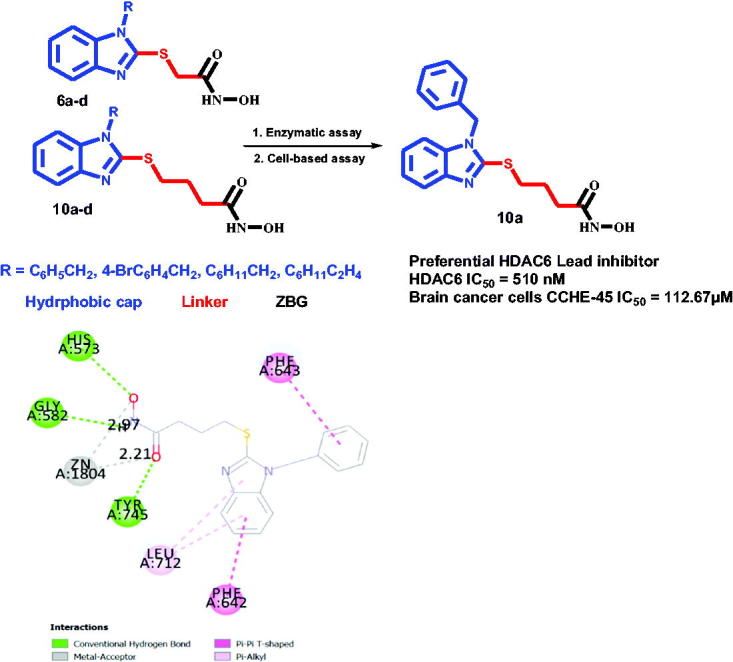

## 1. Introduction

Mechanisms and signalling pathways that lead to transformation of normal cell into cancer cell occupy a remarkable space in the experimental oncology researches. This type of biological studies succeeded to identify some important genes^1^^,^^2^, proteins^3^, transcriptional and epigenetic factors^4–6^ that contribute to the hallmarks of cancer^7^. Epigenetic modifications are associated with changes in gene transcription, and alteration in chromatin structure^8^. The main epigenetic modifications include histone methylation and acetylation^8^ the reversible addition and removal of acetyl group is governed by the controlled expression and activity of histone acetyltransferases (HATs) as well as histone deacetylases (HDACs)^9^^,^^10^. Regularly, chromatin is switched between two states: the loose state and condensed state. The loose form of the chromatin (euchromatin) in which the histone protein is acetylated in lysine residues by HATs, exposes genes for transcription^11^. On contrary, HDACs are associated with chromatin condensed form^12^, this heterochromatin structure intervenes with gene expression.

There are four classes and two families of human HDACs involving 18 mammalian isoforms with different physiological functions and distinct cellular compartments; some are either solely present in the nucleus or the cytosol, while other isoforms are shuttling between the nucleus and the cytosol^13–15^. Eleven isoforms of deacetylases (HDAC 1–11) which constitute the classical family, share similar structure, and require Zn^2+^ for their activity onset. While the sirtuin family contains 7 isoforms (SIRT 1–7), which are structurally different from the classical family, and they are NAD-dependent^16^^,^^17^. HDACs are deregulated in different cancer types,^8^ however, the intricate physiological role of HDAC and its involvement in normal cell proliferation^18^, mitosis, ^19^ development, cardiac morphogenesis^20^, signal transduction pathways^21^^,^^22^, and apoptosis^19^^,^^23–25^ made pan inhibition of HDACs leads to exhibition of toxicity^26–30^. Researchers worked extensively on providing structural determinants for the generation of selective or even preferential inhibitors against one isoform^12^^,^^31–33^ or group of isoforms^13^^,^^34^^,^^35^. Selective HDAC inhibition reduces the toxicity that results from complete shutdown of normal physiological functions of HDACs due to pan inhibition. It was reported that the zinc-dependent HDAC isoforms require an inhibitor with the following characterisations: hydrophobic cap, metal deter and a linker to link between the metal deter and the cap^36^. Only HDAC6 and HDAC8 that can accommodate large hydrophobic cap due to the presence of a second tube-like pocket with a different shape and close to the active hydrophobic pocket^37^^,^^38^ when compared to the other isoforms, but the difference between HDAC6 and HDAC8 is the protein surface flexibility that was identified as a factor of selectivity^36^. The other isoforms are closely similar and the elaboration of selective inhibitors against them is yet, very limited and not feasible to achieve^36^. Thus, many selective inhibitors that were discovered where almost against HDAC6 and/or HDAC8 due to the distinct protein structural differences between these two specific isoforms and other isoforms.

**Figure 1.**
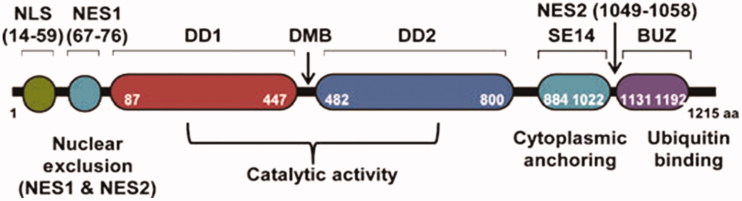
Schematic representation and functional domains of human HDAC6. HDAC6 is the only HDAC with two tandem deacetylase domains (DD1 and DD2) including catalytic activity. A nuclear export signal (NES) prevents the accumulation of the protein in the nucleus and the Ser-Glu-containing tetrapeptide (SE14) region ensures stable anchorage of the enzyme in the cytoplasm. The nuclear localisation signal (NLS) translocates HDAC6 into nucleus. The linker (dynein motor binding, DMB) between both CATs can bind to dynein and the high affinity ubiquitin-binding zinc finger domain (BUZ). aa, amino acid.

HDAC6 is found to be overexpressed in several cancer cell types^39–44^, it is also implicated in the onset or the progression of many neurodegenerative diseases^45–47^ and autoimmune disorders^48–50^. HDAC6 protein is the only isoform with two active deacetylase domains that are identical and function independently^51^; the linker between the two domains is the dynein motor binding (DMB) domain, and cytoplasmic retention signal (SE14) motif that enables the enzyme to reside in the cytoplasm to perform its functions regularly (Figure 1)^52^. Zinc finger ubiquitin binding domain (BUZ) is located at the *C*-terminal and is absent in the other HDAC isoforms^14^. Due to that unique structure, and cytoplasmic localisation, HDAC6 is able to deacetylate non-histone proteins; such as α-tubulin, heat shock protein 90 (Hsp90), and cortactin^53^. The post-translational modification of these non-histone proteins contribute to cancer cell proliferation, migration, protein homoeostasis, regulation of expression of critical immune modulators, the stability and activity of transcriptional factors such as hypoxia inducible factors (HIFs), the activity of estrogenic and androgenic receptors, and platelet aggregation in coagulation process^14^^,^^36^^,^^44^^,^^53^^,^^54^.

**Figure 2.**
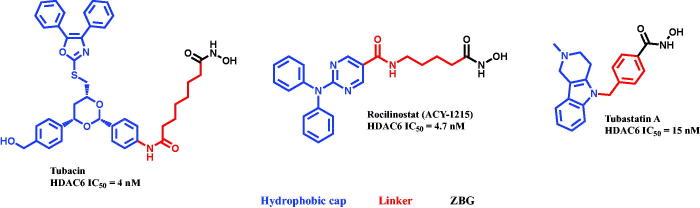
Representative examples of most popular selective HDAC6 inhibitors with the corresponding potencies.

Upon surveying the literature on the chemical structures of the reported selective HDAC6 inhibitors, we have found that all are with large rigid or non-rigid cap; a common structural feature as depicted in the representative examples; Tubacin^55^, Rocillinostat^39^ and Tubastatin A^56^ (Figure 2). The linker properties regarding the length and saturation/unsaturation varied from structure to another, and ZBG group was conserved with all the reported structures^57^. Tubacin that is the first reported as potent selective HDAC6 inhibitor (HDAC6 IC_50_  = 4 nM)^55^^,^^56^; however, it has never been able to be tested *in vivo* due to its complex structure and high lipophilicity^44^. Rocillinostat; although it is the considered the first selective inhibitor of HDAC6, which is orally available and has moved to phase I/II of clinical trials for treating multiple myeloma as well as lymphoma^58–60^, its potency in solid tumours is very low as a single agent^61^ and has not been extensively investigated. Tubastatin A was reported as selective HDAC6 inhibitor with neuroprotective activity^56^ and no study proved its potential antiproliferative activity against any type of cancer, but it increased the sensitivity of non-small cell lung cancer (NSCLC) to cisplatin^62^. Furthermore, Tubastatin A is of rigid tertahydro-γ-carboline hydrophobic cap that is not accessible to introduce different variables to develop but ring extension^31^. This gap encouraged scientists to initiate serious attempts to identify new HDAC6 selective inhibitors to combat specific types of cancer. Accordingly, we aimed at generating a lead compound that initially performs preferential HDAC6 inhibition, with new chemical entity, and is feasible to be synthesised and developed. Enzymatic assay against all human HDAC isoforms are going to be conducted to determine the preferential inhibition activity of the synthesised compounds, which will be coupled with the cytotoxic activity against specific type of children brain cancer; primary choroid plexus carcinoma (CCHE-45) and cell-based assays for detection of acetylated α-tubulin.

Thus, it is important to emphasise the critical approaches toward suggesting a structural design for elaboration of new lead with preferential HDAC6 inhibiting activity: *i) large hydrophobic cap for possible accommodation in the HDAC6 hydrophobic channel which reduces the chance of perfect fitting with the other HDAC isoforms, ii) different linker lengths were used because no definite report generally defines the recommended length of linker, iii) zinc binding group (ZBG) is included as a conservative fragment*^63^^,^^64^*that is essential to capture the zinc metal of the enzyme to stop its catalysis and enhance the stable residence of inhibitor into the catalytic domain.*

**Figure 3.**
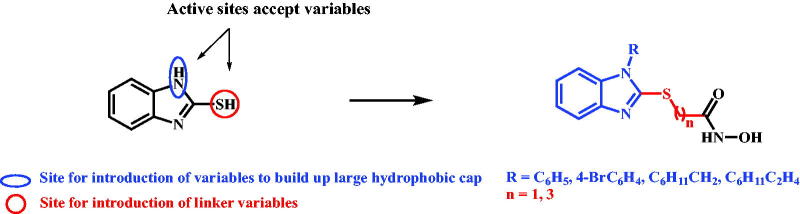
Rational design of preferential HDAC6 lead inhibitor.

Consequently, it was suggested to work on binuclear aromatic heterocycle with flexible chemical entity to accommodate different variables for further modifications that might be useful for future development of the generated lead inhibitor to improve both the potency and selectivity. 2-Mercaptobenzimidazole was chosen as the scaffold of the target inhibitor based on the accessibility of two structural active sites for introduction of different variables which are N^1^-benzimidazole and 2-mercaptobenzimidazole. Both sites, are acidic with different pKa values, would play a role in optimisation of the reaction conditions to introduce the different variables at the target site and avoid multiple alkylation or even getting undesirable regioisomers (Figure 3).

## 2. Materials and methods

### 2.1. Chemistry

Melting points were determined on digital Gallen-Kamp MFB-595 instrument using open capillary tubes and are uncorrected. IR spectra were recorded as potassium bromide discs on Schimadzu FT-IR 440 spectrometer. ^1^H NMR spectra were recorded on Bruker spectrophotometer at 400 MHz in DMSO-d_6_; values (δ) are given in parts per million (ppm) downfield from tetramethylsilane (TMS) as internal reference. ^13 ^C NMR spectra were recorded using the same spectrophotometer that used for recording ^1^H NMR at 101 MHz in DMSO-d_6_. Mass spectra were recorded on ACQUITY UPC2 System mass spectrometer for ES detection and Schimadzu Triple Quadrupole GC-MS mass spectrometer for EI detection. The elemental analyses were performed at the Microanalytical Center, Cairo University, Cairo, Egypt. Reactions were followed up by thin layer chromatography (TLC) using Merck Silica gel/TLC cards with fluorescent indicator UV254 using Hexane:Ethyl acetate (EtOAc) 4:1 and DCM:MeOH 9.5:0.5 as the eluting systems and the spots were visualised using Spectroline E series dual wavelength UV lamp at λ = 254 nm.

#### 2.1.1. Synthesis of ethyl (1H-benzo[d]imidazol-2-yl)thioacetate (3)

A mixture of 2-mercaptobenzimidazole **1** (0.75 g, 5 mmol) and ethyl chloroacetate **2** (0.75 ml, 7.5 mmol) was added to a stirred solution of absolute ethanol at 60°^ ^C, then anhydrous K_2_CO_3_ (1.035 g, 7.5 mmol) was added. The reaction mixture was left stirring overnight. The inorganic salts were filtered off, the solvent was evaporated under reduced pressure and the white organic residue was washed with water several times to furnish white fibrous crystals that was pure enough to be submitted to the next step of the reactions.

Yield: 34%, m.p.: 60 °C, white microcrystals; **^1^H NMR (DMSO-d_6_, 400 MHz)**: δ 12.60 (*s*, 1H, NH), 7.48 (d, *J* = 5.1 Hz, 1H, 8-H), 7.38 (*s*, 1H, 5-H), 7.11 (dd, *J* = 5.7, 2.2 Hz, 2H, 6,7-H), 4.20 (*s*, 2H, S-CH_2_), 4.12 (*q*, *J* = 7.1 Hz, 2H, COOCH_2_CH_3_), 1.17 (*t*, *J* = 7.1 Hz, 3H, COOCH_2_CH_3_).

#### 2.1.2. General synthesis of ethyl 2-((1-(benzyl/cycloalkyl-1H-benzo[d]imidazol-2-yl)thio)acetates (5a-d)

Anhydrous K_2_CO_3_ (0.21 g, 1.5 mmol) was added to a stirred mixture of ethyl (benzimidadol-2-yl)thioacetate **3** (0.236 g, 1 mmol) and benzyl chloride 4a (0.12 ml, 1 mmol) or 4-bromobenzyl bromide **4b** (0.25 g, 1 mmol) or cyclohexylmethyl bromide **4c** (0.14 ml, 1 mmol) or cyclohexylethyl bromide **4d** (0.16 ml, 1 mmol) in DMF and the temperature was raised to 100°^ ^C. The reaction mixture was judged complete after 1 h upon checking with TLC using Hexane:EtOAc 4:1 as eluting system. The reaction mixture was poured onto water and the organic product was extracted by ethyl acetate, the organic extract was washed with water several times then dried over anhydrous sodium sulphate. The solvent was evaporated under reduced pressure to give the product as dark oil residue. All trials to recrystallise the product from Methanol or ethanol furnished only Ethyl 2-((1–(4-bromobenzyl)-1*H*-benzo[*d*]imidazol-2-yl)thio)acetate **(5b)** from methanol as golden yellow microcrystals but all the other derivatives **5a, 5c,** and **5d** were obtained as oil products in yields 45%, 54% and 65% respectively with enough purity to be submitted to the next step of reactions.

#### 2.1.3. Ethyl 2-((1–(4-bromobenzyl)-1H-benzo[d]imidazol-2-yl)thio)acetate (5b)

Yield: 40%, m.p.: 107 °C, golden yellow needles; IR (KBr) ʋ_max_/cm^−1^:1741 (CO). **^1^H NMR (DMSO-d_6_, 400 MHz)**: δ 7.56–7.52 (*m*, 3H, Ar-H), 7.50–7.46 (*m*, 1H, Ar-H), 7.20–7.14 (*m*, 4H, Ar-H), 5.41 (*s*, 2H, 4-BrC_6_H_4_CH_2_), 4.26 (*s*, 2H, S-CH_2_), 4.11 (*q*, *J* = 7.1 Hz, 2H, COOCH_2_CH_3_), 1.16 (*t*, *J* = 7.1 Hz, 3H, COOCH_2_CH_3_). **^13 ^C NMR (DMSO-d_6_, 101 MHz)**: δ 168.35 (CO), 150.63 (S-C), 142.86, 136.22, 135.73, 131.68, 129.29, 122.01, 121.88, 120.94, 117.85, and 109.85 (Ar-C), 61.27 (COOCH_2_CH_3_), 46.11(4-BrC_6_H_4_CH_2)_, 33.83(S-CH_2_), 14.01 (COOCH_2_CH_3_). **MS LRMS (EI)**: (calc) 404.02 (found) 404.08 (M)+. Anal. Calcd for C_18_H_17_BrN_2_O_2_S: C, 53.34; H, 4.23; N, 6.91. Found: C, 53.56; H, 4.11; N, 7.19.

#### 2.1.4. General synthesis of 2-((1-benzyl/cycloalkyl-1H-benzo[d]imidazol-2-yl)thio)-N-hydroxyacetamides (6a-d)

Hydroxylamine hydrochloride (10 equivalent) was neutralised in 1 M NaOMe solution in methanol (14 equivalent) via stirring for 10 min and filtered off to discard sodium chloride. Hydroxylamine solution was added to a stirred solution of the respective ester **(5a-d)** (1 equivalent) in methanol (5 ml) at room temperature. The reaction mixture was left stirring until judged complete upon observing disappearance of the spot of ester starting material on TLC after 10 min using DCM:MeOH 9.5:0.5 as eluting system. The solvent was evaporated under reduced pressure and the crude residue was quenched with distilled water (15–20 ml). All the organic dirt of the aqueous reaction mixture was scavenged while vigorous shaking with EtOAc and the aqueous extract of the product was separated free from the organic dirt remained from the reaction. The aqueous extract of the product was acidified with drop wise addition of 10% HCl until observing precipitation at pH = 5.0. The resulting suspension was left in the fridge overnight for the product to complete its precipitation. The precipitate was filtered off, washed several times with distilled water and dried under vacuum to give crystalline product.

#### 2.1.5. 2-((1-benzyl-1H-benzo[d]imidazol-2-yl)thio)-N-hydroxyacetamide (6a)

Yield: 22.0%, m.p.: 97 °C, golden yellow microcrystals; IR (KBr) ʋ_max_/cm^−1^: 3427 (broad band NH/OH), 1717 (CO). **^1^H NMR (DMSO-d_6_, 400 MHz)**: δ 7.57 – 7.51 (*m*, 1H, Ar-H)), 7.51–7.44 (*m*, 1H, Ar-H)), 7.36–7.22 (*m*, 5H, Ar-H), 7.19–7.12 (*m*, 2H, Ar-H), 5.41 (*s*, 2H, C_6_H_5_CH_2_), 4.20 (*s*, 2H, S-CH_2_), 3.60 (broad s, 2H). **^13 ^C NMR (DMSO-d_6_, 101 MHz)**:δ 169.68 (CO), 151.16 (S-C), 142.91, 136.32, 128.82, 127.85, 127.20, 121.92, 121.82, 117.77, 109.92 (Ar-C), 46.82 (C_6_H_5_CH_2__)_, 34.37 (S-CH_2_). **MS LCMS (ES)**: (calc) 313.09 (found) 314.1561 (MH)^+.^ Anal. Calcd for C_16_H_15_N_3_O_2_S: C, 61.32; H, 4.82; N, 13.41. Found: C, 61.49; H, 5.02; N, 13.25.

#### 2.1.6. 2-((1–(4-bromobenzyl)-1H-benzo[d]imidazol-2-yl)thio)-N-hydroxyacetamide (6b)

Yield: 19.0%, m.p.: 167 °C, golden yellow microcrystals; IR (KBr) ʋ_max_/cm^−1^: 3405 (broad band NH/OH), 1713 (CO). **^1^H NMR (DMSO-d_6_, 400 MHz)**: δ 7.52 (d, *J* = 8.1 Hz, 3H, Ar-H), 7.45 (d, *J* = 4.8 Hz, 1H, Ar-H), 7.16 (dd, *J* = 11.6, 6.1 Hz, 4H, Ar-H), 5.39 (*s*, 2H, 4-BrC_6_H_4_CH_2_), 4.46 (broad s, 2H), 4.14 (*s*, 2H, S-CH_2_). **^13 ^C NMR (DMSO-d_6_, 101 MHz)**: δ 169.84 (CO), 151.73 (S-C), 142.97, 136.09, 135.81, 131.65, 129.31, 121.79, 121.72, 120.86, 117.70, and 109.68 (Ar-C), 46.08 (4-BrC_6_H_4_CH_2_), 36.04 (S-CH_2_). **MS LCMS (ES)**: (calc) 391.00 (found) 392.0124(MH)^+.^ Anal. Calcd for C_16_H_14_BrN_3_O_2_S: C, 48.99; H, 3.60; N, 10.71. Found: C,48.71; H, 3.81; N, 10.94.

#### 2.1.7. 2-((1-(Cyclohexylmethyl)-1H-benzo[d]imidazol-2-yl)thio)-N-hydroxyacetamide (6c)

Yield: 10.0%, m.p.: 160 °C, golden yellow needle crystals; IR (KBr) ʋ_max_/cm^−1^: 3429 (broad band NH/OH), 1712 (CO). **^1^H NMR (DMSO-d_6_, 400 MHz)**: δ 7.50 (dd, *J* = 8.4, 6.8 Hz, 2H, 6,7-H), 7.21 – 7.09 (*m*, 2H, 6,7-H), 4.18 (*s*, 2H, S-CH_2_), 3.98 (d, *J* = 7.3 Hz, 2H, C_6_H_11_CH_2_), 3.45 (broad *s*, 2H), 1.82–1.86 (*m*, 1H, C_6_H_11_-H), 1.66–1.51 (*m*, 5H, C_6_H_11_-H), 1.13–1.04 (*m*, 5H, C_6_H_11-_H). **^13 ^C NMR (DMSO-d_6_, 101 MHz)**: δ 169.68 (CO), 151.06 (S-CH_2_), 142.64, 136.72, 121.64, 121.50, 117.60, and 110.02 (Ar-C), 49.60 (C_6_H_11_CH_2_), 37.81(S-CH_2_), 34.29 (C_6_H_11_-C-1), 30.23(C_6_H_11_-C-2,6), 25.80 (C_6_H_11_-C-3,5), 25.22 (C_6_H_11_-C-4). **MS LCMS (ES)**: (calc) 319.14 (found) 320.1853 (MH)^+.^ Anal. Calcd for C_16_H_21_N_3_O_2_S: C, 60.16; H, 6.63; N, 13.16. Found: C, 60.37; H, 6.94; N, 13.38.

#### 2.1.8. 2-((1–(2-cyclohexylethyl)-1H-benzo[d]imidazol-2-yl)thio)-N-hydroxyacetamide (6d)

Yield: 9.0%, m.p.: 157 °C, light brown fibrous crystals; IR (KBr) ʋ_max_/cm^−1^: 3430 (broad band NH/OH), 1712 (CO). **^1^H NMR (DMSO-d_6_, 400 MHz)**: δ 7.50 (dd, *J* = 18.6, 6.7 Hz, 2H, 5,8-H), 7.18 (d, *J* = 6.3 Hz, 2H, 6,7-H), 4.18 (*m*, 4H, S-CH_2_, C_6_H_11_CH_2_CH_2_), 3.41 (broad *s*, 2H), 1.78–1.61 (*m*, 7H, C_6_H_11_-H), 1.30–1.18 (*m*, 4H, C_6_H_11_-H), 0.96 (*q*, *J* = 10.9 Hz, 2H, C_6_H_11_-H). **^13 ^C NMR (DMSO-d_6_, 101 MHz)**: δ 169.65 (CO), 150.56 (S-C), 142.83, 136.05, 121.68, 121.53, 117.68, and 109.52 (Ar-C), 41.57 (C_6_H_11_CH_2_CH_2_), 36.32 (S-CH_2_), 34.55 (C_6_H_11_CH_2_CH_2_), 34.10 (C_6_H_11_-C-1), 32.62 (C_6_H_11_-C-2,6), 26.01 (C_6_H_11_-C-3,5), 25.65 (C_6_H_11_-C-4). **MS LCMS (ES)**: (calc) 333.15 (found) 334.2125 (MH)^+.^ Anal. Calcd for C_17_H_23_N_3_O_2_S: C, 61.23; H, 6.95; N, 12.60. Found: C, 61.47; H, 7.20; N, 12.91.

#### 2.1.9. Methyl 4-((1H-benzo[d]imidazol-2-yl)thio)butanoate (8)

Potassium hydroxide (0.112 g, 2 mmol) was dissolved in DMF (5 ml) upon stirring at 90°^ ^C, the resulting solution left to cool to room temperature, then benzimidazole-2-thiol **(1)** (0.15 g, 1 mmol) and methyl 4-chlorobutyrate **(7)** (0.145 ml, 1.20 mmol) were added to the solution and left stirring overnight at room temperature. The reaction was found very complete upon checking with TLC using Hexane:EtOAc3:1. The resulting reaction mixture was poured onto ice-cold water to give milky solution of oil product. The product was extracted with EtOAc and the organic extract was washed several times with water and dried over anhydrous sodium sulphate. The solvent was evaporated under reduced pressure to give yellowish oil product that upon standing at room temperature for 10 h furnished off-white needle crystals of the product.

Yield: 76.0%, m.p.: 122 °C, off-white needle crystals; IR (KBr) ʋ_max_/cm^−1^: 3435 (NH), 1752 (CO). **^1^H NMR (DMSO-d_6_, 400 MHz)**: δ 12.54 (*s*, 1H, NH), 7.43 (*s*, 2H, 5,8-H), 7.11 (dd, *J* = 5.9, 3.2 Hz, 2H, 6,7-H), 3.58 (*s*, 3H, OCH_3_), 3.29 (*t*, *J* = 7.1 Hz, 2H, S-CH_2_), 2.47 (*t*, *J* = 7.5 Hz, 2H, 2-CH_2_), 2.01–1.97 (*p*, *J* = 7.2, 2H, 3-CH_2_).**^13^C NMR (DMSO-d_6_, 101 MHz)**: δ 172.81 (CO), 149.83 (S-C), 121.35 (Ar-C), 51.37 (COOCH_3_), 32.00 (S-CH_2_), 30.43 (2-CH_2_), 24.79 (3-CH_2_). **MS LRMS (EI)**: (calc) 250.08 (found) 250.12 (M)^+.^ Anal. Calcd for C_12_H_14_N_2_O_2_S: C, 57.58; H, 5.64; N, 11.19. Found: C, 57.82; H, 5.31; N, 11.45.

#### 2.1.10. General synthesis of methyl 4-((1-benzyl/cycloalkyl-1H-benzo[d]imidazol-2-yl)thio)butanoates (9a-d)

Anhydrous K_2_CO_3_ (0.21 g, 1.5 mmol) was added to a stirred mixture of Methyl 4-((1*H*-benzo[*d*]imidazol-2-yl)thio)butanoate **8** (0.25 g, 1 mmol) and benzyl chloride **4a** (0.12 ml, 1 mmol) or 4-bromobenzyl bromide **4b** (0.25 g, 1 mmol) or cyclohexylmethyl bromide **4c** (0.14 ml, 1 mmol) or cyclohexylethyl bromide **4d** (0.16 ml, 1 mmol) in DMF and the temperature was raised to100 ^o^C. The reaction mixture was judged complete after 2 h upon checking with TLC using Hexane:EtOAc 4:1 as eluting system. The reaction mixture was poured onto water and the organic product was extracted by ethyl acetate, the organic extract was washed with water several times and dried over anhydrous sodium sulfate. The solvent was evaporated under reduced pressure to give the product as yellowish oil residue. The intermediate ester derivatives **(9a-d)** were obtained in yields 85%, 69%, 90%, and 97% respectively with enough purity to be submitted to the next step of reactions without further purification.

#### 2.1.11. General synthesis of 4-((1-benzyl/cycloalkyl-1H-benzo[d]imidazol-2-yl)thio)-N-hydroxybutanamides (10a-d)

Hydroxylamine hydrochloride (10 equivalent) was neutralised in 1 M NaOMe solution in methanol (14 equivalent) via stirring for 10 min and filtered off to discard sodium chloride. Hydroxylamine solution was added to a stirred solution of the respective ester **(5a-d)** (1 equivalent) in methanol (5 ml) at room temperature. The reaction mixture was left stirring until judged complete upon observing disappearance of the spot of ester starting material on TLC after 2 h using DCM:MeOH 9.5:0.5 as eluting system. The solvent was evaporated under reduced pressure and the crude residue was quenched with distilled water (15–20 ml). All the organic dirt of the aqueous reaction mixture was scavenged while vigorous shaking with EtOAc and the aqueous extract of the product was separated free from the organic dirt that remained from the reaction. The aqueous extract of the product was acidified with drop wise addition of 10% HCl until observing precipitation at pH  = 5.0. The resulting suspension was left in the fridge overnight for the product to complete its precipitation. The resulting precipitate was filtered off, washed several times with distilled water and dried under vacuum to give powder product. The resulting products were recrystallised from Hexane:EtOAc 1:1 to afford crystalline products.

#### 2.1.12. 4-((1-benzyl-1H-benzo[d]imidazol-2-yl)thio)-N-hydroxybutanamide (10a)

Yield: 25.0%, m.p.: 145 °C, off-white microcrystals; IR (KBr) ʋ_max_/cm^−1^: 3426 (broad band NH/OH), 1710 (CO). **^1^H NMR (DMSO-d_6_, 400 MHz)**: δ 7.61–7.53 (*m*, 1H, Ar-H), 7.50–7.43 (*m*, 1H, Ar-H), 7.36–7.22 (*m*, 3H, Ar-H), 7.22–7.09 (*m*, 4H, Ar-H), 5.39 (*s*, 2H, C_6_H_5_CH_2_), 3.33–3.37 (*m*, 2H, S-CH_2_), 2.37 (*t*, *J* = 7.3 Hz, 2H, 2-CH_2_), 1.95 (*m*, 2H, 3-CH_2_). **^13^C NMR (DMSO-d_6_, 101 MHz)**: δ 173.98 (CO), 151.43 (S-C), 143.09, 136.43, 136.43, 136.20, 136.20, 128.80, 128.80, 127.75, 127.75, 127.01, 127.01, 121.82, 121.73, 117.76, and 109.85 (Ar-C), 46.69 (C_6_H_5_-CH_2_), 32.42 (S-CH_2_), 31.25 (2-CH_2_), 24.56 (3-CH_2_). **MS LCMS (ES)**: (calc) 341.12 (found) 342.2353 (MH)^+.^ Anal. Calcd for C_18_H_19_N_3_O_2_S: C, 63.32; H, 5.61; N, 12.31. Found: C, 63.50; H, 5.46; N, 12.57.

#### 2.1.13. 4-((1–(4-bromobenzyl)-1H-benzo[d]imidazol-2-yl)thio)-N-hydroxybutanamide (10b)

Yield: 30.0%, m.p.: 153 °C, off-white macrocrystals; IR (KBr) ʋ_max_/cm^−1^: 3430 (broad band NH/OH), 1710 (CO). **^1^H NMR (DMSO-d_6_, 400 MHz)**: δ 12.19 (*s*, 1H), 7.62–7.56 (*m*, 1H, Ar-H), 7.53 (d, *J* = 8.3 Hz, 2H), 7.49–7.43 (*m*, 1H), 7.15 (dt, *J* = 18.5, 7.4 Hz, 4H), 5.38 (*s*, 2H, 4-BrC_6_H_4_-CH_2_), 3.33–3.37 (*m*, 2H, S-CH_2_), 2.37 (*t*, *J* = 7.3 Hz, 2H, 2-CH_2_), 2.00–1.90 (*m*, 2H, 3-CH_2_). **^13 ^C NMR (DMSO-d_6_, 101 MHz)**: δ 173.90 (CO), 151.33 (S-C), 143.04, 136.08, 135.86, 131.67, 129.16, 121.84, 121.76, 120.82, 117.77, and 109.74 (Ar-C), 45.99 (4-BrC_6_H_4_-CH_2_), 32.36 (S-CH_2_), 31.22 (2-CH_2_), 24.49 (3-CH_2_). **MS LCMS (ES)**: (calc) 419.03 (found) 420.1528 (MH)^+.^ Anal. Calcd for C_18_H_18_BrN_3_O_2_S: C, 51.44; H, 4.32; N, 10.00. Found: C, 51.69; H, 4.08; N, 9.77.

#### 2.1.14. 4-((1-(Cyclohexylmethyl)-1H-benzo[d]imidazol-2-yl)thio)-N-hydroxybutanamide (10c)

Yield: 35.0%, m.p.: 149 °C, off-white microcrystals; IR (KBr) ʋ_max_/cm^−1^: 3426 (broad band NH/OH), 1716 (CO). **^1^H NMR (DMSO-d_6_, 400 MHz)**: δ 12.18 (*s*, 1H), 7.54 (d, *J* = 6.6 Hz, 1H, 8-H), 7.45 (d, *J* = 6.3 Hz, 1H, 5-H), 7.22–7.03 (*m*, 2H, 6,7-H), 3.93 (d, *J* = 7.0 Hz, 2H, C_6_H_11_-CH_2_), 3.35 (*t*, *J* = 6.8 Hz, 2H, S-CH_2_), 2.39 (*t*, *J* = 7.1 Hz, 2H, 2-CH_2_), 2.05–1.87 (*m*, 2H, 3-CH_2_), 1.81 (*m*, 1H, C_6_H_11_-H), 1.62–1.48 (*m*, 5H, C_6_H_11_-H), 1.10–1.01 (*m*, 5H, C_6_H_11_-H). **^13 ^C NMR (DMSO-d_6_, 101 MHz)**: δ 173.91(CO), 151.41(S-C), 142.90, 136.56, 121.47, 121.35, 117.58, 109.85 (Ar-C), 49.49 (C_6_H_11_-CH_2_), 37.75 (S-CH_2_), 32.45 (2-CH_2_), 31.26 (C_6_H_11_-C-1), 30.23 (C_6_H_11_-C-2,6), 25.78 C_6_H_11_-C-3,5), 25.20 (C_6_H_11_-C-4), 24.58 (3-CH_2_). **MS LCMS (ES)**: (calc) 347.17 (found) 348.2614 (MH)^+.^ Anal. Calcd for C_18_H_25_N_3_O_2_S: C, 62.22; H, 7.25; N, 12.09. Found: C, 62.53; H, 6.98; N, 12.31.

#### 2.1.15. 4-((1–(2-cyclohexylethyl)-1H-benzo[d]imidazol-2-yl)thio)-N-hydroxybutanamide(10d)

Yield: 31.0%, m.p.: 132 °C, off-white microcrystals; IR (KBr) ʋ_max_/cm^−1^: 3431 (broad band NH/OH), 1715 (CO). **^1^H NMR (DMSO-d_6_, 400 MHz)**: δ 12.21 (s, 1H), 7.53 (dd, *J* = 6.7, 2.0 Hz, 1H, 8-H), 7.48–7.41 (*m*, 1H, 5-H), 7.21–7.08 (*m*, 2H, 6,7-H), 4.12 (*t*, *J* = 7.5 Hz, 2H, C_6_H_11_-CH_2_CH_2_), 3.36–3.33 (*m*, 2H, S-CH_2_), 2.38 (*t, J* = 7.4 Hz, 2H, 2-CH_2_), 1.95 (*p*, *J* = 7.3 Hz, 2H, 3-CH_2_), 1.75–1.54 (*m*, 7H, C_6_H_11_-CH_2_CH_2_,C_6_H_11_-H), 1.29–1.10 (*m*, 4H, C_6_H_11_-H), 0.97–0.89 (*m*, 2H, C_6_H_11_-H). **^13 ^C NMR (DMSO-d_6_, 101 MHz)**: δ 173.91(CO), 150.84 (S-C), 143.04, 135.89, 121.51, 121.39, 117.63, and 109.41(Ar-C), 41.44 (C_6_H_11_-CH_2_CH_2_), 38.87 (S-CH_2_), 36.28 (C_6_H_11_-CH_2_CH_2_), 34.57(2-CH_2_), 32.58 (C_6_H_11_-CH_2_CH_2_), 32.39 (C_6_H_11_-C-1), 31.03 (C_6_H_11_-C-2,6), 25.97 (C_6_H_11_-C-3,5), 25.64 (C_6_H_11_-C-4), 24.60 (3-CH_2_). **MS LCMS (ES)**: (calc) 361.18 (found) 362.2741 (MH)^+.^ Anal. Calcd for C_19_H_27_N_3_O_2_S: C, 63.13; H, 7.53; N, 11.62. Found: C, 63.41; H, 7.18; N, 11.87.

### 2.2. Biological activity

#### 2.2.1. Histone deacetylase inhibitor assays

All enzymatic assays were conducted in BPS Bioscience Inc. in 6042 Cornerstone Court West, Ste. B, San Diego, California, USA using the biochemicals, buffers and reagents according to BPS Bioscience catalog numbers as provided in the supplementary materials.

##### 2.2.1.1. % inhibition of HDAC enzyme at 10 µM of the test inhibitor

The test compounds were dissolved in DMSO. A series of dilutions for each test compound was prepared with 10% DMSO in HDAC assay buffer and 5 µL of the final dilution (10 µM) was added to a 50 µL reaction so that the final concentration of DMSO is 1% in all of the reactions. The enzymatic reactions for the HDAC enzymes were conducted in duplicate at 37 °C for 30 min in a 50 µL mixture containing HDAC assay buffer, 5 µg BSA (bovine serum albumin), HDAC substrate (10 µM), HDAC enzyme (varied in ng/reaction according to the HDAC subtype as presented in supplementary materials) and a test compound (10 µM). After enzymatic reactions, 50 µL of 2 × HDAC Developer was added to each well and the plate was incubated at room temperature for an additional 15 min. Fluorescence intensity was measured at an excitation of 360 nm and an emission of 460 nm using a Tecan Infinite M1000 microplate reader.

HDAC activity assays were performed in duplicate. The fluorescent intensity data were analyzed using the computer software, GraphPad Prism. In the absence of the compound, the fluorescent intensity (F_t_) in each data set was defined as 100% activity. In the absence of HDAC, the fluorescent intensity (F_b_) in each data set was defined as 0% activity. The percent activity in the presence of each compound was calculated according to the following equation: % activity  = (F-F_b_)/(F_t_-F_b_), where F = the fluorescent intensity in the presence of the compound.

##### 2.2.1.2. IC_50_ of the test inhibitor against HDAC6

All of the compounds are dissolved in DMSO. The serial dilution of the compounds was first performed in 100% DMSO with the highest concentration at 1 mM. Each intermediate compound dilution (in 100% DMSO) will then get directly diluted 10x fold into assay buffer for an intermediate dilution of 10% DMSO in HDAC assay buffer and 5 µL of the dilution was added to a 50 µL reaction so that the final concentration of DMSO is 1% in all of reactions. The enzymatic reactions for the HDAC enzymes were conducted in duplicate at 37 °C for 30 min in a 50 µL mixture containing HDAC assay buffer, 5 µg BSA, an HDAC substrate (10 µM), a HDAC enzyme (10 ng/reaction) and a test compound (10 doses range from 0.0003 µM to 10 µM upon 3-fold dilution)/standard inhibitor (10 doses range from 0.00003 µM to 1 µM upon 3-fold dilution). After enzymatic reactions, 50 µL of 2 x HDAC Developer was added to each well for the HDAC enzymes and the plate was incubated at room temperature for an additional 15 min. Fluorescence intensity was measured at an excitation of 360 nm and an emission of 460 nm using a Tecan Infinite M1000 microplate reader.

HDAC activity assays were performed in duplicates at each concentration. The fluorescent intensity data were analysed using the computer software, Graphpad Prism. In the absence of the compound, the fluorescent intensity (F_t_) in each data set was defined as 100% activity. In the absence of HDAC, the fluorescent intensity (F_b_) in each data set was defined as 0% activity. The percent activity in the presence of each compound was calculated according to the following equation: % activity  = (F-F_b_)/(F_t_-F_b_), where F = the fluorescent intensity in the presence of the compound. The values of % activity versus a series of compound concentrations were then plotted using non-linear regression analysis of Sigmoidal dose–response curve generated with the equation Y = B+(T-B)/1 + 10^((LogEC50-X)×Hill Slope)^, where Y = percent activity, B = minimum percent activity, T = maximum percent activity, X = logarithm of compound and Hill Slope = slope factor or Hill coefficient. The IC_50_ value was determined by the concentration causing a half-maximal percent activity.

#### 2.2.2. Cytotoxicity assay using xCELLigence system

Established primary Choroid plexus carcinoma (CCHE-45) were cultured as previously described [41], in RPMI-1640 (Lonza, BE-12-702F) supplemented with 10% Fetal Bovine Serum (FBS, Gibco, 12483–020) and 100 U/ml penicillin and 100 µg/ml streptomycin (Lonza, DE17-602F). Cells were maintained at 37 °C in a 5% CO_2_ humidified atmosphere.

Cytotoxicity assay by the xCELLigence system was performed using RTCA xCELLigence DP system (ACEA Biosciences, Inc., San Diego, CA, USA) as per manufacturer’s instructions https://www.aceabio.com/products/rtca-dp/, https://www.aceabio.com/products/rtca-sp^65–68^. In brief, after setting up the instruments and blanking the wells with media, CCHE-45 cells were seeded into the 96 well E-plate (ACEA Biosciences) with a density of 20,000 cells/well. Attachment and growth of the cells were monitored every 1 h. Approximately 24 h after seeding, when the cells were in the log growth phase, the cells were exposed to a range of test compound **10a** concentrations (3.125, 6.25, 12.5, 25, 50, 100, 200, 400) µM for 96 h. Controls received either medium alone, or medium + DMSO with a final concentration below of 0.5%. For each concentration, duplicates were tested. Experiments were conducted in three biological repeats. Results were analyzed using the RTCA software (Version 2.0). Data was exported and average IC_50_ value was analyzed.

#### 2.2.3. Western blot analysis

CCHE-45 cells were treated with 100 µM of test compound for 24 h. HL60 cell line (a kind gift from Professor Azza Kamel from the National Cancer Institute, Cairo University); and is cultured in RPMI-1640 (Lonza, BE-12-702F) supplemented with 10% Fetal Bovine Serum (FBS, Gibco, 12483–020) and 100 U/ml penicillin and 100 µg/ml streptomycin (Lonza, DE17-602F), has also been treated with different concentrations of **10a** in twofold serial dilutions for 24 h. Proteins were extracted from CCHE-45 cells using RIPA Lysis and Extraction Buffer (ThermoScientific, cat. No: 89901) containing 100x Halt Protease and Phosphate Inhibitor Cocktail (ThermoScientific, cat. No. 78444). Followed by protein quantification using Bradford Reagent (Pierce Coomassie Plus (Bradford) Assay Reagent) (ThermoScientific, cat. No. 23238). A total of 50 µg proteins were loaded on a 10% SDS page using 30% Acrylamide/Bisacrylamide 19:1(Serva, cat. No. 10679.01) then transferred by semidry transfer method using Bio-Rad Trans-Blot Turbo transfer system on PVDF Transfer Membrane (ThermoScientific, cat. No. 88518). The membranes were incubated overnight at 4 °C in the primary antibodies Mouse monoclonal Anti-Beta Actin antibody (1:100, abcam, ab8224) and Mouse monoclonal Anti-acetylated α Tubulin (1:200, Santa Cruz Biotechnology, sc-23950). Membranes were washed 3 times using TBST then incubated for an hour at room temperature in secondary antibody Anti-Mouse IgG, HRP-linked Antibody (1:1000, Cell Signaling, cat. No. 7076). Detection take place by Clarity Western ECL Substrate (Bio-Rad, Cat. No. 170–5061) using ChemiDoc MP imaging system (Bio-Rad). Densitometry analysis was performed using ImageJ software.

### 2.3. Docking studies

Molecular docking was performed using Biovia’s Discovery studio 4.0 software using the Dock ligands (CDOCKER) protocol which is an implementation of the CDOCKER algorithm. The X-ray crystal structure of the kinase domain of HDAC6 in complex with its propionic acid derivative inhibitor (PDB entry 5G0H) was recovered from RSCB protein data bank. The protein structure was prepared using protein preparation protocol of Biovia’s discovery studio 4.0. The amino acid residues were ionised using role-based technique and the missing residues and hydrogen atoms were added and minimised. The protein structure was typed by CHARMM. Synthesised compounds were prepared from ligands prepare tool which fix bad valences, adds hydrogen, and generates a 3 D coordinates using catalyst. Docking was performed using (CDOCKER) protocol. Top hits were set to 10 and pose cluster radius was set to 0.5 Å, while other docking parameters were kept as default. The best docking poses are analyzed according to docking score and interactions with key amino acids of the receptor using CDOCKER_ENERGY scoring function.

## 3. Results and discussion

### 3.1. Chemistry

**Scheme 1.**
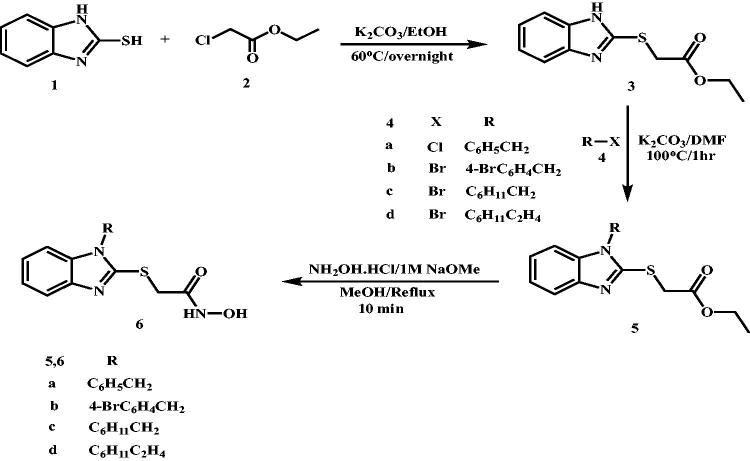
Synthesis of (benzimidazol-2-yl)thio-*N*-hydroxyacetamide derivatives (**6**).

The target product (benzimidazol-2-yl)thio-*N*-hydroxyacetamide derivatives **(6a-d)** were synthesised as shown in Scheme 1 starting from 2-mercaptobenzimidazole **(1)** to be alkylated with ethyl chloroacetate **(2)** in ethanolic suspension of potassium carbonate (K_2_CO_3_) at 60 °C to afford the corresponding ester **(3)** in 34% yield after stirring for 4 h. The resulting thioacetate ester **(3)** without further crystallisation, underwent an alkylation process that involves alkylation of benzimidazole-NH with 1.0 equivalent of benzyl chloride **(4a)**, 4-bromobenzyl bromide **(4b)**, cyclohexylmethyl bromide **(4c)** and cyclohexylethyl bromide **(4d)**. The alkylation reaction was conducted in DMF in the presence of 1.5 equivalent of K_2_CO_3_ at 100 °C for 1 h to afford the respective ethyl 1-substituted(benzimidazole-2-yl)thioacetate esters **(5a-d)** in reasonable yields (40%-65%). The ester products **(5a,c,d)** were isolated as oil products while 4-bromobenzyl derivative **(5b)** was crystallised from methanol as golden yellow needles. The resulting esters **(5a-d)** were used for the next step without further chromatographic purification or crystallisation.

Finally, The hydroxylamine hydrochloride (10 equivalent) was neutralised with 1 M NaOMe (14 equivalent) in methanolic solution and the produced hydroxylamine was promptly added to the respective ethyl 1-substituted(benzimidazole-2-yl)thio acetate esters **(5a-d)** (1.0 equivalent) dissolved in methanol. The reaction was considered complete after 10 min of stirring under reflux via observing disappearance of the ester spot on thin layer chromatography (TLC). The hydroxamic acid products (**6b, 6c, 6d**) were obtained in low yields while the benzyl derivative **(6a)** had the highest yield of 22%.

The structures of *N*-hydroxyacetamide derivatives **(6a-d)** were confirmed by IR, ^1^H-NMR, ^13 ^C-NMR, Mass spectrometry and microanalysis. Furthermore, ^1^H-NMR confirmed the generation of S-acetate ester regioisomer not *N*-acetate ester. In ^1^H-NMR spectrum, Compound **6b** as a representative figure to the whole series **(6a-d)** showed a singlet proton peak at chemical shift 4.14 ppm for methylene proton of *S*-C**H_2_**CONHOH while the reported^69^ value for methylene protons of *N*-C**H_2_**CONHOH is 4.98 ppm which verify the successful synthesis of the target regioisomers **(6a-d)**.

**Scheme 2.**
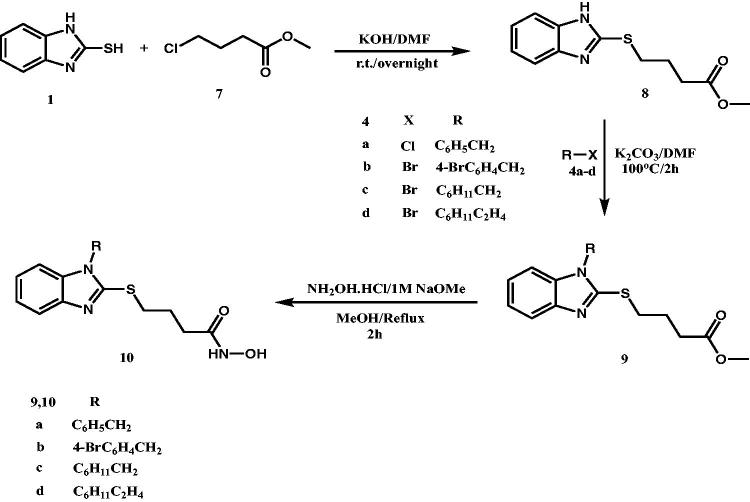
Synthesis of (benzimidazol-2-yl)thio-*N*-hydroxybutanamide derivatives (**10**).

On the other hand, we used different reaction conditions for preparation of the second series of target HDAC6 inhibitors (benzimidazol-2-yl)thio-*N*-hydroxybutanamide derivatives **(10a-d)** starting from 2-mercaptobenzimidazole **(1)** as shown in Scheme 2. Methyl 4-chlorobutyrate **(7)** was used to alkylate 2-mercaptobenzimidazole at position 2, potassium hydroxide was used as strong base to enhance the formation of thiolate salt in DMF as polar aprotic solvent to potentiate the efficacy of alkylation process with 4-carbon chain alkylating agent by stirring overnight at room temperature. The starting ester, methyl 4-((1*H*-benzo[*d*]imidazol-2-yl)thio) butanoate **(8)** was obtained in 76% yield and in pure needle crystals. Because the reaction condition was very regioselective, there were no any by-products require further purification. Interestingly, the resulting ester **(8)** gave positive hits on searching SciFinder for its preparation. But upon searching the references contents, we did not find the methyl ester of the prepared compound **(8)** but only the ethyl ester^70^. The ethyl ester counterpart of **(8)** was prepared using triethylamine as a mild base in DMF by stirring for 12 h at 80 °C and it was purified by column chromatography to infer that upon heating for such long period of time, multiple alkylation might have happened with these reaction conditions.

The prepared ester **(8)** was heated with 1.0 equivalent of benzyl chloride **(4a)**, 4-bromobenzyl bromide **(4b)**, cyclohexylmethyl bromide **(4c)** and cyclohexylethyl bromide **(4d)** in the presence of 1.5 equivalent of K_2_CO_3_ in DMF at 100 °C for 2 h to afford the corresponding *N*-benzyl, *N*-(4-bromobenzyl), *N*-(cyclohexylmethyl) and *N*-(cyclohexylethyl) esters **(9a-d)** respectively, in quantitative yields.

The oil products of methyl 4-((1-benzyl/cycloalkyl-1*H*-benzo[*d*]imidazol-2-yl)thio)butanoates **(9a-d)** were used to prepare the corresponding *N*-hydroxybutanamide derivatives **(10a-d)** upon its treatment with hydroxylamine in methanol at room temperature after stirring for 2 h as described previously for synthesis of *N*-hydroxyacetamide derivatives **(6a-b)**. The target compounds were obtained in yields (25%–35%).

The structures of the final *N*-hydroxybutanamide derivatives **(10a-d)** were confirmed by IR, ^1^H-NMR, ^13 ^C-NMR, mass spectrometry and microanalysis. In ^1^HNMR, appearance of triplet proton peak of methylene group of *S*-C**H_2_**CH_2_CH_2_CONHOH for compound **10c** at chemical shift 3.35 ppm while the reported value^69^ for methylene protons of *N*-C**H_2_**CH_2_CH_2_CONHOH is 4.28 ppm, confirmed the synthesis of target regioisomers **(10a-d)**.

### 3.2. Biological activity

#### 3*.2.1. Human HDAC inhibition activity*

Activity of the synthesised benzimidazole-based hydroxamic acid derivatives **(6a-d)** and **(10a-d)** against HDACs isoforms via *in vitro* enzymatic assay was investigated. The study revealed that the derivatives of one-carbon linker **(6a-d)** performed weak inhibiting activity against several HDAC isoforms including HDAC6 (Table 1). Among the investigated derivatives of three-carbon linker **(10a-d)**, 1-benzylbenzimidazolyl-*N*-hydroxybutanamide **10a** showed excellent inhibiting activity at 10 µM (92%) against HDAC6. Moreover, **10a** exhibited impressive preferential activity against HDAC6 when compared to the inhibiting activity of the same derivative against all the other isoforms that represent class I (HDAC1, HDAC2, HDAC3, HDAC8), class IIa (HDAC4, HDAC5, HDAC7, HDAC9), class IIb (HDAC10) and class IV (HDAC11) (Table 1). There was no need to measure the corresponding IC_50_ values^71^ of **10a** against other HDAC isoforms due to the weak inhibiting activity that didn’t reach 75% against any isoform (Table 1). The % inhibition was enough to highlight **10a** that showed significant preference against HDAC6. Moreover, the other *N*-hydroxybutanamide derivatives were with insignificant activity against other HDAC isoforms as shown in (Table 1). The % inhibition results promoted **10a** among all the synthesised derivatives to measure its HDAC6 IC_50_ value that specified the corresponding potency at 510 nM. In accordance, the nanomolar potency of **10a** could primarily define it as new-preferential HDAC6 lead inhibitor ^34^^,^^72–74^.

**Table 1.** *In vitro* inhibition activity of test compounds (6a-d) and (10a-d) against human HDACs.

Compound	%inhibition of HDACs at 10µM of test compound^a^											IC_50_±SE^b^
	HDAC1	HDAC2	HDAC3	HDAC4	HDAC5	HDAC6	HDAC7	HDAC8	HDAC9	HDAC10	HDAC11	HDAC6
**6a**	6	5	ND^c^	ND	ND	2	ND	14	ND	ND	ND	ND
**6b**	18	6	ND	ND	ND	6	ND	12	ND	ND	ND	ND
**6c**	13	7	ND	ND	ND	2	ND	14	ND	ND	ND	ND
**6d**	14	3	ND	ND	ND	2	ND	1	ND	ND	ND	ND
**10a**	36	19	37	1	4	92	2	56	5	28	6	510 ± 0.015nM
**10b**	9	9	ND	ND	ND	12	ND	1	ND	ND	ND	ND
**10c**	9	8	ND	ND	ND	14	ND	12	ND	ND	ND	ND
**10d**	11	8	ND	ND	ND	35	ND	10	ND	ND	ND	ND
**SAHA (1µM)**	98^d^	85	94	ND	ND	97	ND	ND	ND	97	ND	ND
**TSA (10µM)**	ND	ND	ND	64	64	100^e^	78	89	60	ND	49	5.0 ± 0.00015 nM

a Mean value of two replicates of %inhibition of HDAC at 10 µM of test compound.

b Mean value of two replicates of the concentration of test compound required to produce 50% inhibition of HDAC6 in nM**±**standard error.

c Not determined.

d Mean value of two replicates of % inhibition of HDAC1 at 3 µM of SAHA.

e Mean value of two replicates of % inhibition of HDAC6 at 1 µM of TSA.

#### 3.2.2. Cytotoxic activity assay against choroid plexus carcinoma (CCHE-45) cells

**Figure 4.**
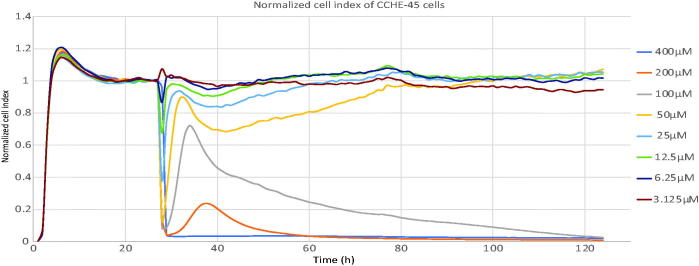
Real-time cell analysis of the cytotoxic effect of **(10a)** on CCHE-45. Cells were seeded into the E-plate then 24 h later, treated with a range of concentrations of (**10a)** for 96 h. Graph is showing CCHE-45 cell response profiles designated as cell index for the different concentrations of (**10a)** over 96 h. Graph is a representative of three independent experiments. Concentrations are 400 µM (blue line), 200 µM (orange line), 100 µM (gray line), 50 µM (yellow line), 25 µM (light blue line), 12.5 µM (green line), 6.25 µM (dark blue line), 3.125 µM (brown Line).

Choroid plexus carcinoma cell line CCHE-45 was then used to test the effect of the test HDAC6 inhibitor **10a** on cell proliferation. CCHE-45 cell line is characterised by constitutive formation of aggresomes^41^, which are inclusion bodies for highly misfolded proteins formed by the collapse of the intermediate filament vimentin. Protein aggregates that are unable to be degraded by the proteasome are shuttled along the microtubules to the aggresomes assisted by dynein motor proteins and HDAC6^75^. Due to the essential role of HDAC6 in aggresomes formation, CCHE-45 cell line was chosen to test the cytotoxic activity of HDAC6 inhibitor **10a**. xCELLigence system was used to allow real-time monitoring of cell response to the drug; without any need for toxic labeling. The monitoring of cell proliferation for 96 h (Figure 4) identified the concentration of 400 µM or 200 µM of **10a** led to a quick and dramatic reduction in cell index (CI), while 100 µM reduced CI to half the maximum CI after 24 h from addition of the drug with a mean IC50 of 112.67 ± 11.06 µM (Table 2).

**Table 2.** *In vitro* cytotoxic activity of (**10a**) against CCHE-45.

Compound	CCHE-45 IC_50_ (µM)±SEM^a^
**10a**	112.67 ± 11.06
**Tubacin**	20.00 ± 10.18

a Mean value of three replicates of the required concentration of test compound to produce 50% inhibition of CCHE-45cells **±** standard error of the mean.

When (CCHE-45 IC50 = 112.76 µM) of **10a** was compared to that of Tubacin; a standard HDAC6 selective inhibitor (CCHE-45 IC50 = 20 µM) (Table 2), it showed that the new lead inhibitor has just 5 times lower potency than the standard inhibitor. However, when compared to their enzymatic activity, where Tubacin has originally an HDAC6 IC_50_  = 4 nM^55^ which is 127.5 times more potent than the lead inhibitor **10a** (HDAC6 IC_50_  = 510 nM) to infer that the new lead inhibitor carries remarkable efficacy and interesting cytotoxic profile. Accordingly, it is highly anticipated that the future structural optimisation of the lead inhibitor **10a** might result in a selective HDAC6 inhibitor with a superior potency against CCHE-45 cells, compared to Tubacin.

#### 3.2.3. Cell-based assay of acetylated α-tubulin

**Figure 5.**
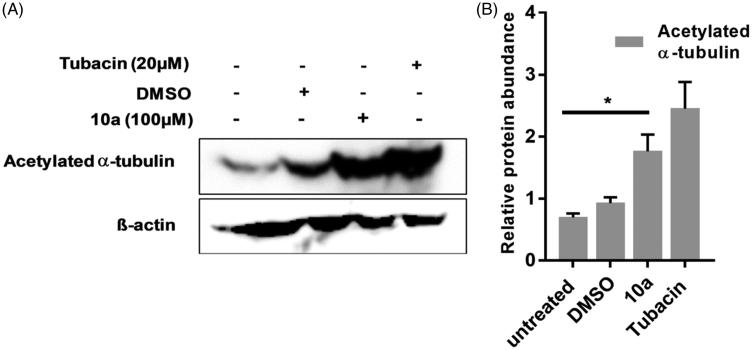
The effect of **(10a)** on the acetylation of α-tubulin. Treatment of CCHE-45 cells for 24 h with 100 µM of **(10a)** upregulated acetylated α-tubulin using Tubacin as standard HDAC6 inhibitor at 20 µM. (**A**) Representative Western Blot analysis of acetylated α-tubulin protein levels of three replicates showing the effect of **(10a)** on acetylation of α-tubulin. (**B**) Relative mean densitometry measurement value of protein abundance levels using ImageJ software. β-Actin was used as a loading control.

Western Blot analysis of acetylated α-tubulin was used to detect and semi-quantify the upregulation of acetylated α-tubulin that accumulates upon inhibition of HDAC6 by **10a** treatment. A significant increase in acetylated α-tubulin was observed in CCHE-45 cells at 24 h after treatment with **10a** (100 µM), which is statistically equivalent to the levels of acetylated α-tubulin induced by Tubacin addition (20 µM) (Figure 5(A)). This increase in acetylated α-tubulin was consistent through three biological replicates. Interestingly, the on-target mechanism of antiproliferative activity of **10a** against brain cancer cells CCHE-45 as HDAC6 inhibitor was confirmed via exhibition of insignificant difference between the test inhibitor **10a** and Tubacin according to densitometry measurements (Figure 5(B)) at their corresponding CCHE-45 IC_50_ values, (Table 2). Moreover, these results excluded the possibility of off-target mechanism of action of **10a** against brain cancer cells CCHE-45 and the new inhibitor doesn’t have multiple targets by which it caused cancer cell death. This observation might highlight the new lead compound **10a** as safe inhibitor with controlled biological interference as antiproliferative agent.

**Figure 6.**
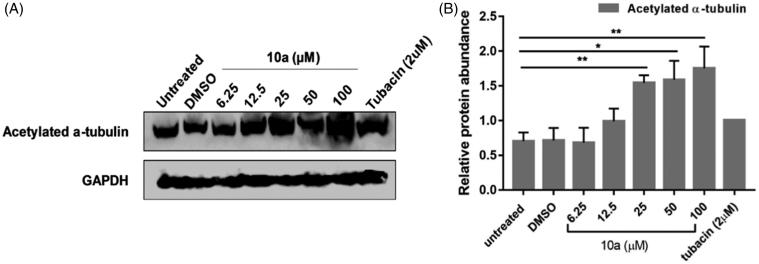
The effect of **(10a)** on the acetylation of α-tubulin. Treatment of HL60 cells for 24 h with several concentrations of **(10a)**; 6.25, 12.5, 25, 50 and 100 µM upregulated acetylated α-tubulin at concentration starting from 12.5 µM. (**A**) Representative Western Blot analysis of acetylated α-tubulin protein levels of three replicates showing the effect of **(10a)** on acetylation of α-tubulin. (**B**) Relative mean densitometric value of protein abundance levels using ImageJ software. GAPDH was used as a loading control.

We have extended the work to determine the effect of **10a** on HL60; an acute promyeloblastic leukemia cell line, as HDAC6 has been observed to be overexpressed in acute myeloid leukemia^76^^,^^77^. Treating HL60 with the test inhibitor **10a** was done through the addition of a range of concentrations over 2-fold serial dilutions starting from 100 µM and ended at 6.25 µM. According to the results shown (Figure 6(A)), acetylated α-tubulin levels were positively correlated with the test compound concentration, with a significant upregulation of acetylated α-tubulin detected at 25 µM (Figure 6(B)). This suggests that HL60 might be more sensitive to **10a** compared to CCHE-45; as much lower concentration was able to induce the acetylation of α-tubulin.

Conclusively, the enzymatic, cytotoxic activity and cell-based assays’ results could define benzimidazole-based hydroxamic acid derivative **10a** of the new class as preferential HDAC6 lead inhibitor according to Graham Patrick’s definition^74^ of the new lead compound in drug discovery studies that says “the lead is the compound that exhibits therapeutic usefulness and the level of activity is not crucial”. Thus, the newly generated lead **10a** is therapeutically useful lead for future development to improve the potency besides the selectivity, and eventually elaborate potent antiproliferative agent against solid tumour of choroid plexus carcinoma in addition to acute promyeloblastic leukaemia. Noteworthy to mention is that the generated lead inhibitor **10a** gained its importance from being the first preferential HDAC6 inhibitor with verified cytotoxic activity against brain cancer CCHE-45 as type of serious solid tumours having such interesting profile and verified on-target cytotoxic activity when compared to tubacin standard inhibitor.

### 3.3. Docking studies

**Figure 7.**
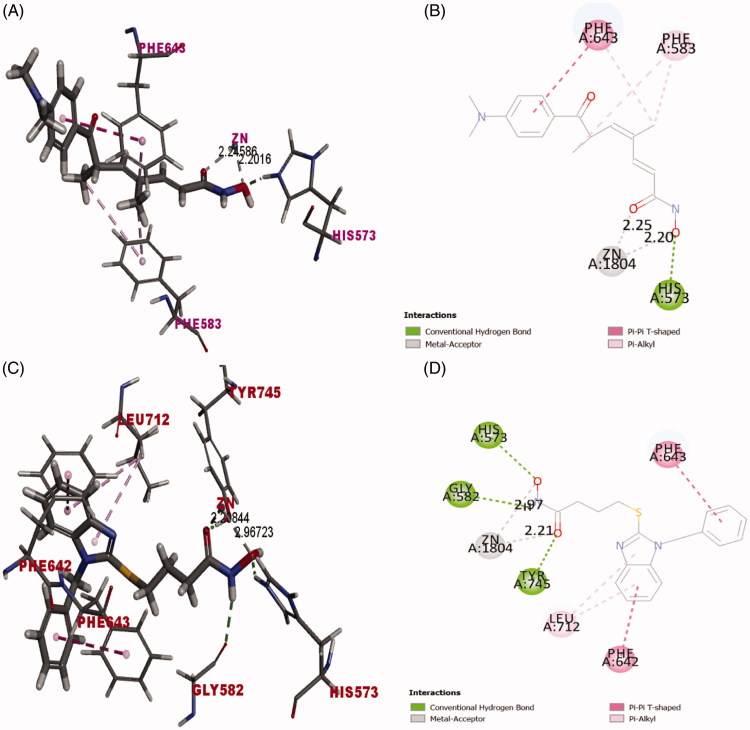
(A) Redocking of TSA (was built as solid stick model) into active biding site of HDAC6 (PDB entry 5G0H) in 3 D style and atoms are assigned by colors; (blue nitrogen, red oxygen and gray carbon). π-π stacking between the benzene ring of TSA and benzene rings of active binding site amino acid residues presented as pink dotted line. Alkyl-π stacking between alkyl chain of TSA and benzene rings of amino acid residues presented as light pink dotted line. Zinc metal ion-TSA coordinate bond formation presented as grey-dotted line. (B) Docking solution of TSA into the active binding site of HDAC6 (PDB entry 5G0H) in 2D style (C) Docking solution of compound **(10a)** (was built as solid stick model) into the active binding site of HDAC6 (PDB entry 5G0H). Yellow sulphur atom and hydrogen bond formation between compound (**10a)** and the amino acid residues represented as green dotted line, the description of the rest of binding interaction representations are the same as A and B. (D) Docking solution of compound **(10a)** into the active binding site of HDAC6 (PDB entry 5G0H) in 2D style.

A molecular docking study using discovery studio 4.0 http://www.3dsbiovia.com/events/webinars/discovery-studio-25/index.html was performed using the Dock ligands (CDOCKER) protocol^78^. It is used to visualise the binding modes and orientation of some representative examples of the synthesised compounds into the active binding site of HDAC6. The coordinates of the target protein structure were obtained from the crystal structure of HDAC6 (PDB entry 5G0H)^51^ in complex with the preferential HDAC6 ligand inhibitor Trichostatin A (TSA) that shows high similarity to the human orthologue^79^. Validation of docking algorithm was achieved by redocking of co-crystallised structure of the inhibitor inside the active binding site of HDAC6. The root mean square difference (RMSD) between the top docking pose and original crystallographic geometry was 0.44 Å. This provides sufficient confidence in drawing meaningful conclusions from the docking study. In addition, redocking of TSA retrieved the reported binding mode of the inhibitor into the X-ray crystal structure of the active binding site of HDAC6 (C-docker energy  = −25.49) as depicted in (Figure 7(A,B)), where the carbonyl and hydroxyl oxygens of hydroxamate moiety complexes with the corresponding Zn^2+^ metal ion in a bidentate fashion at metal-coordinate bond distances 2.25 Å and 2.20 Å, respectively (Figure 7(A,B)). The unsaturated aliphatic linker of TSA was nearly planar and sandwiched between the aromatic side chains of Phe583 and Phe643. Whereas the carbonyl group of the hydroxamate was almost coplanar with the unsaturated aliphatic chain and appeared twisted by ∼30° towards the zinc metal ion. The hydroxyl oxygen of hydroxamate makes hydrogen bonding with His573. Phenyl radical of docked ligand inhibitor TSA interacted with Phe643 via π-π stacking while the unsaturated aliphatic chain of the linker showed Alkyl-π interaction with Phe583 and Phe643 (Figure 7(A,B)).

The binding mode of the synthesised compounds of benzimidazole-based derivatives was investigated to justify the nanomolar activity of compound **10a** (IC_50_  = 510 nM) against HDAC6 among all the other derivatives **(5a-d)** and **(10b-d)** that showed remarkably low %inhibition against HDAC6 compared to **10a** (Table 1). On the other hand, identifying the binding mode of some representative examples of low-active benzimidazole derivatives **6a** and **10b** will provide us reliable clues for future development and optimisation of the identified lead inhibitor **10a**.

Docking of 1-benzylbenzimidazolyl-*N*-hydroxybutanamide **10a** into the catalytic domain of HDAC6 (PDB entry 5G0H) revealed the (C-docker energy  = −39.12 Kcal/mol) to be superior to that of ligand inhibitor, TSA (C-docker energy  = −25.49 Kcal/mol). Binding interactions of **10a** with the active binding site amino acid residues showed formation of three hydrogen bonds between hydroxamic acid and His573, Tyr745 and Gly582, π-π stacking between 1-benzyl radical and Phe643. Moreover, benzimidazole ring interacted with Phe642 via π-π stacking and with Leu712 via alkyl-π stacking (Figure 7(C,D)). Carbonyl and hydroxyl oxygens of hydroxamate moiety captured zinc metal ion in bidentate manner at respective bond distances 2.21 Å and 2.97 Å **(**Figure 7(C,D)) to imply the formation of metal-coordinate bond with lower strength due to the longer bond length^80–82^ when compared to TSA (Figure 7(A,B)). It is worthy to emphasise that the generated lead inhibitor **10a** succeeded to interact with 3 amino acids; Phe643, Gly582, Leu712 out of 5 amino acids [51] that constitute the narrow hydrophobic channel of HDAC6 at which the acetylated lysine is set into the catalytic domain for deacetylation while the ligand inhibitor TSA interacted with only Phe643. In addition, **10a** formed hydrogen bond as an extra type of interaction with Tyr745 that TSA in its bioactive conformer was not able to form (Figure 7(B–D)). Tyr745 is located next to zinc metal cation and thought to stabilise the transition state of intermediate to eventually release the product with deacetylated lysine residues^51^.

**Figure 8.**
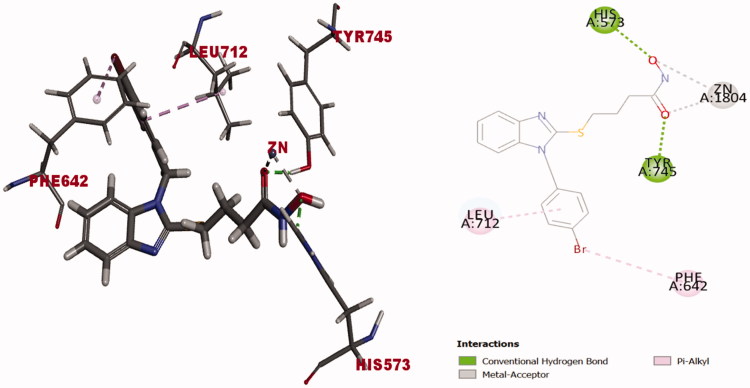
Docking solution of (**10b)** (was built as solid stick model) into catalytic domain of HDAC6 (PDB entry 5G0H) in 3D style in the left side and 2D style in the right side. Atoms are assigned by colours; (blue nitrogen, red oxygen, grey carbon, yellow sulphur and maroon bromine). Alkyl-π stacking presented as light pink dotted line. Zinc metal cation-hydroxamate coordinate bond formation presented as grey-dotted line. Hydrogen bond formation between compound (**10b)** and the amino acid residues represented as green dotted line.

Compound **10b**; the1–(4-bromobenzyl) counterpart of the lead inhibitor **10a** that with 4-atoms-linker, was chosen to represent the derivatives of low inhibition activity (12%) against HDAC6 (Table 1). Docking solution of **10b** into the catalytic domain of HDAC6 (PDB entry 5G0H) showed (C-docker energy  = −36.34 Kcal/mol) to excel the binding affinity of ligand inhibitor TSA (C-docker energy  = −25.49 Kcal/mol). Upon investigating the real reason(s) that lied behind the discrepancy in activity between **10b** and 1-benzyl derivative **10a** against HDAC6, it was found that the orientation of the docked compound **10b** flipped around the S-alkyl chain axis (Figure 8) when compared to **10a** (Figure 7(C)). This flip oriented the 4-bromobenzyl fragment upward in the hydrophobic channel to interact with Leu712 via alkyl-π stacking and be away from Phe643. Benzimidazole ring was then oriented downward to interact with Phe642 via π-π stacking. This might be attributed to the para substitution of 1-benzyl ring with bromine that led to steric clash with Phe643 forced the 4-bromobenzyl to flip and stay closer to Phe642. 4-Bromobenzyl exhibited π-stacking with Phe642 and Leu712 via bromine substituent and benzyl ring respectively (Figure 8) and lost the ability to interact with the hydrophobic channel amino acid Phe643. The hydroxamate moiety captured the metal cation in bidentate manner and formed two hydrogen bonds with His573 and Tyr745 (Figure 8). Thus, significant weaker inhibition activity of **10b** might be attributed to the loss of π-π stacking interaction with Phe643.

**Figure 9.**
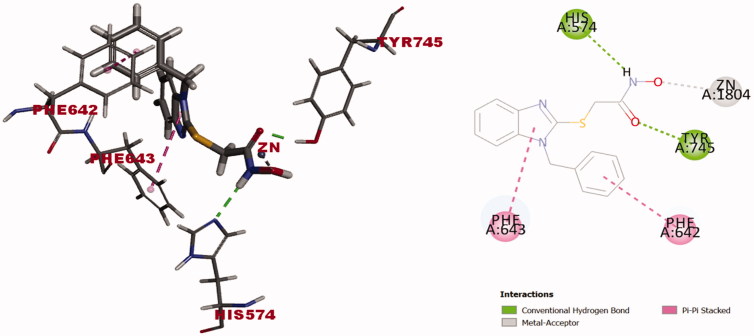
Docking solution of **(6a)** (was built as solid stick model) into catalytic domain of HDAC6 (PDB entry 5G0H) in 3 D style in the left side and 2D style in the right side. Atoms are assigned by colours; (blue nitrogen, red oxygen, yellow sulphur and grey carbon). π-π stacking presented as pink dotted line. Zinc metal cation-hydroxamate coordinate bond formation presented as grey-dotted line. Hydrogen bond formation between compound **(6a)** and the amino acid residues represented as green dotted line.

Finally, we docked the 1-benzylbenzimidazole-*N*-hydroxyactamide derivative **6a**; the one-carbon linker counter part of **10a** that showed insignificant inhibiting activity (2%) against HDAC6 (Table 1) compared to **10a** (92%). The docking solution of **6a** into the active binding site of HDAC6 (PDB entry 5G0H) showed comparable binding affinity (C-docker energy  = -28.63 Kcal/mol) to ligand inhibitor TSA (C-docker energy  = −25.49 Kcal/mol) but lower than that of **10a** (-39.12 Kcal/mol) and **10b** (−36.34 Kcal/mol) to reflect significant alteration in the binding interaction forces with the hydrophobic channel amino acid residues of the catalytic domain. The orientation of the docked derivative **6a** left an impression that the shortness of the linker made the compound struggle to take the pose that fulfills all the required binding interactions for successful inhibition to the enzyme. This could be justified by the resulting best pose of **6a** that flipped on the S-alkyl chain axis to let benzimidazole ring catch the opportunity to interact with Phe643 via π- π stacking and it gives the hydroxamate moiety that built on short alkyl chain, the chance to face the zinc metal ion (Figure 9). The docked compound **6a** also swung a little to get the hydroxamate residue closer to zinc metal ion to capture it via formation of metal-coordinate bond by the hydroxyl oxygen in a monodentate fashion (Figure 9). Thus, test compound’s flipping led the1-benzyl group to interact with Phe642 via π- π stacking and lost the interaction with Leu712 as one of the hydrophobic channel amino acid residues when compared to *N*-hydroxybutanamide **10b** case. Two further hydrogen bonds are formed between the hydroxamate moiety and His574 and Tyr745 amino acid residues (Figure 9). Conclusively, the insignificant activity of 1-benzylbenzimidazole-*N*-hydroxyactamide **6a** is definitely attributed to shortness of the linker between the 1-benzylbenzimidazole hydrophobic cap and the hydroxamate moiety.

Based on the results of the above investigation that involved docking the compound **10a** of the most inhibition activity (92%) against HDAC6 (IC_50_ = 510 nM) and two representative examples of insignificant inhibiting activity **10b** (12%) and **6a** (2%), it was obvious that *the length of the linker*, *interaction of the 1-(4-unsubstituted)benzyl hydrophobic cap of benzimidazole ring with the narrow hydrophobic channel amino acid residues; Phe643 and Leu712 via π-stacking* and *bidentate capture of the hydroxamate moiety to zinc metal ion by the aid of carbonyl and hydroxyl oxygens* are critical determinants that verified the inhibiting activity pattern of the test inhibitors generated from such benzimidazole skeleton. This also justifies the weak inhibiting activity of 1-cyclohexyl derivatives **6c,d** and **10c,d** that lack the interaction with the hydrophobic channel amino acids of HDAC6 via π-π stacking which is considered a critical type of interaction for successful inhibition of HDAC6.

After exhibiting the binding mode of the generated lead inhibitor; 1-benzylbenzimidazole-*N*-hydroxybutanamide **10a** into the catalytic domain of HDAC6 and comparing it to that of the ligand inhibitor TSA, it was shown that the test inhibitor **10a** was with superior binding affinity due to more interaction types with the narrow hydrophobic channel amino acid residues.

This raises the question: *why the lead inhibitor****10a****that showed better binding mode and affinity to the ligand inhibitor TSA was much less potent in biological evaluation.* The lead inhibitor **10a** carries hydrophobic cap that is large enough to maintain the preferential and inhibiting activity against HDAC6 compared to HDAC1-5 and HDAC7-11 (Table 1) but is not enough to excel the selectivity. Moreover, the two critical factors that determine the activity and selectivity of the HDAC inhibitors are the hydrophobic cap and the linker^64^^,^^83^^,^^84^. Those are the two fragments that extensively varied in most of the designed HDAC inhibitors to either generate new inhibitor or to optimise the discovered lead inhibitor for improvement of the relative potency^12^^,^^13^^,^^31^. Since then, it might be that the three-carbon length of the linker of **10a** was not enough to capture the zinc metal ion in HDAC6 catalytic domain as strong as TSA due to formation of metal-coordinate bond at longer distance (Figure 7(B,D)).

Definitely, the interesting results of the docking study could provide us with an excellent framework for setting up the future directions towards optimisation of the identified lead inhibitor **10a**. The suggested directions involve: (i) longer carbon linker from five-carbon to seven-carbon to enhance the potency against the enzyme, (ii) bigger size of 1-arylmethyl residue via trying with binuclear arylmethyl residues instead of benzyl residue that is expected to change the profile of **10a** from preferential to selective inhibitor and (iii) pyridylmethyl and quinolinyl methyl via trying with heterocycle substitute to benzyl residue that might enhance the affinity and the corresponding potency by nitrogen heteroatom.

## Conclusion

The present study introduced molecular-, structural-based design and identification of new class of benzimidazole-based hydroxamic acid that involves a lead inhibitor with HDAC6 preferential inhibiting activity (HDAC6 IC_50_  = 510 nM) and on-target cytotoxic mechanism of action against CCHE-45 children brain cancer cells at (CCHE-45 IC_50_  = 112.76 µM). The generated lead inhibitor though low potency, it showed moderated cytotoxic activity against the CCHE-45 cells with interesting profile when compared to Tubacin as standard inhibitor that verified the better efficacy and the superior activity is anticipated upon enhancing the potency. The preferential inhibitor gave better activity against acute leukaemia cells HL60 at 25 µM according to Western analysis. The new class is feasible to synthesise with laboratory friendly reaction conditions and accessible to develop and modify to enhance both the potency and selectivity. Docking studies gave clues for the appropriate modifications to develop the structural features of the lead in order to change the profile from preferential to selective inhibitor. Referring to docking results, it has been recommended that introducing bigger size of arylmethyl and heterocycle substituents at position 1 of the lead scaffold will be in the favor of improving the selectivity and the longer carbons of the linker at position 2 might play a key role in enhancing the potency. For future study, these recommendations aim to enhance the level of activity and selectivity without compromising the therapeutic usefulness exhibited by the original lead inhibitor and ensure the on-target activity of the same inhibitor that may define it as safe lead inhibitor.

## Supplementary Material

Supplemental Material
