# Supplementary material for: Structure-based design generated novel hydroxamic acid based preferential HDAC6 lead inhibitor with on-target cytotoxic activity against primary choroid plexus carcinoma
Source: J Enzyme Inhib Med Chem. 2019 May 9;34(1):1062–77. doi: 10.1080/14756366.2019.1613987 (PMC6522981; doi:10.1080/14756366.2019.1613987)
Supplement: Supplemental Material [file IENZ_A_1613987_SM6743.pdf]

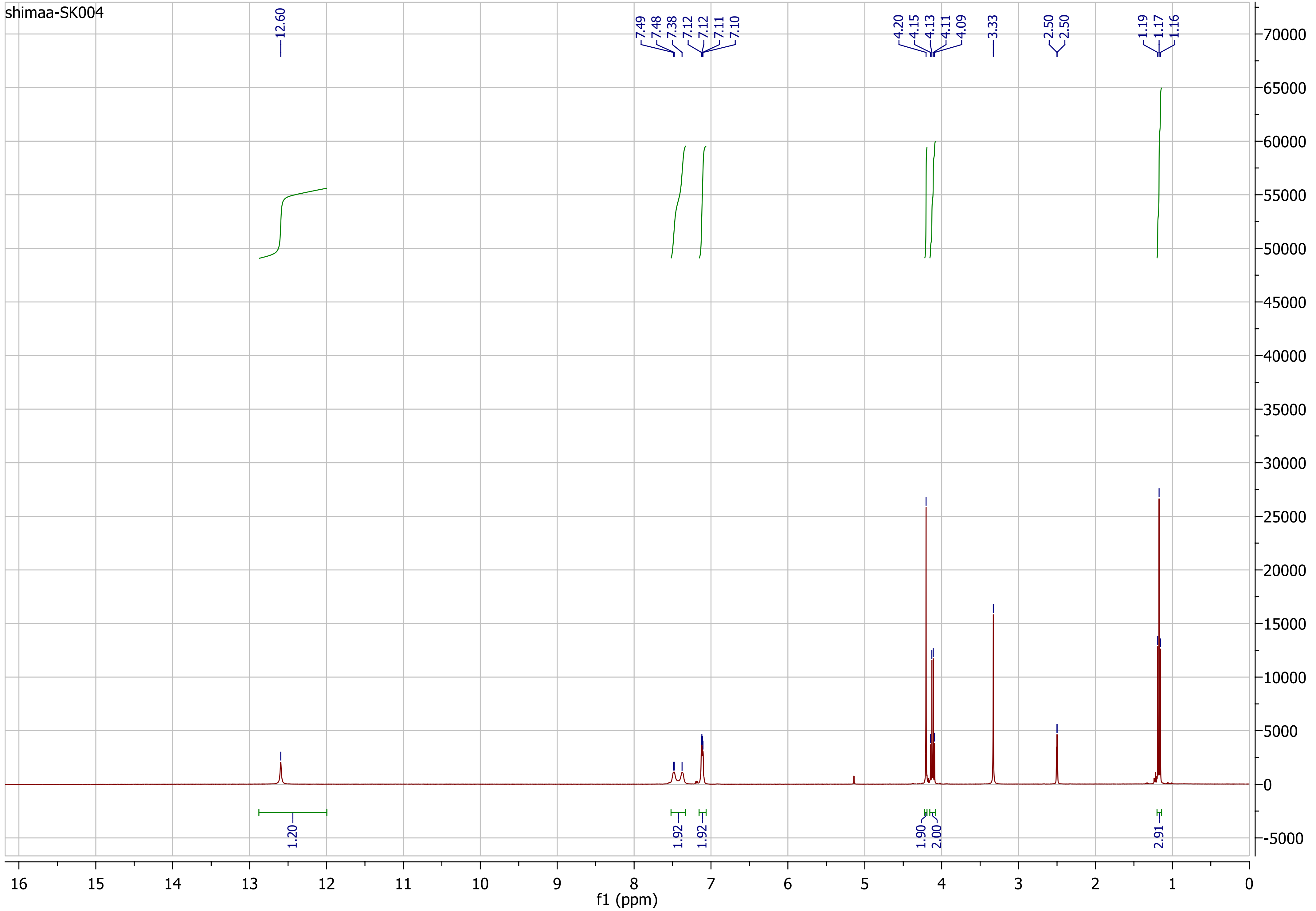
shimaa-SK004
12.60
7.49
7.48
7.38
7.12
7.12
7.11
7.10
4.20
4.15
4.13
4.11
4.09
3.33
2.50
2.50
1.19
1.17
1.16
1.20
1.92
1.92
1.90
2.00
2.91
f1 (ppm)

Shimaa Kassab-SK041-S-proton
Shimaa Kassab-SK-041-S-proton

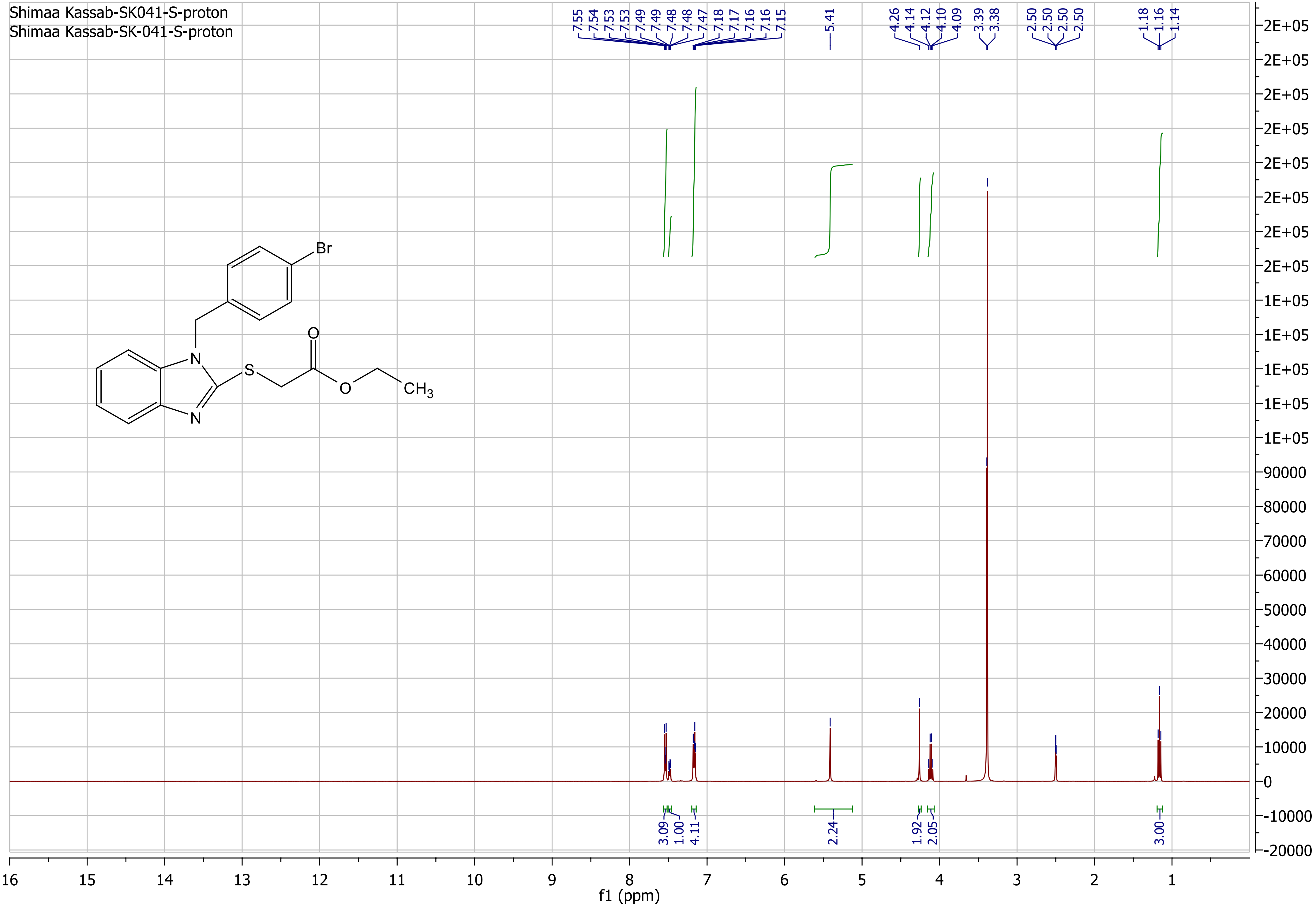

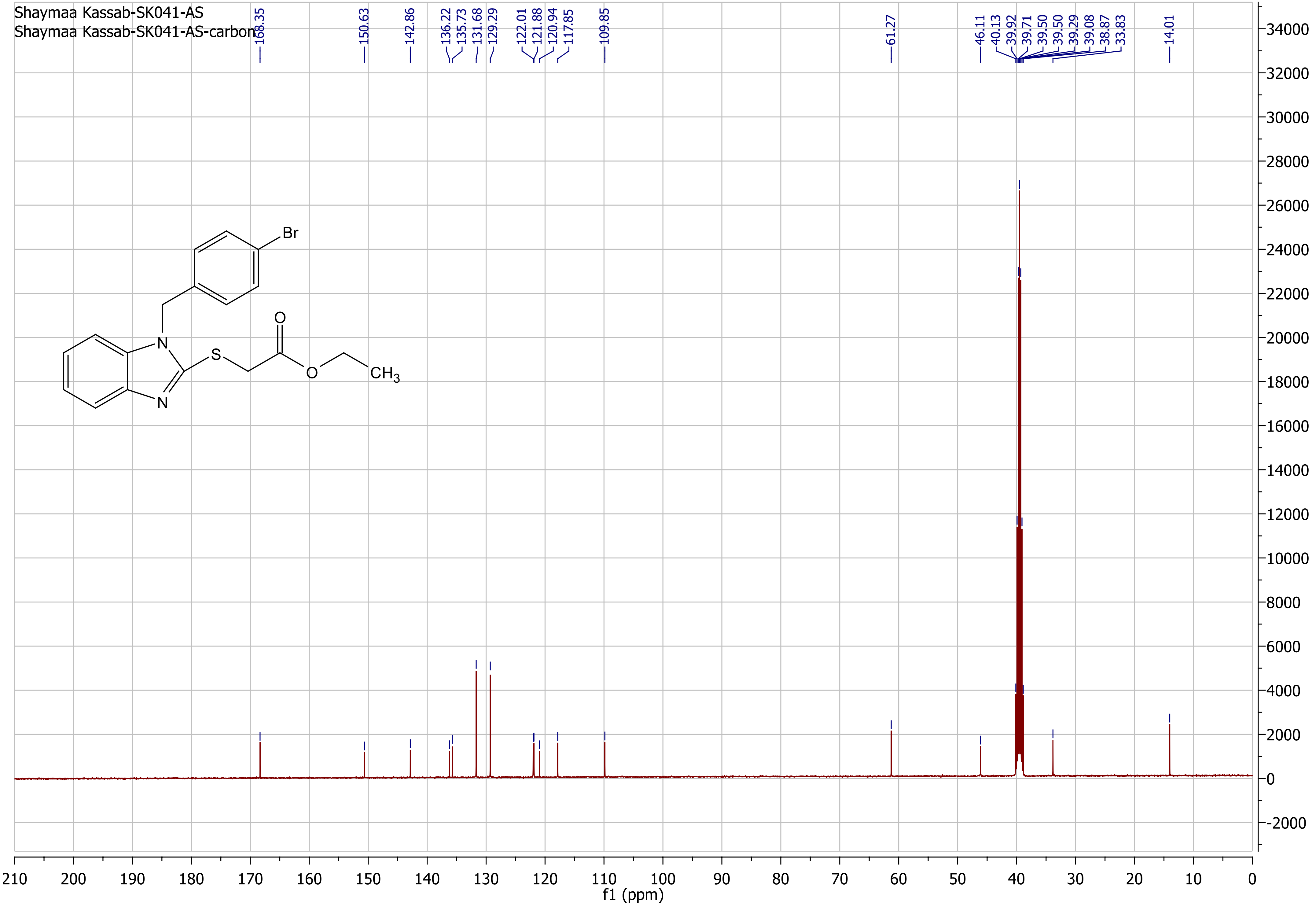
Shaymaa Kassab-SK041-AS
Shaymaa Kassab-SK041-AS-carbon
168.35
150.63
142.86
136.22
135.73
131.68
129.29
122.01
121.88
120.94
117.85
109.85
61.27
46.11
40.13
39.92
39.71
39.50
39.50
39.29
39.08
38.87
33.83
14.01
Br
N
N
S
O
O
CH3
f1 (ppm)

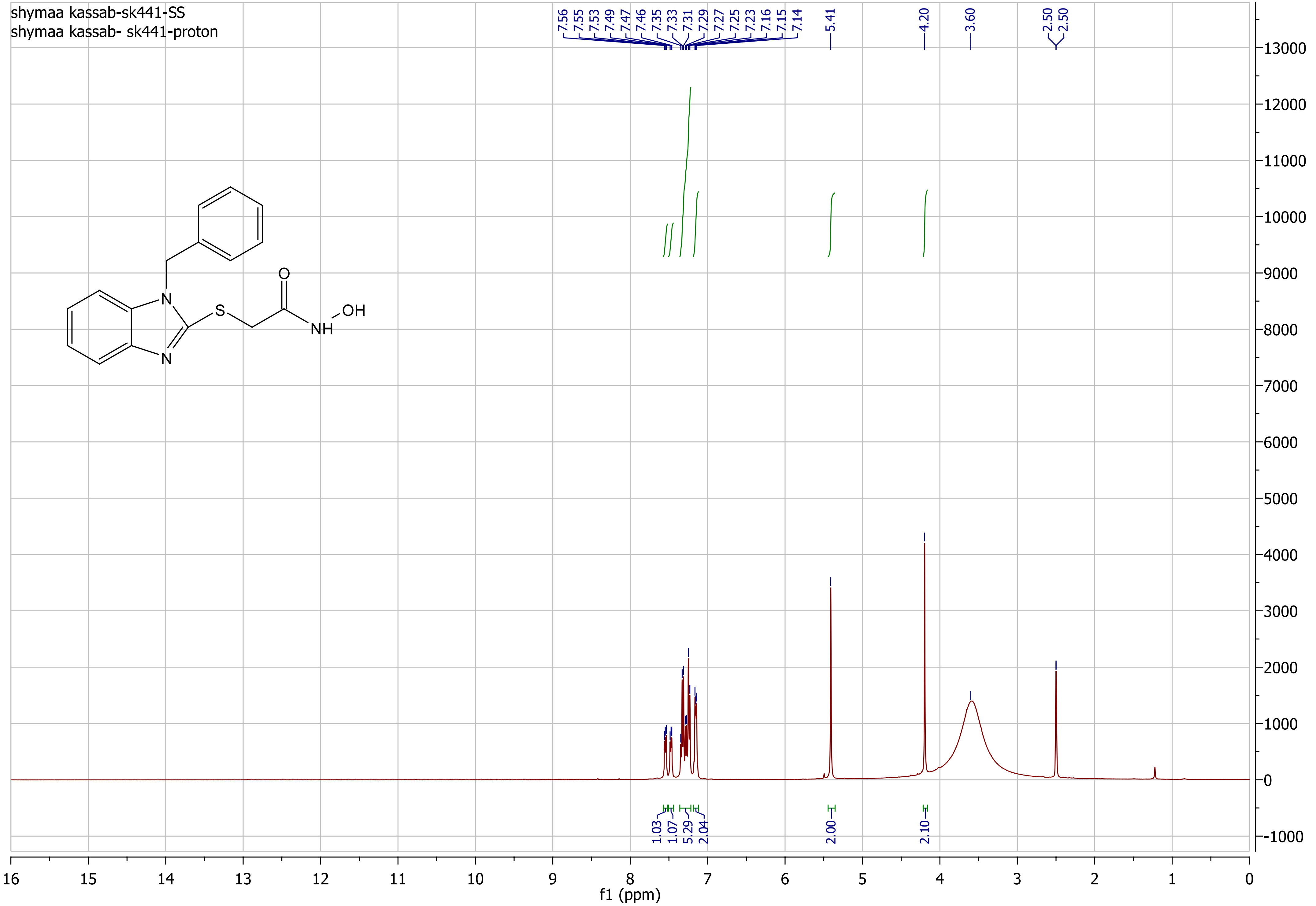
shymaa kassab-sk441-SS
shymaa kassab- sk441-proton
7.56
7.55
7.53
7.49
7.47
7.46
7.35
7.33
7.31
7.29
7.27
7.25
7.23
7.16
7.15
7.14
5.41
4.20
3.60
2.50
2.50
O
N
S
OH
NH
N
1.03
1.07
5.29
2.04
2.00
2.10
f1 (ppm)

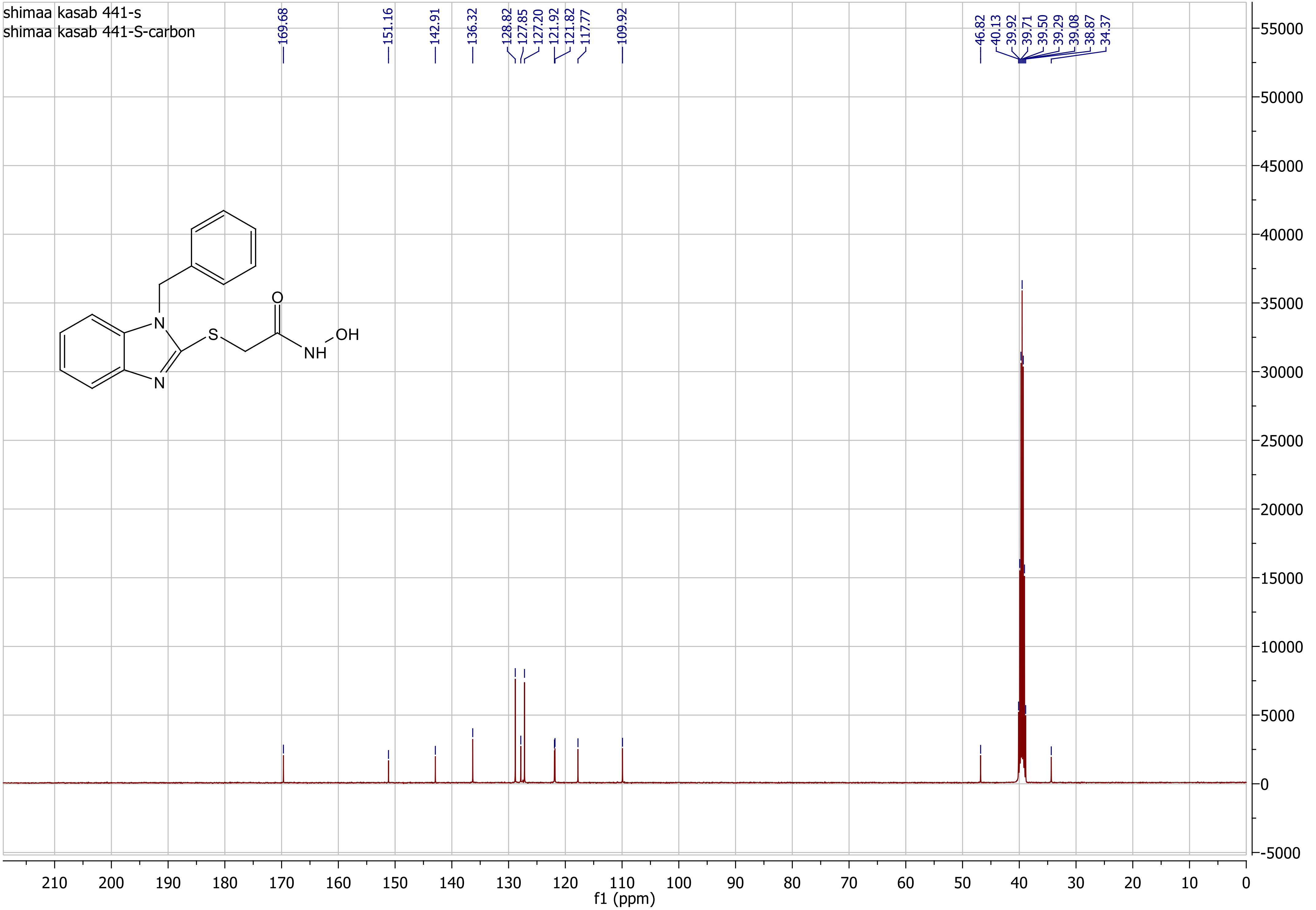
shimaa kasab 441-s
shimaa kasab 441-S-carbon
169.68
151.16
142.91
136.32
128.82
127.85
127.20
121.92
121.82
117.77
109.92
46.82
40.13
39.92
39.71
39.50
39.29
39.08
38.87
34.37
O
N
S
OH
NH
N
55000
50000
45000
40000
35000
30000
25000
20000
15000
10000
5000
0
-5000
210 200 190 180 170 160 150 140 130 120 110 100 90 80 70 60 50 40 30 20 10 0
f1 (ppm)

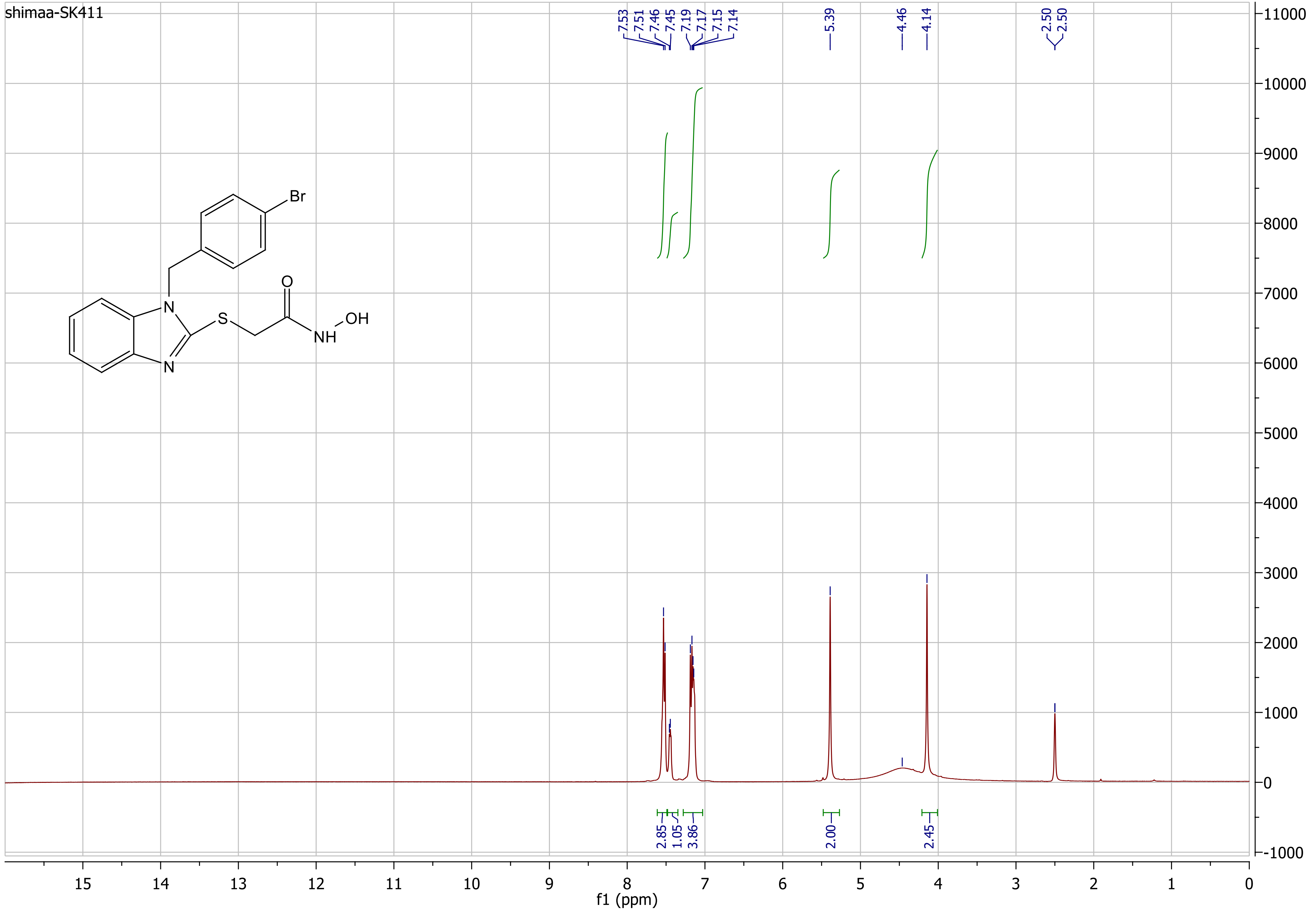
shimaa-SK411
7.53
7.51
7.46
7.45
7.19
7.17
7.15
7.14
5.39
4.46
4.14
2.50
2.50
Br
O
N
S
OH
NH
N
2.85
1.05
3.86
2.00
2.45
f1 (ppm)

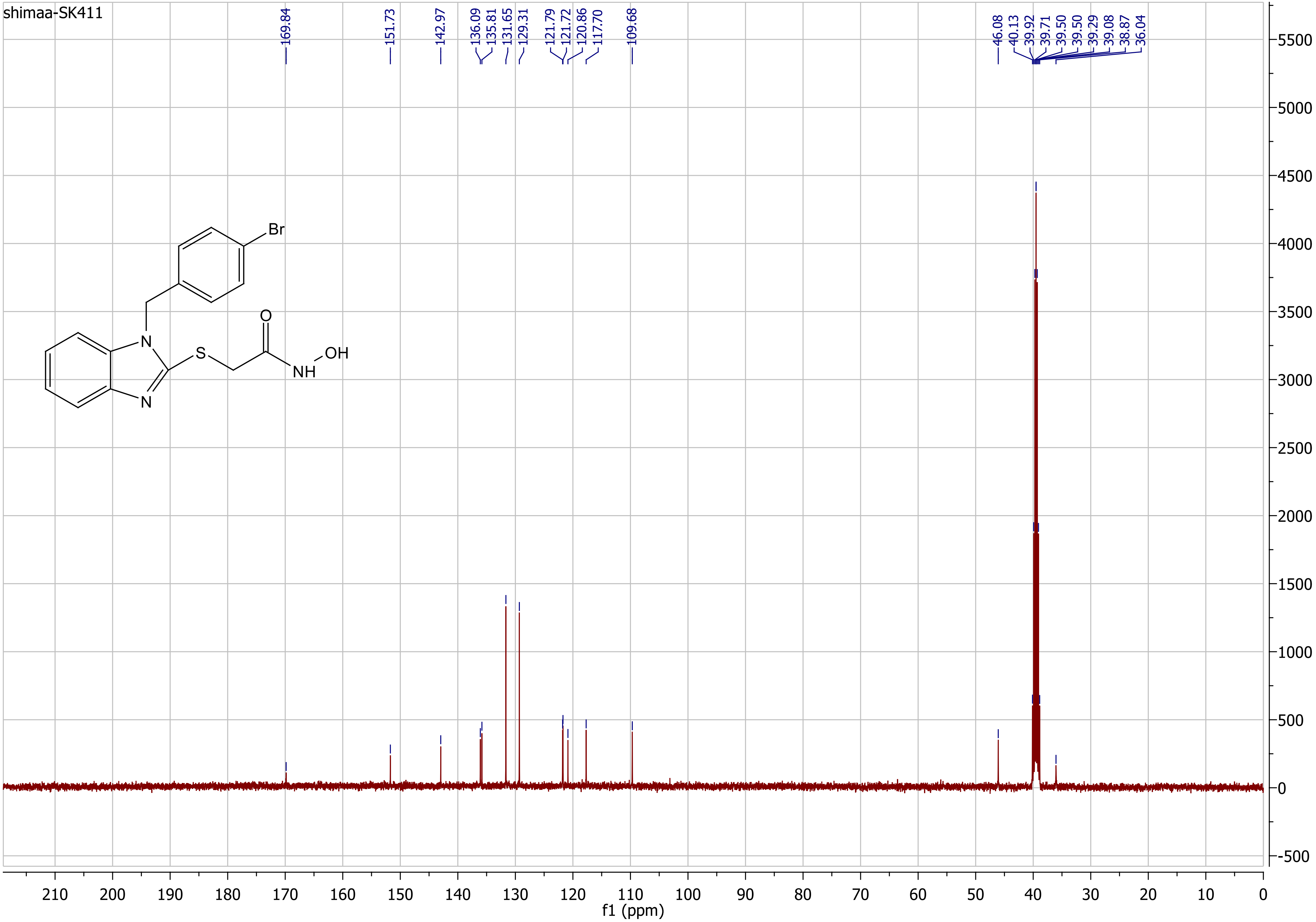

shimaa-SK411
169.84
151.73
142.97
136.09
135.81
131.65
129.31
121.79
121.72
120.86
117.70
109.68
46.08
40.13
39.92
39.71
39.50
39.50
39.29
39.08
38.87
36.04
Br
O
N
S
OH
NH
N
f1 (ppm)

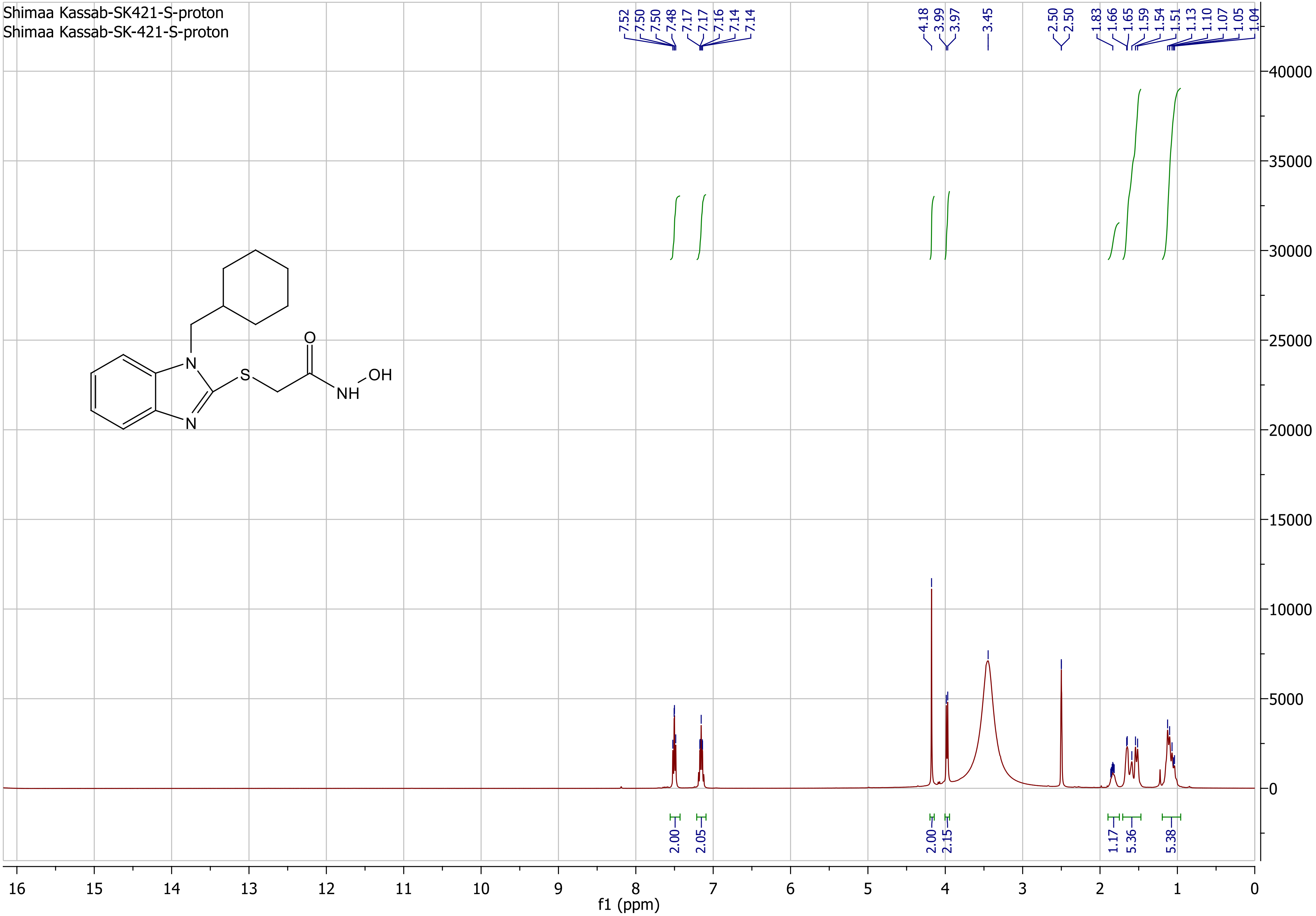
Shimaa Kassab-SK421-S-proton
Shimaa Kassab-SK-421-S-proton
7.52 7.50 7.50 7.48 7.17 7.17 7.16 7.14 7.14
4.18 3.99 3.97
3.45
2.50 2.50
1.83 1.66 1.65 1.59 1.54 1.51 1.13 1.10 1.07 1.05 1.04
O
S
N
N
NH
OH
2.00
2.05
2.00
2.15
1.17
5.36
5.38
40000
35000
30000
25000
20000
15000
10000
5000
0
16 15 14 13 12 11 10 9 8 7 6 5 4 3 2 1 0
f1 (ppm)

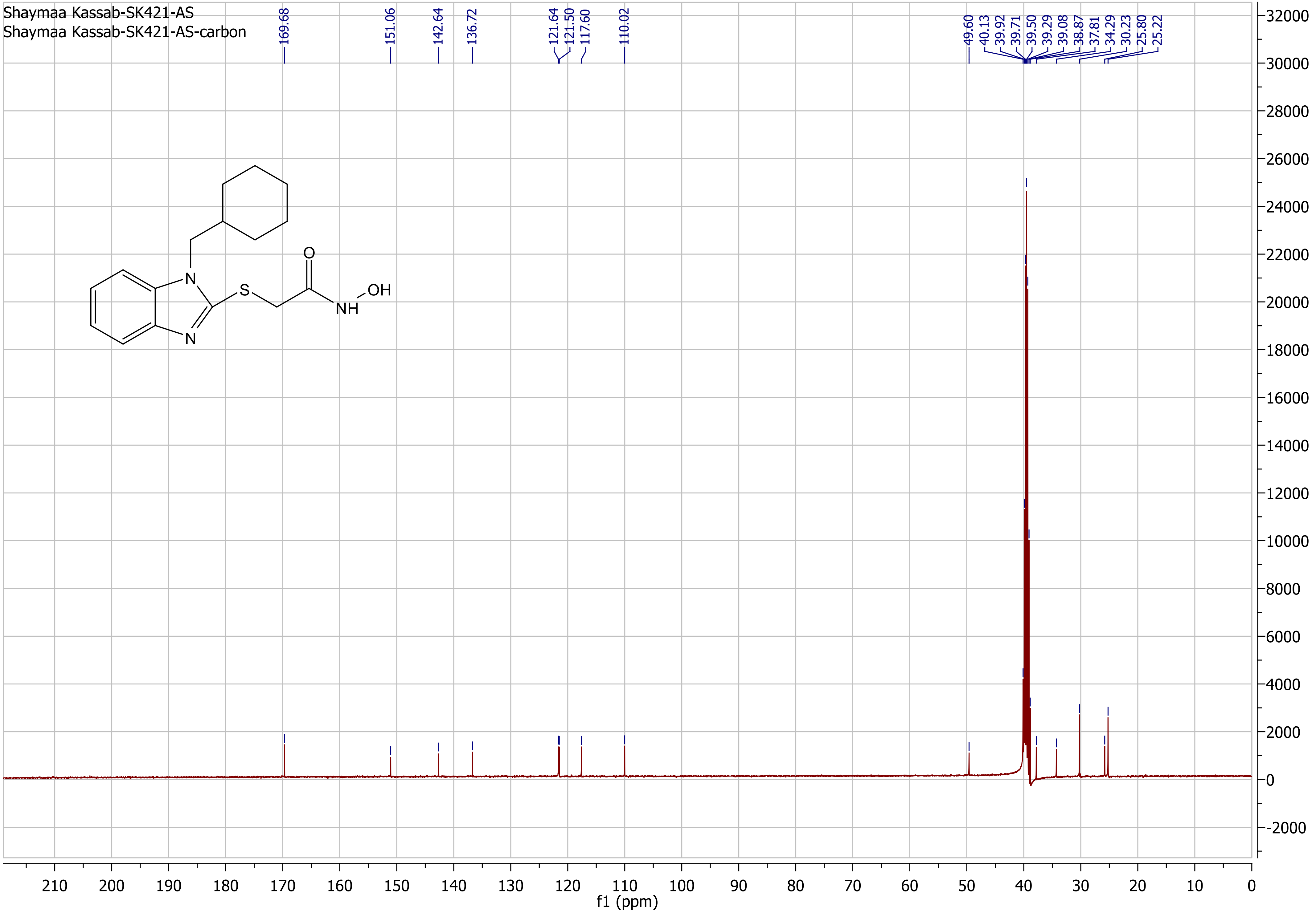
Shaymaa Kassab-SK421-AS
Shaymaa Kassab-SK421-AS-carbon
169.68
151.06
142.64
136.72
121.64
121.50
117.60
110.02
49.60
40.13
39.92
39.71
39.50
39.29
39.08
38.87
37.81
34.29
30.23
25.80
25.22
O
S
N
N
NH
OH
f1 (ppm)

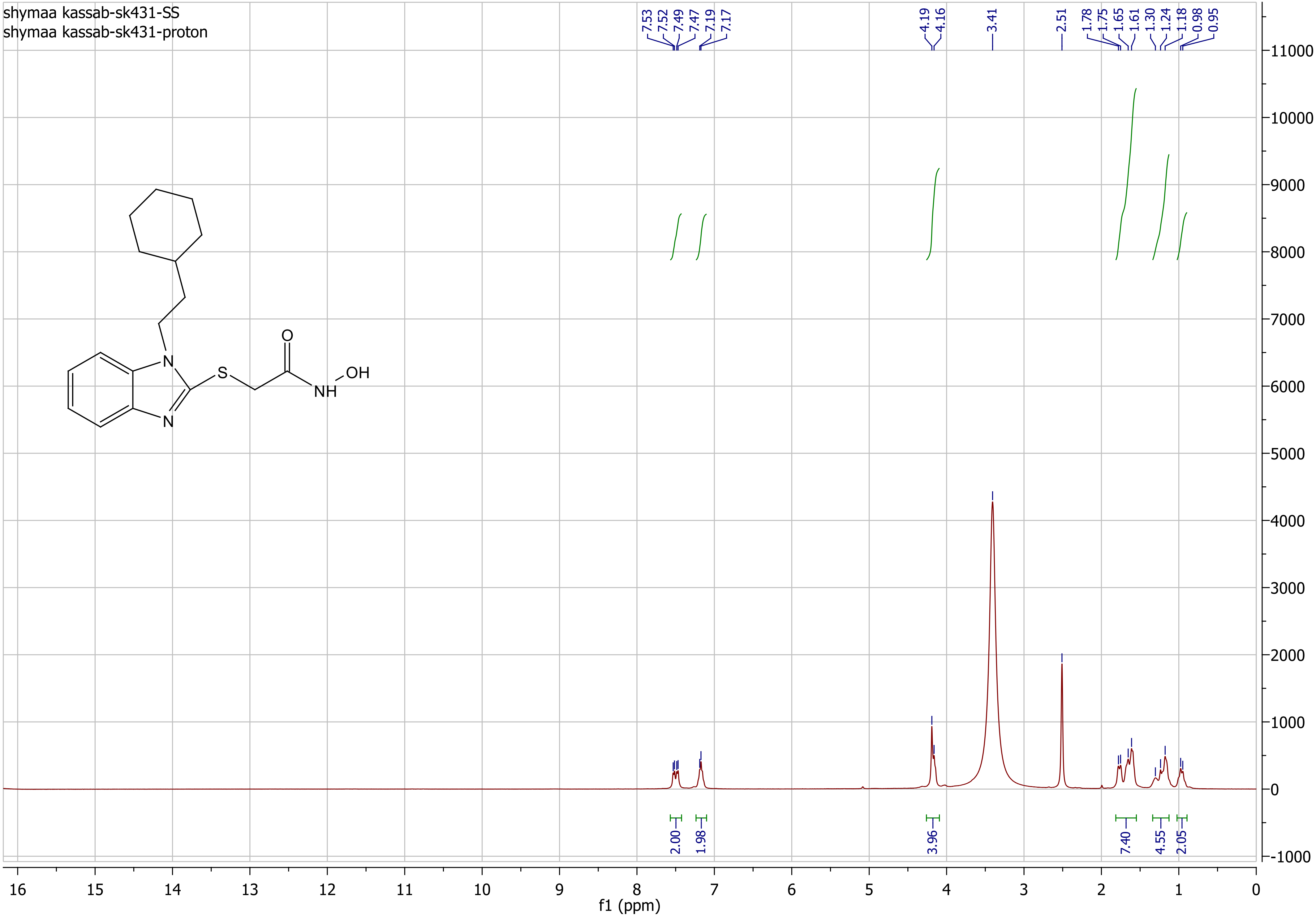
shymaa kassab-sk431-SS
shymaa kassab-sk431-proton
7.53
7.52
7.49
7.47
7.19
7.17
4.19
4.16
3.41
2.51
1.78
1.75
1.65
1.61
1.30
1.24
1.18
0.98
0.95
O
S
N
N
NH
OH
2.00
1.98
3.96
7.40
4.55
2.05
11000
10000
9000
8000
7000
6000
5000
4000
3000
2000
1000
0
-1000
16
15
14
13
12
11
10
9
8
7
6
5
4
3
2
1
0
f1 (ppm)

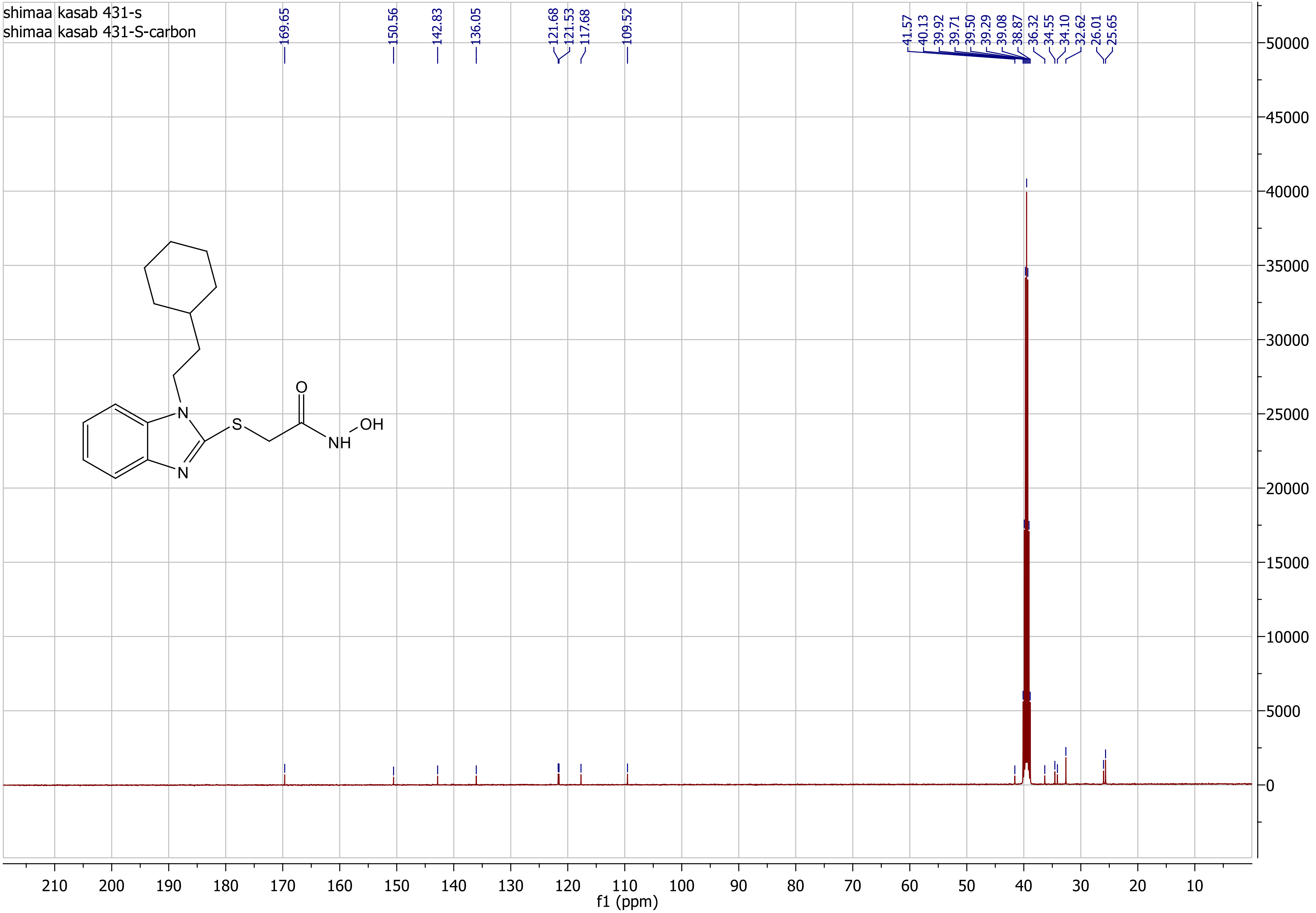

shimaa kasab 431-s
shimaa kasab 431-S-carbon
169.65
150.56
142.83
136.05
121.68
121.53
117.68
109.52
41.57
40.13
39.92
39.71
39.50
39.29
39.08
38.87
36.32
34.55
34.10
32.62
26.01
25.65
O
N
S
NH
OH
N
f1 (ppm)

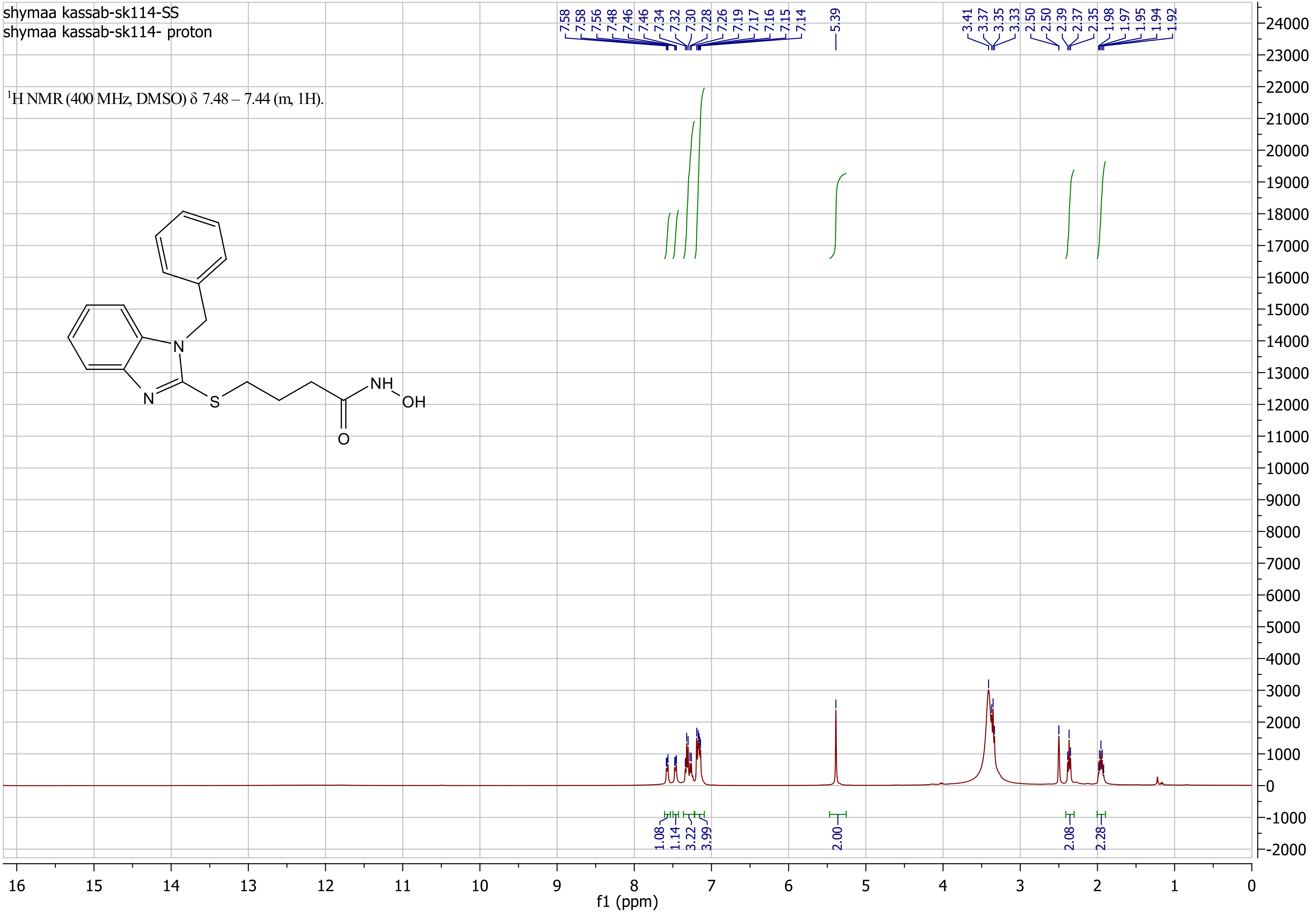
shymaa kassab-sk114-SS
shymaa kassab-sk114- proton
1H NMR (400 MHz, DMSO) δ 7.48 – 7.44 (m, 1H).
7.58 7.58 7.56 7.48 7.46 7.46 7.34 7.32 7.30 7.28 7.26 7.19 7.17 7.16 7.15 7.14
5.39
3.41 3.37 3.35 3.33 2.50 2.50 2.39 2.37 2.35 1.98 1.97 1.95 1.94 1.92
N
N
S
NH
OH
O
1.08 1.14 3.22 3.99 2.00 2.08 2.28
f1 (ppm)

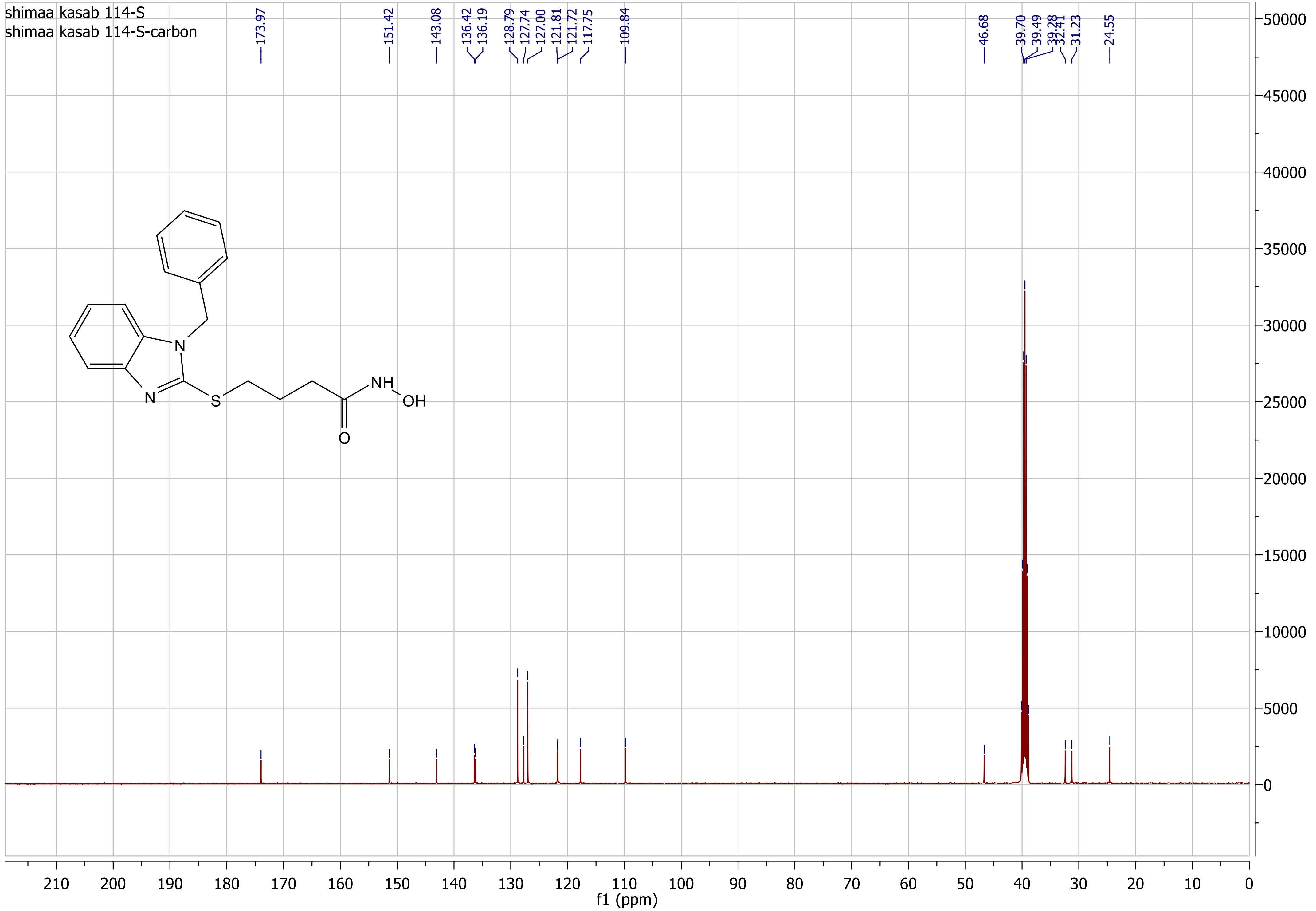
shimaa kasab 114-S
shimaa kasab 114-S-carbon
173.97
151.42
143.08
136.42
136.19
128.79
127.74
127.00
121.81
121.72
117.75
109.84
46.68
39.70
39.49
39.28
32.41
31.23
24.55
N
N
S
NH
OH
O
f1 (ppm)

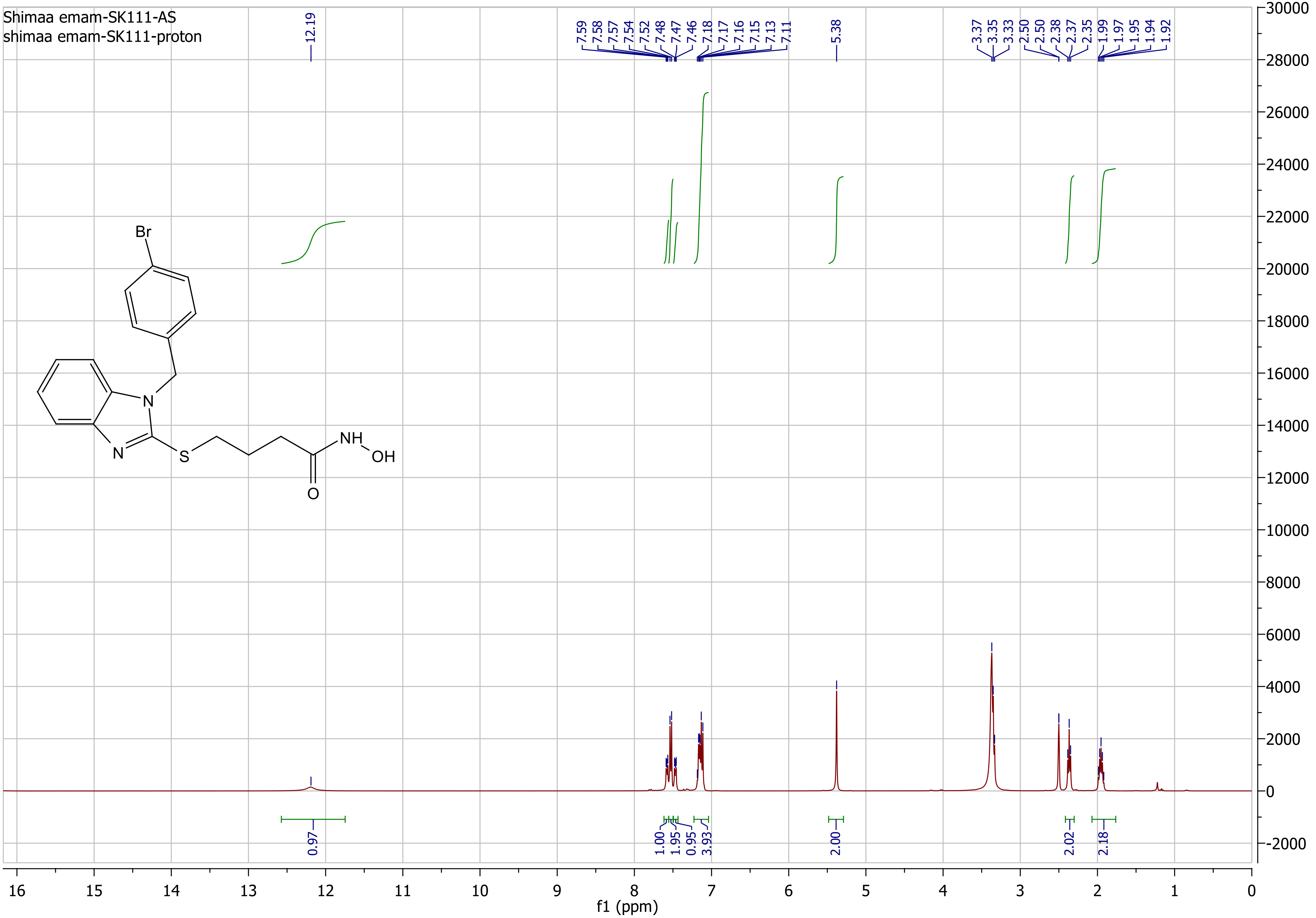
Shimaa emam-SK111-AS
shimaa emam-SK111-proton
12.19
7.59 7.58 7.57 7.54 7.52 7.48 7.47 7.46 7.18 7.17 7.16 7.15 7.13 7.11
5.38
3.37 3.35 3.33 2.50 2.50 2.38 2.37 2.35 1.99 1.97 1.95 1.94 1.92
Br
N
N
S
NH
OH
O
0.97
1.00
1.95
0.95
3.93
2.00
2.02
2.18
f1 (ppm)
16 15 14 13 12 11 10 9 8 7 6 5 4 3 2 1 0
30000 28000 26000 24000 22000 20000 18000 16000 14000 12000 10000 8000 6000 4000 2000 0 -2000

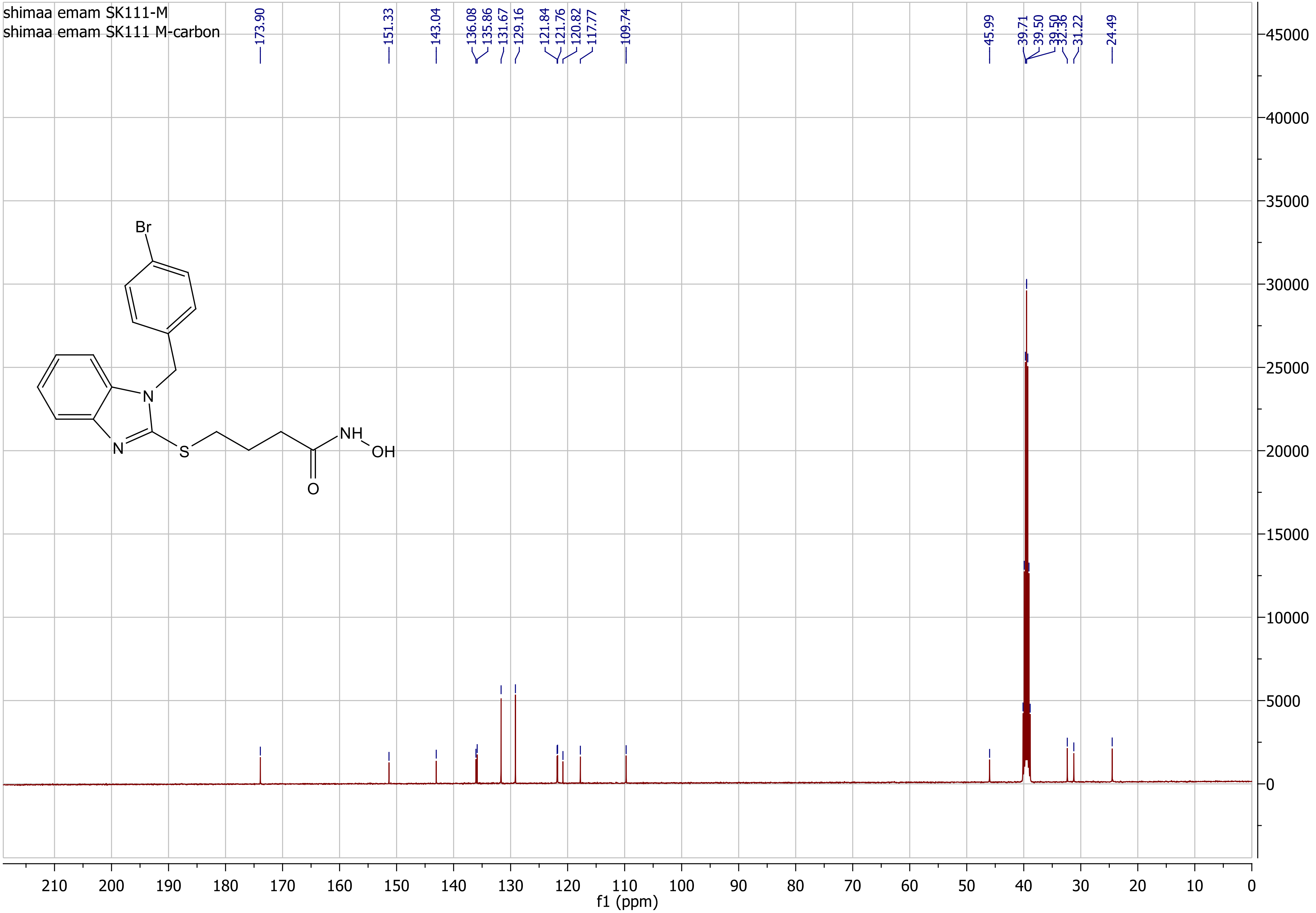
shimaa emam SK111-M
shimaa emam SK111 M-carbon
173.90
151.33
143.04
136.08
135.86
131.67
129.16
121.84
121.76
120.82
117.77
109.74
45.99
39.71
39.50
39.50
32.36
31.22
24.49
Br
N
N
S
NH
OH
O
f1 (ppm)

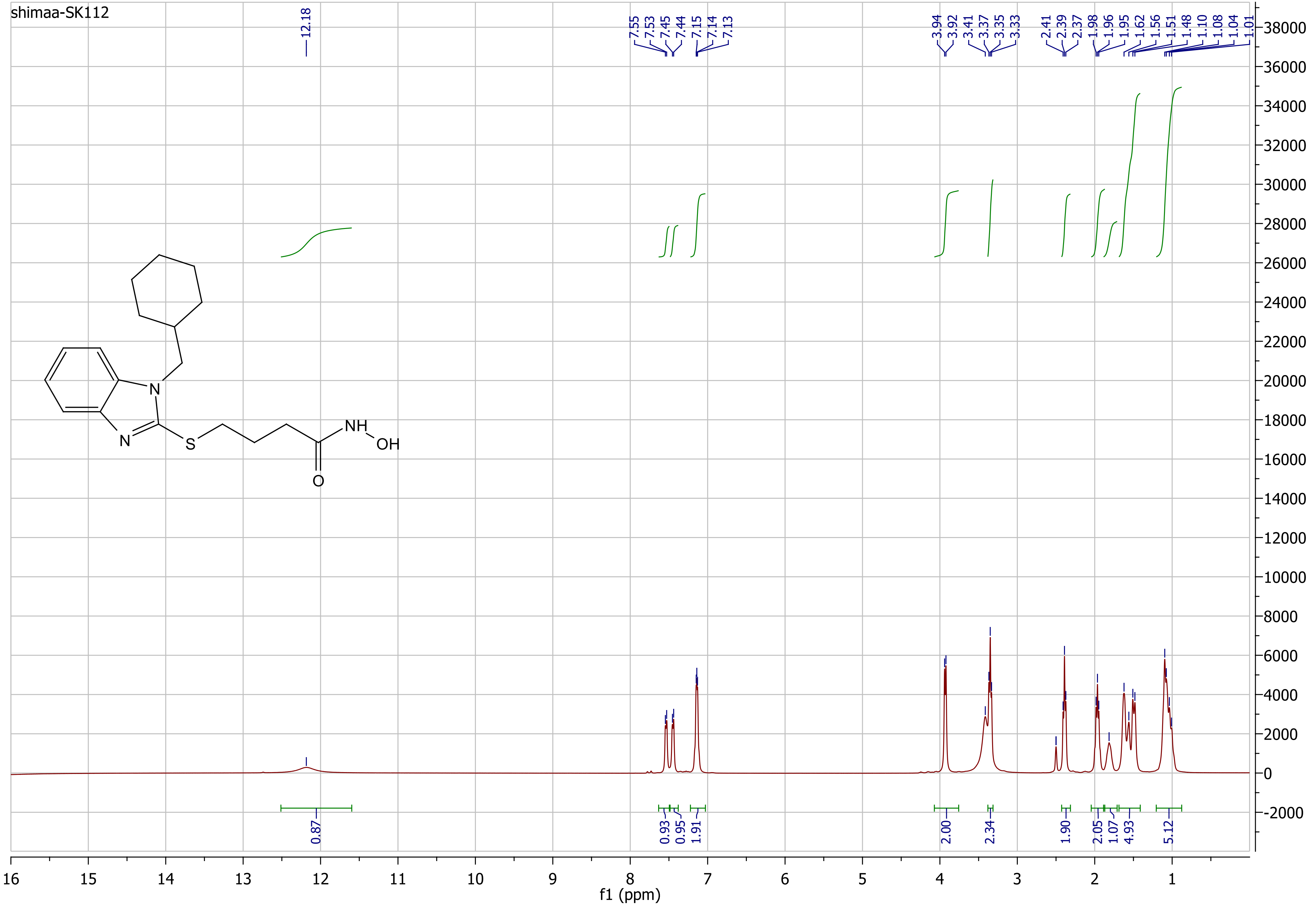
shimaa-SK112
12.18
7.55
7.53
7.45
7.44
7.15
7.14
7.13
3.94
3.92
3.41
3.37
3.35
3.33
2.41
2.39
2.37
1.98
1.96
1.95
1.62
1.56
1.51
1.48
1.10
1.08
1.04
1.01
0.87
0.93
0.95
1.91
2.00
2.34
1.90
2.05
1.07
4.93
5.12
N
N
S
NH
OH
O
f1 (ppm)

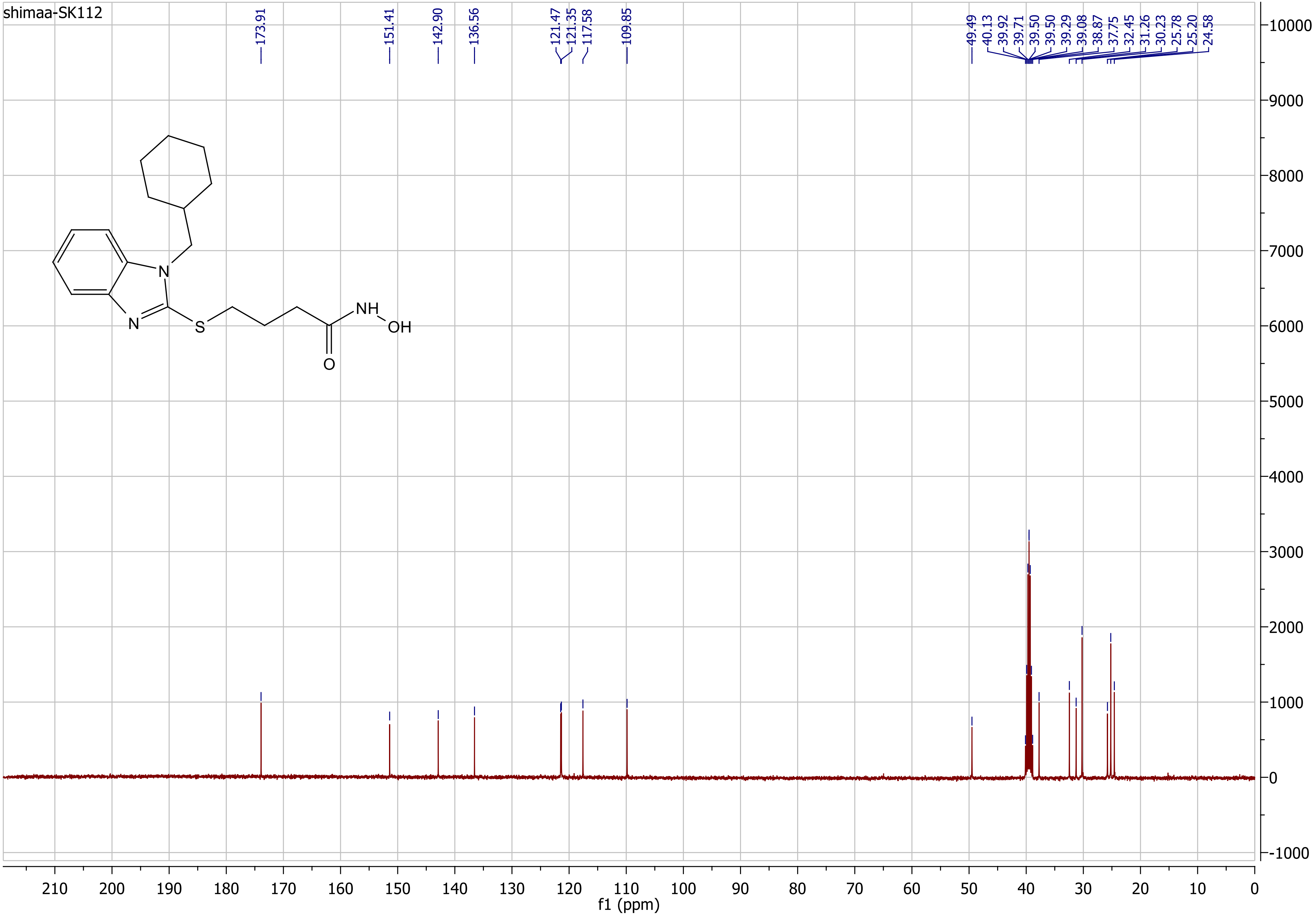

shimaa-SK112
173.91
151.41
142.90
136.56
121.47
121.35
117.58
109.85
49.49
40.13
39.92
39.71
39.50
39.50
39.29
39.08
38.87
37.75
32.45
31.26
30.23
25.78
25.20
24.58
N
N
S
NH
OH
O
f1 (ppm)

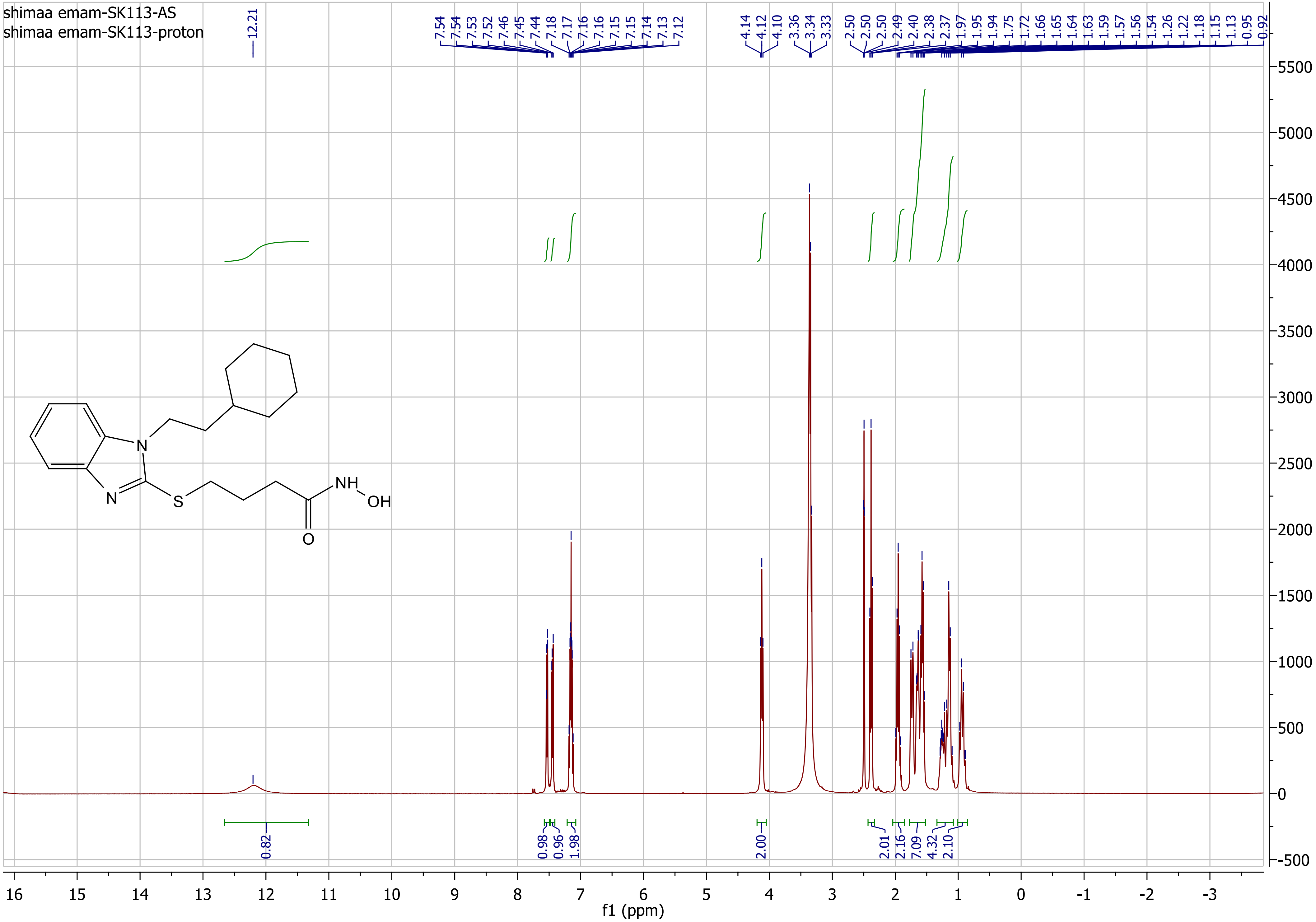

shimaa emam-SK113-AS
shimaa emam-SK113-proton
12.21
7.54
7.54
7.53
7.52
7.46
7.45
7.44
7.18
7.17
7.16
7.16
7.15
7.15
7.14
7.13
7.12
4.14
4.12
4.10
3.36
3.34
3.33
2.50
2.50
2.50
2.49
2.40
2.38
2.37
1.97
1.95
1.94
1.75
1.72
1.66
1.65
1.64
1.63
1.59
1.57
1.56
1.54
1.26
1.22
1.18
1.15
1.13
0.95
0.92
N
N
S
NH
OH
O
0.82
0.98
0.96
1.98
2.00
2.01
2.16
7.09
4.32
2.10
f1 (ppm)

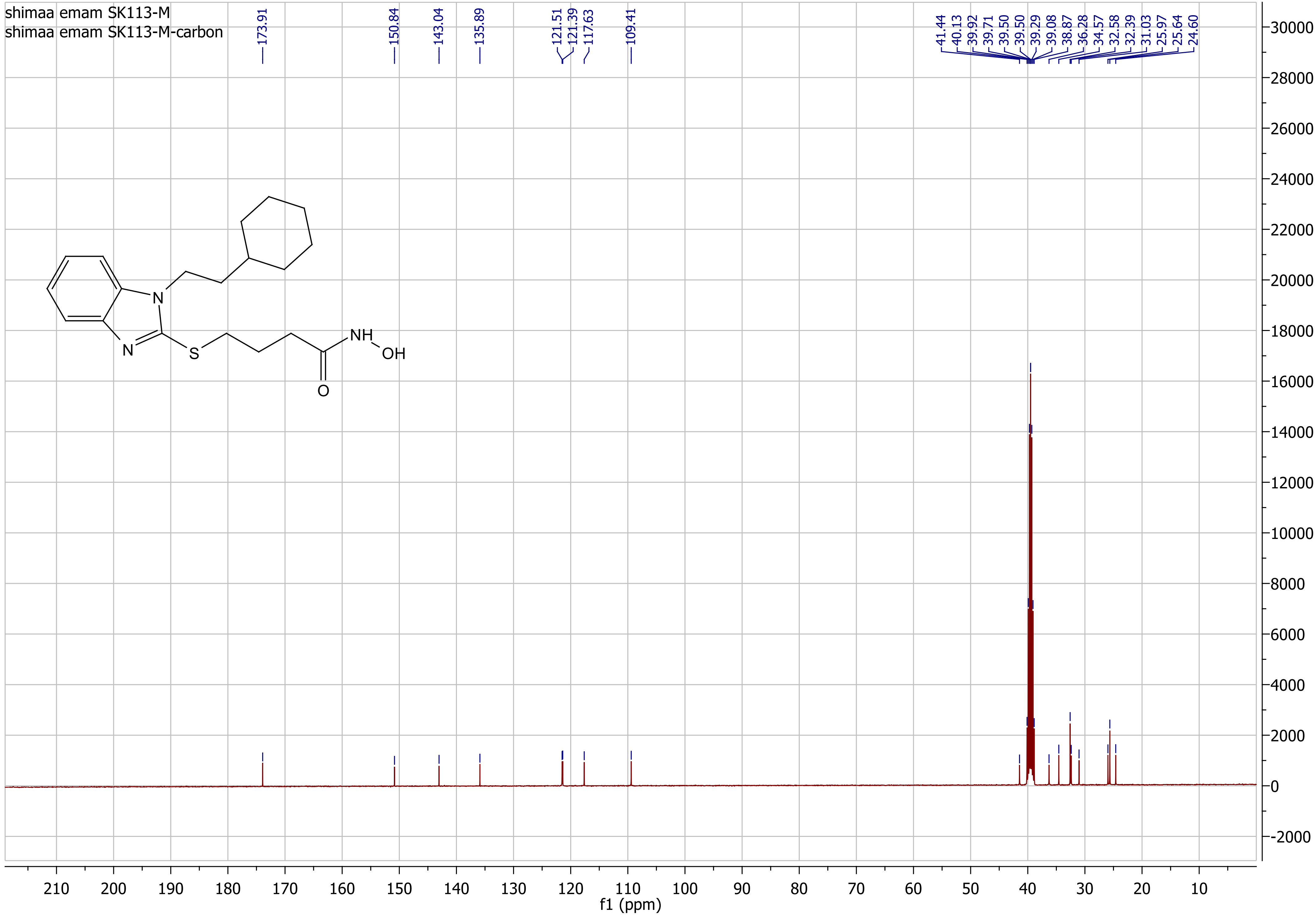
shimaa emam SK113-M
shimaa emam SK113-M-carbon
173.91
150.84
143.04
135.89
121.51
121.39
117.63
109.41
41.44
40.13
39.92
39.71
39.50
39.50
39.29
39.08
38.87
36.28
34.57
32.58
32.39
31.03
25.97
25.64
24.60
N
N
S
NH
OH
O
f1 (ppm)

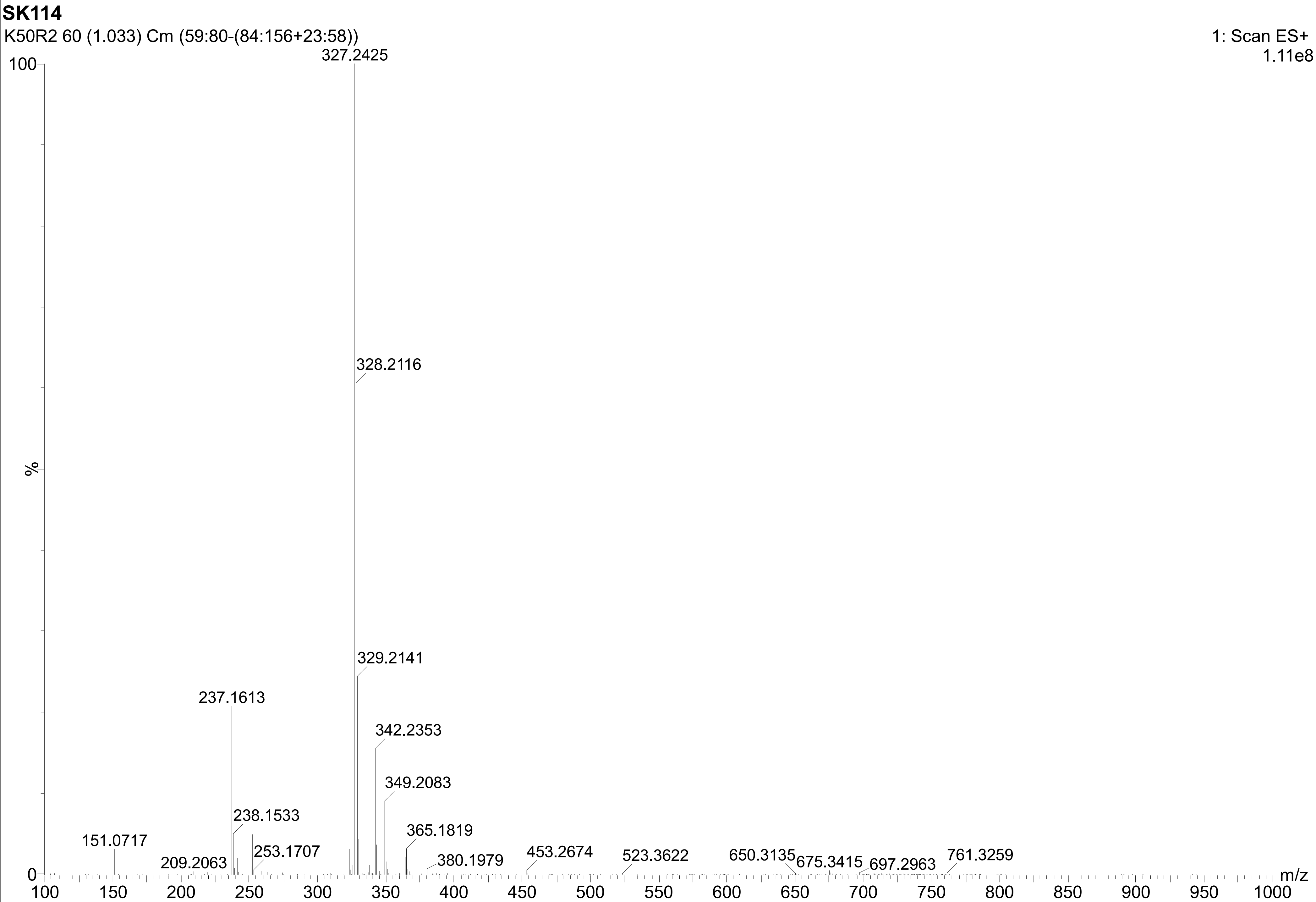

SK114
K50R2 60 (1.033) Cm (59:80-(84:156+23:58))
1: Scan ES+
1.11e8
327.2425
328.2116
329.2141
237.1613
342.2353
349.2083
238.1533
365.1819
151.0717
253.1707
209.2063
380.1979
453.2674
523.3622
650.3135
675.3415
697.2963
761.3259
%
m/z
100
0
100
150
200
250
300
350
400
450
500
550
600
650
700
750
800
850
900
950
1000

6042 Cornerstone Court West, Suite B
San Diego, CA 92121
Tel: 1.858.829.3082
Fax: 1.858.481.8694
Email: info@bpsbioscience.com

Assay Report

Histone Deacetylases (HDAC2 and HDAC6) Inhibitor Assays

Enzymatic Study of One Compound from Damanhour University

6042 Cornerstone Court West, Suite B
San Diego, CA 92121
Tel: 1.858.829.3082
Fax: 1.858.481.8694
Email: info@bpsbioscience.com

Damanhour Univ._ HDAC2 and HDAC6_20180607

HDACs Inhibitor Assays

Study Sponsor:	Damanhour University
Attention:	Dr. Shaymaa Kassab
Address:	425 Nargess Buildings, North Teseen St., New Cairo City, 11835Street, Cairo, Egypt
Study Director:	Henry Zhu, Ph.D.
Testing Facility:	BPS Bioscience Inc.<br>6042 Cornerstone Court West, Ste. B<br>San Diego, CA 92121<br>USA
Study Period:	
Report Version:	1
Report Date:	June 07th, 2018

6042 Cornerstone Court West, Suite B
San Diego, CA 92121
Tel: 1.858.829.3082
Fax: 1.858.481.8694
Email: info@bpsbioscience.com

Study Director

Cinzia Ambrosi
Scientist II

06/07/2018
Date

Henry Zhu, Ph.D.
President

06/07/2018
Date

6042 Cornerstone Court West, Suite B
San Diego, CA 92121
Tel: 1.858.829.3082
Fax: 1.858.481.8694
Email: info@bpsbioscience.com

CONTENTS

1. PURPOSE OF THE STUDY 5

2. MATERIALS AND METHODS 6

2.1. MATERIALS 6
2.2. COMPOUNDS 6
2.3. EXPERIMENTAL CONDITIONS 6
2.3.1. Enzymes and Substrates ***6***
2.3.2. Assay Conditions ***6***
2.3.3. Data Analysis ***7***

3. ASSAY RESULTS 8

3.1. SUMMARY OF THE INHIBITORY EFFECTS OF ONE COMPOUND ON INDIVIDUAL HDAC ACTIVITIES 8
3.2. RESULTS OF THE EFFECTS OF THE COMPOUNDS ON INDIVIDUAL HDAC ACTIVITY 9
3.2.1. HDAC ***9***
3.2.1.1. HDAC2 ..... ***9***
3.2.1.2. HDAC6 ..... ***10***

4. QUALITY ASSURANCE STATEMENT 11

6042 Cornerstone Court West, Suite B
San Diego, CA 92121
Tel: 1.858.829.3082
Fax: 1.858.481.8694
Email: info@bpsbioscience.com

1. Purpose of the Study

The purpose of the study is to determine the effects of eight compounds from Damanhour University on the enzymatic activities of recombinant human HDAC2 and HDAC6 using an *in vitro* enzymatic assay.

6042 Cornerstone Court West, Suite B
San Diego, CA 92121
Tel: 1.858.829.3082
Fax: 1.858.481.8694
Email: info@bpsbioscience.com

2. Materials and Methods

2.1. Materials

SAHA is purchased from Cayman Chemicals (Ann Arbor, MI, Catalog Number 10009929).
HDAC Assay Buffer (BPS catalog number 50031).
HDAC Assay Developer (BPS catalog number 50030).
HDAC Substrate 3 (BPS catalog number 50037).

2.2. Compounds

The test compound is supplied by Damanhour University.

SK 114	Powder	10 mM	DMSO	10	10 % DMSO in HDAC Assay Buffer
*SAHA	Powder	10 mM	DMSO	0.03-0.3-3	10 % DMSO in HDAC Assay Buffer

*Reference compound.

2.3. Experimental Conditions

2.3.1. Enzymes and Substrates

Assay	Catalog #	Enzyme Lot #	Enzyme Used (ng) / Reaction	Substrate
HDAC2	50002	151125-G	7.5	10 µM HDAC Substrate 3 50037
HDAC6	50006	170520	10	10 µM HDAC Substrate 3 50037

2.3.2. Assay Conditions

The compounds were dissolved in DMSO. A series of dilutions of the compounds was prepared with 10% DMSO in HDAC assay buffer and 5µl of the dilution was added to a 50µl reaction so that the final concentration of DMSO is 1% in all of the reactions.

6042 Cornerstone Court West, Suite B
San Diego, CA 92121
Tel: 1.858.829.3082
Fax: 1.858.481.8694
Email: info@bpsbioscience.com

The enzymatic reactions for the HDAC enzymes were conducted in duplicate at 37ºC for 30 minutes in a 50µl mixture containing HDAC assay buffer, 5µg BSA, an HDAC substrate (see 2.3.1), a HDAC enzyme (see 2.3.1) and a test compound (see 2.2).

After enzymatic reactions, 50µl of 2 x HDAC Developer was added to each well and the plate was incubated at room temperature for an additional 15 minutes.

Fluorescence intensity was measured at an excitation of 360 nm and an emission of 460 nm using a Tecan Infinite M1000 microplate reader.

2.3.3. Data Analysis

HDAC activity assays were performed in duplicate. The fluorescent intensity data were analyzed using the computer software, GraphPad Prism. In the absence of the compound, the fluorescent intensity (F_t) in each data set was defined as 100% activity. In the absence of HDAC, the fluorescent intensity (F_b) in each data set was defined as 0% activity. The percent activity in the presence of each compound was calculated according to the following equation: %activity = $(F-F_b)/(F_t-F_b)$, where F= the fluorescent intensity in the presence of the compound.

The values of percentage activity were plotted on a bar graph.

6042 Cornerstone Court West, Suite B
San Diego, CA 92121
Tel: 1.858.829.3082
Fax: 1.858.481.8694
Email: info@bpsbioscience.com

3. Assay Results

3.1. Summary of the Inhibitory Effects of One Compound on Individual HDAC Activities

The percentage inhibition of the compounds against HDAC enzymes is summarized in Table 3.1.

Table 3.1. Inhibitory Effects of the Compound on HDAC Activities

Compounds	Conc. (μM)	% Inhibition	
		HDAC2	HDAC6
SK 114	10	19	92
SAHA	0.03	70	67
	0.3	96	82
	3	100	100

6042 Cornerstone Court West, Suite B
San Diego, CA 92121
Tel: 1.858.829.3082
Fax: 1.858.481.8694
Email: info@bpsbioscience.com

3.2. Results of the Effects of the Compound on Individual HDAC Activities

3.2.1. HDAC

3.2.1.1. HDAC2

Table 3.2.1.1. Data for the Effect of One Compound on HDAC2 Activity

Compounds	Conc. μM	HDAC2 Activity (Fluorescence count)		% Activity		% Inhibition
		Repeat1	Repeat2	Repeat1	Repeat2	
No Compound		7777	7719	100	100	0
SK 114	10	6362	6375	81	81	19
SAHA	0.03	2790	2771	30	30	70
	0.3	913	937	4	4	96
	3	593	609	0	0	100
Background		630	639			

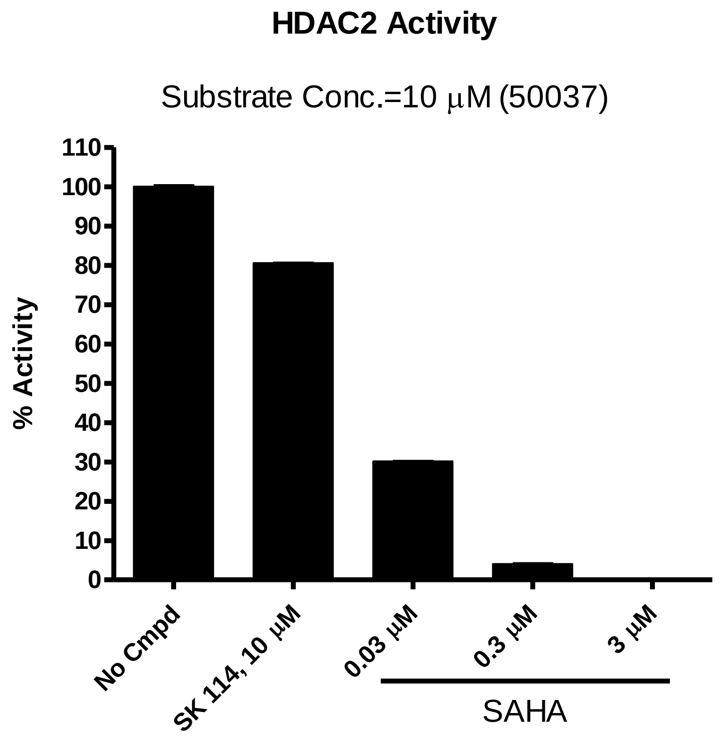

BPS Bioscience

6042 Cornerstone Court West, Suite B
San Diego, CA 92121
Tel: 1.858.829.3082
Fax: 1.858.481.8694
Email: info@bpsbioscience.com

3.2.1.2. HDAC6

Table 3.2.1.2. Data for the Effect of One Compound on HDAC6 Activity

Compounds	Conc. µM	HDAC6 Activity (Fluorescence count)		% Activity		% Inhibition
		Repeat1	Repeat2	Repeat1	Repeat2	
No Compound		5425	5468	100	100	0
SK 114	10	1043	1040	8	8	92
SAHA	0.03	2191	2298	32	35	67
	0.3	1518	1523	18	18	82
	3	611	611	0	0	100
Background		630	639			

HDAC6 Activity

Substrate Conc.=10 µM (50037)

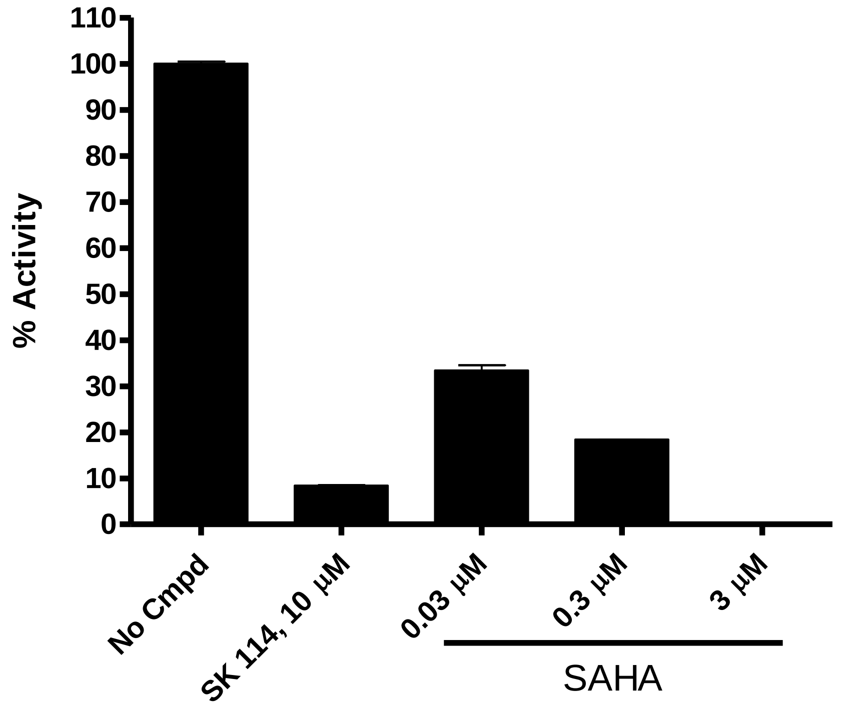

6042 Cornerstone Court West, Suite B
San Diego, CA 92121
Tel: 1.858.829.3082
Fax: 1.858.481.8694
Email: info@bpsbioscience.com

4. Quality Assurance Statement

I certify that the results presented in this report were generated using the materials and methods mentioned and that these results reflect the Raw Data.

Henry Zhu, Ph.D.
President

06/07/2018
Date

6042 Cornerstone Court West, Suite B
San Diego, CA 92121
Tel: 1.858.829.3082
Fax: 1.858.481.8694
Email: info@bpsbioscience.com

Assay Report

Histone Deacetylase (HDAC) Inhibitor Assays

Enzymatic Study of One Compound from Damanhour University

6042 Cornerstone Court West, Suite B
San Diego, CA 92121
Tel: 1.858.829.3082
Fax: 1.858.481.8694
Email: info@bpsbioscience.com

DHU_HDAC_181016

HDAC Inhibitor Assays

Study Sponsor:	Damanhour University
Attention:	Dr. Shaymaa Kassab
Address:	Damanhour University<br>425 Nargess Buildings<br>North Teseen St.<br>New Cairo City<br>Cairo, Egypt 11835
Study Director:	Henry Zhu, Ph.D.
Testing Facility:	BPS Bioscience Inc.<br>6042 Cornerstone Court West, Ste. B<br>San Diego, CA 92121<br>USA
Study Period:	
Report Version:	1
Report Date:	October 16, 2018

6042 Cornerstone Court West, Suite B
San Diego, CA 92121
Tel: 1.858.829.3082
Fax: 1.858.481.8694
Email: info@bpsbioscience.com

Study Director

Kevin Kurtz
Sr. Scientist II.

10-16-18

Date

Henry Zhu, Ph.D.
President

10-16-18

Date

6042 Cornerstone Court West, Suite B
San Diego, CA 92121
Tel: 1.858.829.3082
Fax: 1.858.481.8694
Email: info@bpsbioscience.com

CONTENTS

HISTONE DEACETYLASE (HDAC) INHIBITOR ASSAYS....1

ENZYMATIC STUDY OF ONE COMPOUND FROM DAMANHOUR UNIVERSITY 1

HDAC INHIBITOR ASSAYS....2

STUDY DIRECTOR....3

1. PURPOSE OF THE STUDY 5

2. MATERIALS AND METHODS 6

2.1 MATERIALS 6
2.2 COMPOUNDS....6
2.3 EXPERIMENTAL CONDITIONS 6
2.3.1 Enzymes and Substrates 6
2.3.2 Assay Conditions....7
2.3.3 Data Analysis 7

3. ASSAY RESULTS 8

3.1. SUMMARY OF THE INHIBITORY EFFECTS OF THE COMPOUND ON INDIVIDUAL HDAC ACTIVITIES 8
3.2. RESULTS OF THE EFFECTS OF THE COMPOUNDS ON INDIVIDUAL HDAC ACTIVITY 9
3.2.1. HDAC3/NCOR2....9
3.2.2. HDAC4....10
3.2.3. HDAC5....11
3.2.4. HDAC7....12
3.2.5. HDAC9....13
3.2.6. HDAC10....14
3.2.7. HDAC11....15

4. QUALITY ASSURANCE STATEMENT 16

6042 Cornerstone Court West, Suite B
San Diego, CA 92121
Tel: 1.858.829.3082
Fax: 1.858.481.8694
Email: info@bpsbioscience.com

1. Purpose of the Study

The purpose of the study is to determine the effects of one compound from Damanhour University on the enzymatic activities of recombinant human HDAC3/NCOR2, HDAC4, HDAC5, HDAC7, HDAC9, HDAC10, and HDAC11 using an in vitro enzymatic assay.

6042 Cornerstone Court West, Suite B
San Diego, CA 92121
Tel: 1.858.829.3082
Fax: 1.858.481.8694
Email: info@bpsbioscience.com

2. Materials and Methods

2.1 Materials

HDAC Assay Buffer (BPS catalog number 50031)
HDAC Assay Developer (BPS catalog number 50030)
HDAC Substrate 3 (BPS number 50037)
HDAC Class 2a Substrate 1 (BPS number 50040)
SAHA is purchased from Cayman Chemicals (Ann Arbor, MI, Catalog Number 10009929).
TSA is purchased from Selleck (Houston, TX, Catalog number S1045).

2.2 Compounds

The test compounds are supplied by Damanhour University.

Compound I.D.	Compound Supplied	Stock Concentration	Dissolving Solvent	Test Range (µM)	Intermediate Dilution
SK 114	Powder	10mM	DMSO	10	10 % DMSO in HDAC Assay Buffer
SAHA*	Powder	10mM	DMSO	1, 0.1, 0.01	10 % DMSO in HDAC Assay Buffer
TSA*	Powder	10mM	DMSO	100, 10, 1	10 % DMSO in HDAC Assay Buffer

*Reference Compound

2.3 Experimental Conditions

2.3.1 Enzymes and Substrates

Assay	Catalog #	Enzyme Lot #	Enzyme Used (ng) / Reaction	Substrate
HDAC3/NCOR2	50003	130819	3.4	10µM HDAC Substrate 3
HDAC4	50004	130828-G	0.3	2µM HDAC Substrate Class 2a
HDAC5	50005	140211	44	2µM HDAC Substrate Class 2a
HDAC7	50007	150629-5	1.6	2µM HDAC Substrate Class 2a
HDAC9	50009	130502-66	4.3	2µM HDAC Substrate Class 2a
HDAC10	50010	170912	600	10µM HDAC Substrate 3
HDAC11	50011	160222	60	2µM HDAC Substrate Class 2a

2.3.2 Assay Conditions

The compounds are dissolved in DMSO. A series of dilutions of the compounds were prepared with 10% DMSO in HDAC assay buffer and 5µl of the dilution was added to a 50µl reaction so that the final concentration of DMSO is 1% in all of reactions.

The enzymatic reactions for the HDAC enzymes were conducted in duplicate at 37ºC for 30 minutes in a 50µl mixture containing HDAC assay buffer, 5µg BSA, an HDAC substrate (see 2.3.1), a HDAC enzyme (see 2.3.1) and a test compound (see 2.2).

After enzymatic reactions, 50µl of 2 x HDAC Developer was added to each well for the HDAC enzymes and the plate was incubated at room temperature for an additional 15 minutes.

Fluorescence intensity was measured at an excitation of 360 nm and an emission of 460 nm using a Tecan Infinite M1000 microplate reader.

2.3.3 Data Analysis

HDAC activity assays were performed in duplicates at each concentration. The fluorescent intensity data were analyzed using the computer software, Graphpad Prism. In the absence of the compound, the fluorescent intensity (F_t) in each data set was defined as 100% activity. In the absence of HDAC, the fluorescent intensity (F_b) in each data set was defined as 0% activity. The percent activity in the presence of each compound was calculated according to the following equation: %activity = $(F-F_b)/(F_t-F_b)$, where F= the fluorescent intensity in the presence of the compound.

The values of percentage activity were plotted on a bar graph.

6042 Cornerstone Court West, Suite B
San Diego, CA 92121
Tel: 1.858.829.3082
Fax: 1.858.481.8694
Email: info@bpsbioscience.com

3. Assay Results

3.1. Summary of the Inhibitory Effects of the Compound on Individual HDAC Activities

The percentage inhibition of the one compound against HDACs is summarized on Table 3.1.

Table 3.1 Inhibitory Effects of the Compounds on HDAC Activities

Inhibitors	% Inhibition						
	**HDAC3 /NCOR2**	**HDAC4**	**HDAC5**	**HDAC7**	**HDAC9**	**HDAC10**	**HDAC11**
SK 114	37	1	4	2	5	28	6
SAHA, 0.01μM	16					34	
SAHA, 0.1μM	64					74	
SAHA, 1μM	94					97	
TSA, 1μM		36	33	31	15		15
TSA, 10μM		64	64	78	60		49
TSA, 100μM		92	93	97	95		91

6042 Cornerstone Court West, Suite B
San Diego, CA 92121
Tel: 1.858.829.3082
Fax: 1.858.481.8694
Email: info@bpsbioscience.com

3.2. Results of the Effects of the Compounds on Individual HDAC Activity

3.2.1. HDAC3/NCOR2

Table 3.2.1. Data for the Effect of the Compounds on HDAC3/NCOR2 Activity

Compounds	HDAC Activity (Fluorescence count)		% Activity		% Inhibition
	Repeat1	Repeat2	Repeat1	Repeat2	
No Compound	26848	26221	101	99	0
SK 114	16891	17038	62	63	37
SAHA, 0.01μM	22865	21873	86	82	16
SAHA, 0.1μM	10116	10182	36	36	64
SAHA, 1μM	2251	2469	5	6	94
Background	871	854			

HDAC3/NCOR2 Activity

Substrate Conc.=10 μM Substrate 3 (50037)

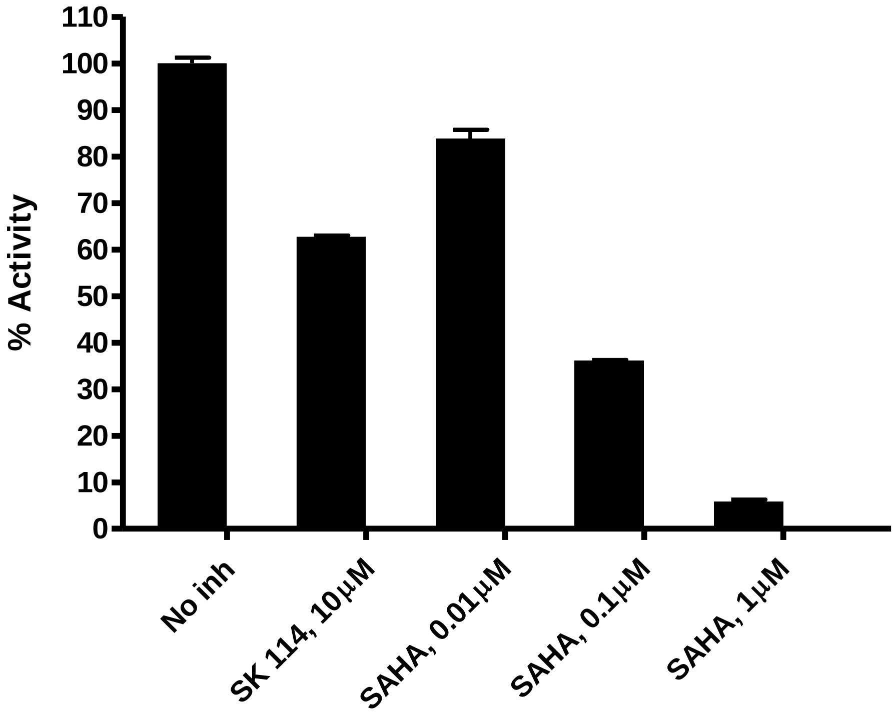

6042 Cornerstone Court West, Suite B
San Diego, CA 92121
Tel: 1.858.829.3082
Fax: 1.858.481.8694
Email: info@bpsbioscience.com

3.2.2. <u>HDAC4</u>

Table 3.2.2. Data for the Effect of the Compounds on HDAC4 Activity

Compounds	HDAC Activity (Fluorescence count)		% Activity		% Inhibition
	Repeat1	Repeat2	Repeat1	Repeat2	
No Compound	27153	27550	99	101	0
SK 114	26757	27199	98	99	1
TSA, 1μM	17735	18019	64	65	36
TSA, 10μM	10390	10000	36	35	64
TSA, 100μM	2894	2911	8	8	92
Background	748	722			

HDAC4 Activity

Substrate Conc.=2 μM Class 2a Substrate 1 (50040)

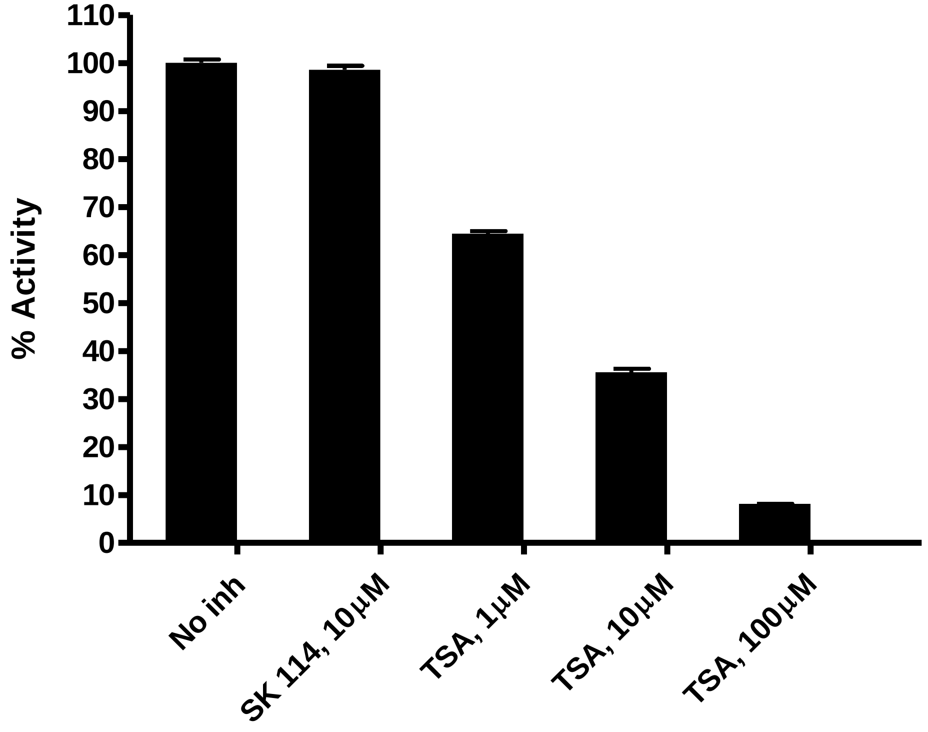

6042 Cornerstone Court West, Suite B
San Diego, CA 92121
Tel: 1.858.829.3082
Fax: 1.858.481.8694
Email: info@bpsbioscience.com

3.2.3. HDAC5

Table 3.2.3. Data for the Effect of the Compounds on HDAC5 Activity

Compounds	HDAC Activity (Fluorescence count)		% Activity		% Inhibition
	Repeat1	Repeat2	Repeat1	Repeat2	
No Compound	7217	7314	99	101	0
SK 114	7054	7002	97	96	4
TSA, 1μM	5188	5035	68	66	33
TSA, 10μM	3145	3076	37	36	64
TSA, 100μM	1156	1191	6	7	93
Background	739	734			

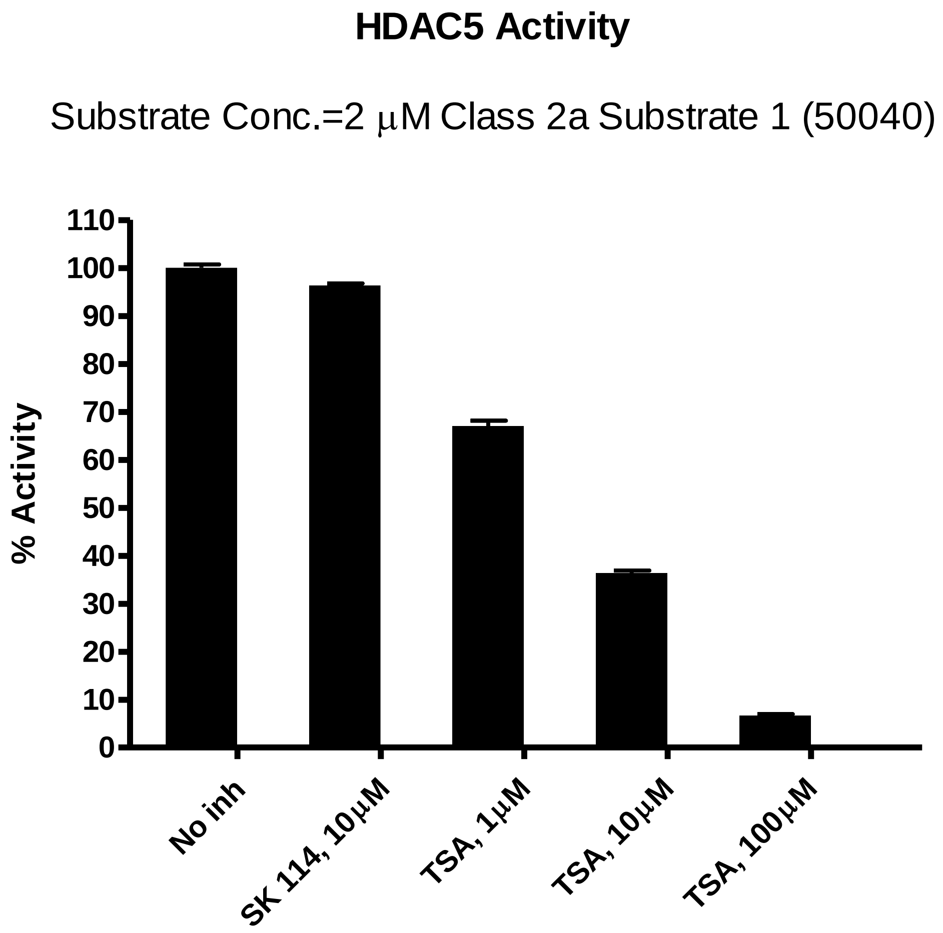

BPS Bioscience
6042 Cornerstone Court West, Suite B
San Diego, CA 92121
Tel: 1.858.829.3082
Fax: 1.858.481.8694
Email: info@bpsbioscience.com

3.2.4. HDAC7

Table 3.2.4. Data for the Effect of the Compounds on HDAC7 Activity

Compounds	HDAC Activity (Fluorescence count)		% Activity		% Inhibition
	Repeat1	Repeat2	Repeat1	Repeat2	
No Compound	26375	27435	98	102	0
SK 114	26807	25934	100	96	2
TSA, 1µM	18549	19044	68	70	31
TSA, 10µM	6346	6725	21	23	78
TSA, 100µM	1625	1690	3	4	97
Background	740	757			

HDAC7 Activity

Substrate Conc.=2 µM Class 2a Substrate 1 (50040)

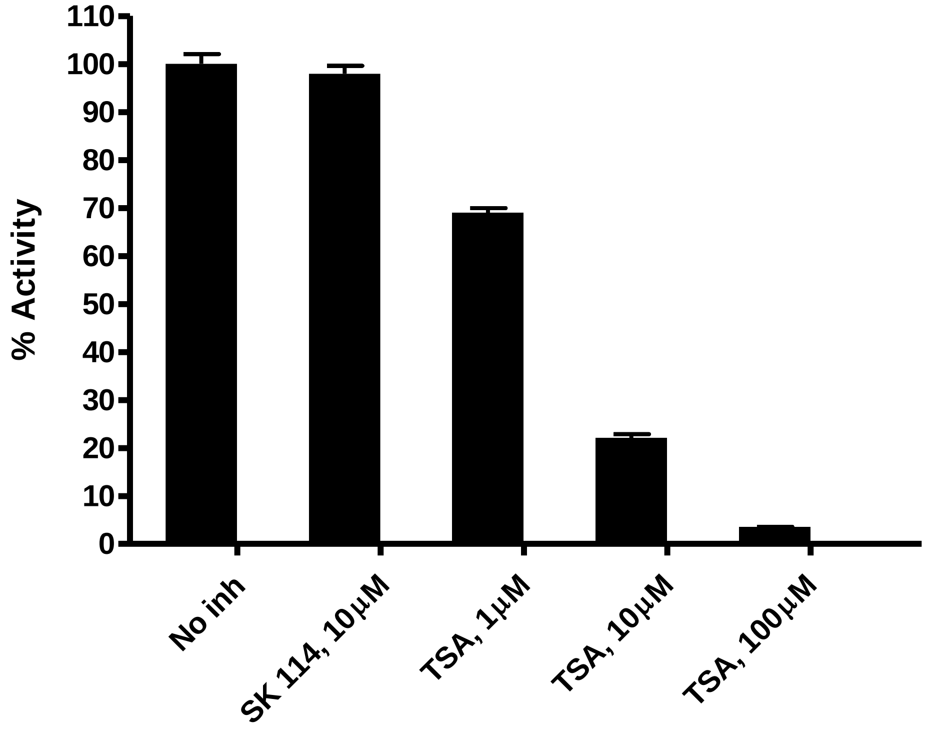

6042 Cornerstone Court West, Suite B
San Diego, CA 92121
Tel: 1.858.829.3082
Fax: 1.858.481.8694
Email: info@bpsbioscience.com

3.2.5. HDAC9

Table 3.2.5. Data for the Effect of the Compounds on HDAC9 Activity

Compounds	HDAC Activity (Fluorescence count)		% Activity		% Inhibition
	Repeat1	Repeat2	Repeat1	Repeat2	
No Compound	24306	25639	97	103	0
SK 114	23758	23540	95	94	5
TSA, 1μM	21090	21392	84	85	15
TSA, 10μM	10943	10103	42	39	60
TSA, 100μM	2006	1954	5	5	95
Background	788	781			

HDAC9 Activity

Substrate Conc.=2 μM Class 2a Substrate 1 (50040)

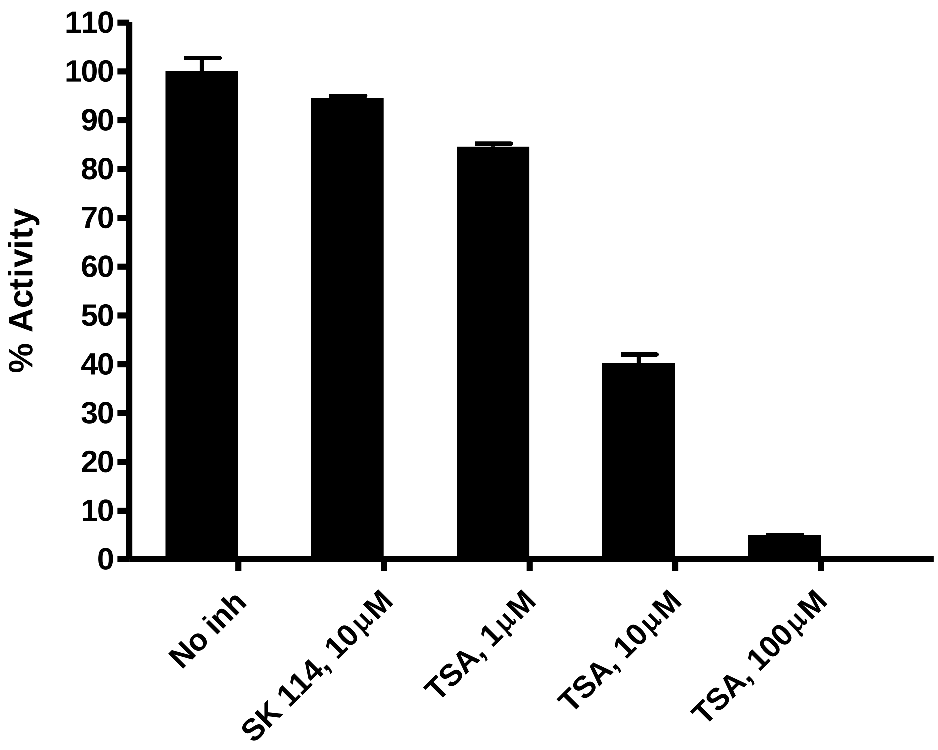

6042 Cornerstone Court West, Suite B
San Diego, CA 92121
Tel: 1.858.829.3082
Fax: 1.858.481.8694
Email: info@bpsbioscience.com

3.2.6. HDAC10

Table 3.2.6. Data for the Effect of the Compounds on HDAC10 Activity

Compounds	HDAC Activity (Fluorescence count)		% Activity		% Inhibition
	Repeat1	Repeat2	Repeat1	Repeat2	
No Compound	4848	4811	100	100	0
SK 114	3755	3696	73	71	28
SAHA, 0.01μM	3511	3492	67	66	34
SAHA, 0.1μM	1933	1851	27	25	74
SAHA, 1μM	991	963	3	2	97
Background	873	860			

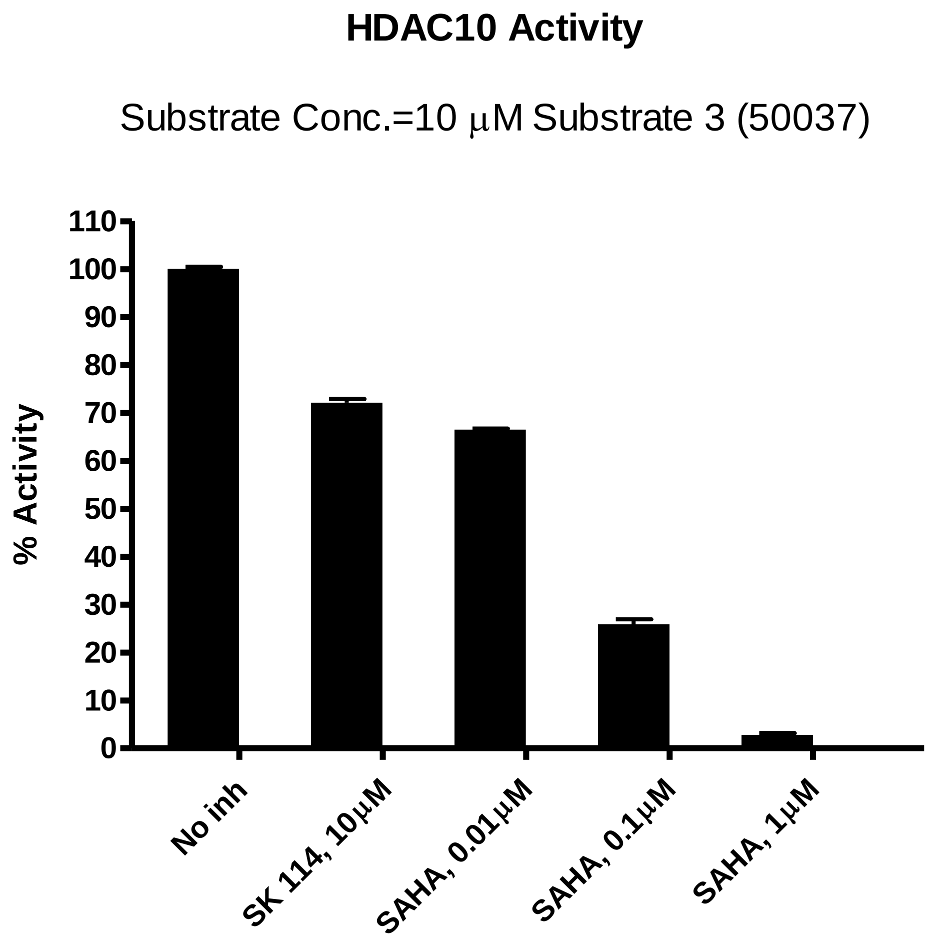

6042 Cornerstone Court West, Suite B
San Diego, CA 92121
Tel: 1.858.829.3082
Fax: 1.858.481.8694
Email: info@bpsbioscience.com

3.2.7. HDAC11

Table 3.2.7. Data for the Effect of the Compounds on HDAC11 Activity

Compounds	HDAC Activity (Fluorescence count)		% Activity		% Inhibition
	Repeat1	Repeat2	Repeat1	Repeat2	
No Compound	9260	9424	99	101	0
SK 114	8891	8842	95	94	6
TSA, 1μM	7825	8217	82	87	15
TSA, 10μM	5377	4979	54	49	49
TSA, 100μM	1492	1577	8	9	91
Background	763	775			

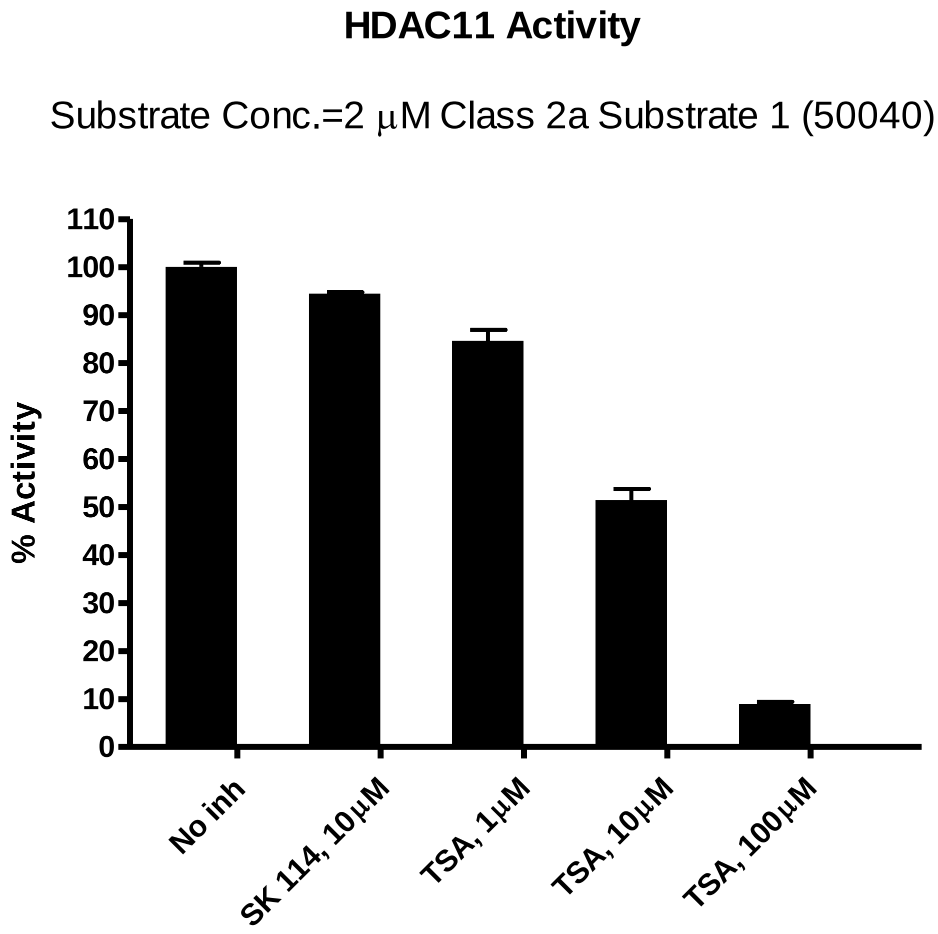

BPS Bioscience

6042 Cornerstone Court West, Suite B
San Diego, CA 92121
Tel: 1.858.829.3082
Fax: 1.858.481.8694
Email: info@bpsbioscience.com

4. Quality Assurance Statement

I certify that the results presented in this report were generated using the materials and methods mentioned and that these results reflect the Raw Data.

Henry Zhu, Ph.D.
President

10-16-18

Date

6042 Cornerstone Court West, Suite B
San Diego, CA 92121
Tel: 1.858.829.3082
Fax: 1.858.481.8694
Email: info@bpsbioscience.com

Assay Report

Histone Deacetylase (HDAC) Inhibitor Assays

Enzymatic Study of One Compound from Damanhour University

6042 Cornerstone Court West, Suite B
San Diego, CA 92121
Tel: 1.858.829.3082
Fax: 1.858.481.8694
Email: info@bpsbioscience.com

DHU_HDAC_180926

HDAC Inhibitor Assays

Study Sponsor:	Damanhour University
Attention:	Dr. Shaymaa Kassab
Address:	Damanhour University<br>425 Nargess Buildings<br>North Teseen St.<br>New Cairo City<br>Cairo, Egypt 11835
Study Director:	Henry Zhu, Ph.D.
Testing Facility:	BPS Bioscience Inc.<br>6042 Cornerstone Court West, Ste. B<br>San Diego, CA 92121<br>USA
Study Period:	
Report Version:	1
Report Date:	September 26, 2018

6042 Cornerstone Court West, Suite B
San Diego, CA 92121
Tel: 1.858.829.3082
Fax: 1.858.481.8694
Email: info@bpsbioscience.com

Study Director

Kevin Kurtz
Sr. Scientist II.

9-26-18

Date

Henry Zhu, Ph.D.
President

9-26-18

Date

6042 Cornerstone Court West, Suite B
San Diego, CA 92121
Tel: 1.858.829.3082
Fax: 1.858.481.8694
Email: info@bpsbioscience.com

CONTENTS

HISTONE DEACETYLASE (HDAC) INHIBITOR ASSAYS....1

ENZYMATIC STUDY OF ONE COMPOUND FROM FUNAKOSHI CO., LTD 1

HDAC INHIBITOR ASSAYS....2

STUDY DIRECTOR....3

1. PURPOSE OF THE STUDY 5

2. MATERIALS AND METHODS 6

2.1 MATERIALS 6
2.2 COMPOUNDS....6
2.3 EXPERIMENTAL CONDITIONS 6
2.3.1 Enzymes and Substrates 6
2.3.2 Assay Conditions....7
2.3.3 Data Analysis 7

3. ASSAY RESULTS 8

3.1. SUMMARY OF THE INHIBITORY EFFECTS OF THE COMPOUNDS ON HDAC ACTIVITIES 8
3.2. RESULTS OF THE EFFECTS OF THE COMPOUNDS ON INDIVIDUAL HDAC ACTIVITY 9
3.2.1. HDAC1....9
3.2.1.1. Compound 1 9
3.2.1.2. TSA 10
3.2.2. HDAC2....11
3.2.2.1. Compound 1 11
3.2.2.2. TSA 12

4. QUALITY ASSURANCE STATEMENT 11

6042 Cornerstone Court West, Suite B
San Diego, CA 92121
Tel: 1.858.829.3082
Fax: 1.858.481.8694
Email: info@bpsbioscience.com

1. Purpose of the Study

The purpose of the study is to determine the effects of one compound from Damanhour University on the activities of recombinant HDAC6 using an *in vitro* enzymatic assay.

6042 Cornerstone Court West, Suite B
San Diego, CA 92121
Tel: 1.858.829.3082
Fax: 1.858.481.8694
Email: info@bpsbioscience.com

2. Materials and Methods

2.1 Materials

TSA is purchased from Selleck (Houston, TX, Catalog number S1045)
HDAC Assay Buffer (BPS catalog number 50031)
HDAC Assay Developer (BPS catalog number 50030)
HDAC Substrate 3 (BPS number 50037)

2.2 Compounds

The test compounds are supplied by Damanhour University.

Compound I.D.	Compound Supplied	Stock Concentration	Dissolving Solvent	Test Range (µM)	Intermediate Dilution
SK 114	Powder	10mM	DMSO	0.0003 - 10	10 % DMSO in HDAC Assay Buffer
TSA*	Powder	10mM	DMSO	0.00003 - 1	10 % DMSO in HDAC Assay Buffer

*Reference Compound

2.3 Experimental Conditions

2.3.1 Enzymes and Substrates

Assay	Catalog #	Enzyme Lot #	Enzyme Used (ng) / Reaction	Substrate
HDAC6	50006	180709-G	10	10µM HDAC Substrate 3

BPS Bioscience
6042 Cornerstone Court West, Suite B
San Diego, CA 92121
Tel: 1.858.829.3082
Fax: 1.858.481.8694
Email: info@bpsbioscience.com

2.3.2 Assay Conditions

All of the compounds are dissolved in DMSO. The serial dilution of the compounds was first performed in 100% DMSO with the highest concentration at 1mM. Each intermediate compound dilution (in 100% DMSO) will then get directly diluted 10x fold into assay buffer for an intermediate dilution of 10% DMSO in HDAC assay buffer and 5µl of the dilution was added to a 50µl reaction so that the final concentration of DMSO is 1% in all of reactions.

The enzymatic reactions for the HDAC enzymes were conducted in duplicate at 37ºC for 30 minutes in a 50µl mixture containing HDAC assay buffer, 5µg BSA, an HDAC substrate (see 2.3.1), a HDAC enzyme (see 2.3.1) and a test compound (see 2.2).

After enzymatic reactions, 50µl of 2 x HDAC Developer was added to each well for the HDAC enzymes and the plate was incubated at room temperature for an additional 15 minutes.

Fluorescence intensity was measured at an excitation of 360 nm and an emission of 460 nm using a Tecan Infinite M1000 microplate reader.

2.3.3 Data Analysis

HDAC activity assays were performed in duplicates at each concentration. The fluorescent intensity data were analyzed using the computer software, Graphpad Prism. In the absence of the compound, the fluorescent intensity (F_t) in each data set was defined as 100% activity. In the absence of HDAC, the fluorescent intensity (F_b) in each data set was defined as 0% activity. The percent activity in the presence of each compound was calculated according to the following equation: %activity = $(F-F_b)/(F_t-F_b)$, where F= the fluorescent intensity in the presence of the compound.

The values of % activity versus a series of compound concentrations were then plotted using non-linear regression analysis of Sigmoidal dose-response curve generated with the equation $Y=B+(T-B)/1+10^{((LogEC50-X)\times Hill\ Slope)}$, where Y=percent activity, B=minimum percent activity, T=maximum percent activity, X= logarithm of compound and Hill Slope=slope factor or Hill coefficient. The IC_{50} value was determined by the concentration causing a half-maximal percent activity.

6042 Cornerstone Court West, Suite B
San Diego, CA 92121
Tel: 1.858.829.3082
Fax: 1.858.481.8694
Email: info@bpsbioscience.com

3. Assay Results

3.1. Summary of the Inhibitory Effects of the Compounds on HDAC Activities

The IC_{50} of the compound against HDACSs are summarized on Table 3.1.

Table 3.1 Inhibitory Effects of the Compound on HDAC Activities (IC_{50})

Enzymes	IC_{50} (μM)	
	SK 114	Reference
HDAC6	0.51	0.0050 TSA

6042 Cornerstone Court West, Suite B
San Diego, CA 92121
Tel: 1.858.829.3082
Fax: 1.858.481.8694
Email: info@bpsbioscience.com

3.2. Results of the Effects of the Compounds on Individual HDAC Activity

3.2.1. HDAC6

3.2.1.1. SK 114

Table 3.2.1.1. Data for the Effect of SK 114 on HDAC6 Activity

SK 114 [μM]	HDAC6 Activity (Fluorescence count)		% Activity	
	Repeat1	Repeat2	Repeat1	Repeat2
No Compound	10606	11145	97	103
0.0003	10826	10776	100	99
0.001	10630	10878	98	100
0.003	10925	10394	100	95
0.01	10366	10832	95	100
0.03	10207	10266	93	94
0.1	9486	9009	86	82
0.3	6885	7093	61	63
1	3997	4067	33	33
3	2002	2139	13	14
10	1163	1127	5	5
Background	650	666		

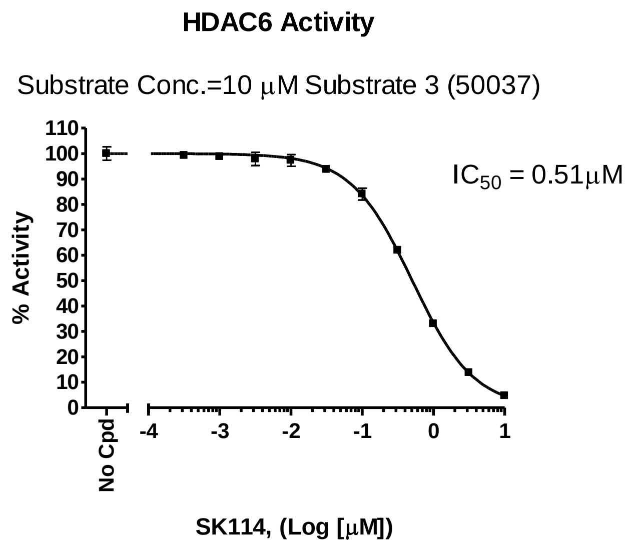

6042 Cornerstone Court West, Suite B
San Diego, CA 92121
Tel: 1.858.829.3082
Fax: 1.858.481.8694
Email: info@bpsbioscience.com

3.2.1.2. TSA

Table 3.2.1.2. Data for the Effect of TSA on HDAC6 Activity

TSA [μM]	HDAC6 Activity (Fluorescence count)		% Activity	
	Repeat1	Repeat2	Repeat1	Repeat2
No Compound	10769	10770	100	100
0.00003	10819	10617	100	98
0.0001	10525	10372	98	96
0.0003	10358	9667	96	89
0.001	8662	9172	79	84
0.003	7078	6509	63	58
0.01	4304	4110	36	34
0.03	2204	2026	15	13
0.1	1142	1123	5	5
0.3	868	891	2	2
1	687	674	0	0
Background	686	640		

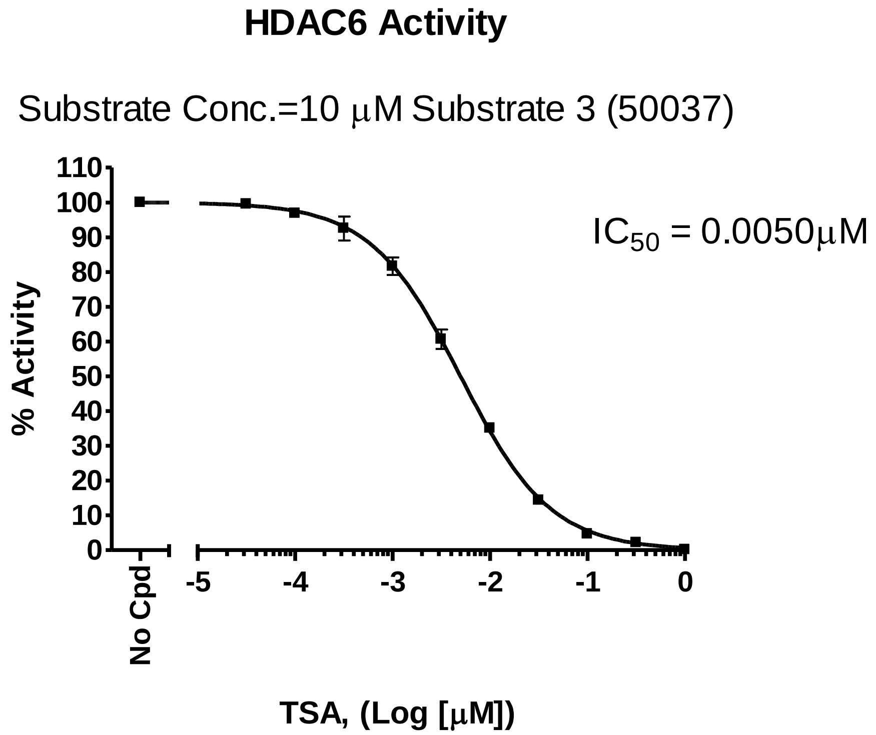

6042 Cornerstone Court West, Suite B
San Diego, CA 92121
Tel: 1.858.829.3082
Fax: 1.858.481.8694
Email: info@bpsbioscience.com

4. Quality Assurance Statement

I certify that the results presented in this report were generated using the materials and methods mentioned and that these results reflect the Raw Data.

Henry Zhu, Ph.D.
President

9-26-18

Date

6042 Cornerstone Court West, Suite B
San Diego, CA 92121
Tel: 1.858.829.3082
Fax: 1.858.481.8694
Email: info@bpsbioscience.com

Assay Report

Histone Deacetylases (HDAC6) Inhibitor Assays

Enzymatic Study of Three Compounds from Damanhour University

6042 Cornerstone Court West, Suite B
San Diego, CA 92121
Tel: 1.858.829.3082
Fax: 1.858.481.8694
Email: info@bpsbioscience.com

Damanhour Univ._HDAC6_20180608

HDACs Inhibitor Assays

Study Sponsor:	Damanhour University
Attention:	Dr. Shaymaa Kassab
Address:	425 Nargess Buildings, North Teseen St., New Cairo City, 11835Street, Cairo, Egypt
Study Director:	Henry Zhu, Ph.D.
Testing Facility:	BPS Bioscience Inc.<br>6042 Cornerstone Court West, Ste. B<br>San Diego, CA 92121<br>USA
Study Period:	
Report Version:	1
Report Date:	June 08th, 2018

6042 Cornerstone Court West, Suite B
San Diego, CA 92121
Tel: 1.858.829.3082
Fax: 1.858.481.8694
Email: info@bpsbioscience.com

Study Director

Cinzia Ambrosi
Scientist II

06/08/2018
Date

Henry Zhu, Ph.D.
President

06/08/2018
Date

6042 Cornerstone Court West, Suite B
San Diego, CA 92121
Tel: 1.858.829.3082
Fax: 1.858.481.8694
Email: info@bpsbioscience.com

CONTENTS

1. PURPOSE OF THE STUDY **5**

2. MATERIALS AND METHODS **6**

2.1. MATERIALS 6
2.2. COMPOUNDS 6
2.3. EXPERIMENTAL CONDITIONS 6
2.3.1. Enzymes and Substrates ***6***
2.3.2. Assay Conditions ***6***
2.3.3. Data Analysis ***7***

3. ASSAY RESULTS **8**

3.1. SUMMARY OF THE INHIBITORY EFFECTS OF THREE COMPOUNDS ON HDAC6 ACTIVITY 8
3.2. RESULTS OF THE EFFECTS OF THE COMPOUNDS ON HDAC6 ACTIVITY 9
3.2.1. HDAC ***9***
3.2.1.1. HDAC6 .... ***9***

4. QUALITY ASSURANCE STATEMENT **10**

6042 Cornerstone Court West, Suite B
San Diego, CA 92121
Tel: 1.858.829.3082
Fax: 1.858.481.8694
Email: info@bpsbioscience.com

1. Purpose of the Study

The purpose of the study is to determine the effects of three compounds from Damanhour University on the enzymatic activities of recombinant human HDAC6 using an *in vitro* enzymatic assay.

6042 Cornerstone Court West, Suite B
San Diego, CA 92121
Tel: 1.858.829.3082
Fax: 1.858.481.8694
Email: info@bpsbioscience.com

2. Materials and Methods

2.1. Materials

HDAC Assay Buffer (BPS catalog number 50031).
HDAC Assay Developer (BPS catalog number 50030).
HDAC Substrate 3 (BPS catalog number 50037).

2.2. Compounds

The test compounds are supplied by Damanhour University.

SK 111	Powder	10 mM	DMSO	10	10 % DMSO in HDAC Assay Buffer
SK 112	Powder	10 mM	DMSO	10	10 % DMSO in HDAC Assay Buffer
SK 113	Powder	10 mM	DMSO	10	10 % DMSO in HDAC Assay Buffer

*Reference compound.

2.3. Experimental Conditions

2.3.1. Enzymes and Substrates

Assay	Catalog #	Enzyme Lot #	Enzyme Used (ng) / Reaction	Substrate
HDAC6	50006	170520	10	10 µM HDAC Substrate 3 50037

2.3.2. Assay Conditions

The compounds were dissolved in DMSO. A series of dilutions of the compounds was prepared with 10% DMSO in HDAC assay buffer and 5µl of the dilution was added to a 50µl reaction so that the final concentration of DMSO is 1% in all of the reactions.

The enzymatic reactions for the HDAC enzymes were conducted in duplicate at 37ºC for 30 minutes in a 50µl mixture containing HDAC assay buffer, 5µg BSA, an HDAC substrate (see 2.3.1), a HDAC enzyme (see 2.3.1) and a test compound (see 2.2).

6042 Cornerstone Court West, Suite B
San Diego, CA 92121
Tel: 1.858.829.3082
Fax: 1.858.481.8694
Email: info@bpsbioscience.com

After enzymatic reactions, 50µl of 2 x HDAC Developer was added to each well and the plate was incubated at room temperature for an additional 15 minutes.

Fluorescence intensity was measured at an excitation of 360 nm and an emission of 460 nm using a Tecan Infinite M1000 microplate reader.

2.3.3. Data Analysis

HDAC activity assays were performed in duplicate. The fluorescent intensity data were analyzed using the computer software, GraphPad Prism. In the absence of the compound, the fluorescent intensity (F_t) in each data set was defined as 100% activity. In the absence of HDAC, the fluorescent intensity (F_b) in each data set was defined as 0% activity. The percent activity in the presence of each compound was calculated according to the following equation: %activity = $(F-F_b)/(F_t-F_b)$, where F= the fluorescent intensity in the presence of the compound.

The values of percentage activity were plotted on a bar graph.

6042 Cornerstone Court West, Suite B
San Diego, CA 92121
Tel: 1.858.829.3082
Fax: 1.858.481.8694
Email: info@bpsbioscience.com

3. Assay Results

3.1. Summary of the Inhibitory Effects of Three Compounds on HDAC6 Activity

The percentage inhibition of the compounds against HDAC6 is summarized in Table 3.1.

Table 3.1. Inhibitory Effects of the Compounds on HDAC6 Activity

Compounds	Conc. (μM)	% Inhibition
		HDAC6
SK 111	10	12
SK 112	10	14
SK 113	10	35

BPS Bioscience
6042 Cornerstone Court West, Suite B
San Diego, CA 92121
Tel: 1.858.829.3082
Fax: 1.858.481.8694
Email: info@bpsbioscience.com

3.2. Results of the Effects of the Compounds on HDAC6 Activity

3.2.1. HDAC

3.2.1.1. HDAC6

Table 3.2.1.1. Data for the Effect of Three Compounds on HDAC6 Activity.

Compounds	Conc. µM	HDAC6 Activity (Fluorescence count)		% Activity		% Inhibition
		Repeat1	Repeat2	Repeat1	Repeat2	
No Compound		2178	2119	102	98	0
SK 111	10	1973	1840	91	84	12
SK 112	10	1929	1823	89	83	14
SK 113	10	1477	1456	65	64	35
Background		217	200			

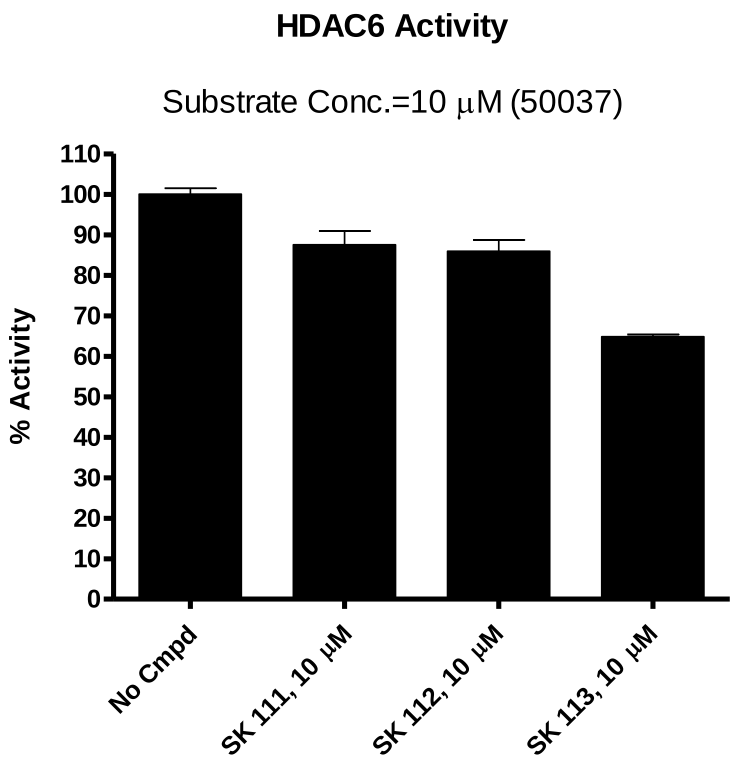

6042 Cornerstone Court West, Suite B
San Diego, CA 92121
Tel: 1.858.829.3082
Fax: 1.858.481.8694
Email: info@bpsbioscience.com

4. Quality Assurance Statement

I certify that the results presented in this report were generated using the materials and methods mentioned and that these results reflect the Raw Data.

Henry Zhu, Ph.D.
President

06/08/2018
Date

6042 Cornerstone Court West, Suite B
San Diego, CA 92121
Tel: 1.858.829.3082
Fax: 1.858.481.8694
Email: info@bpsbioscience.com

Assay Report

Histone Deacetylases (HDAC1 and HDAC8) Inhibitor Assays

Enzymatic Study of Eight Compounds from Damanhour University

6042 Cornerstone Court West, Suite B
San Diego, CA 92121
Tel: 1.858.829.3082
Fax: 1.858.481.8694
Email: info@bpsbioscience.com

Damanhour Univ._ HDAC1 and HDAC8_20180605

HDACs Inhibitor Assays

Study Sponsor:	Damanhour University
Attention:	Dr. Shaymaa Kassab
Address:	425 Nargess Buildings, North Teseen St., New Cairo City, 11835Street, Cairo, Egypt
Study Director:	Henry Zhu, Ph.D.
Testing Facility:	BPS Bioscience Inc.<br>6042 Cornerstone Court West, Ste. B<br>San Diego, CA 92121<br>USA
Study Period:	
Report Version:	1
Report Date:	June 05th, 2018

6042 Cornerstone Court West, Suite B
San Diego, CA 92121
Tel: 1.858.829.3082
Fax: 1.858.481.8694
Email: info@bpsbioscience.com

Study Director

Cinzia Ambrosi
Scientist II

06/05/2018
Date

Henry Zhu, Ph.D.
President

06/05/2018
Date

6042 Cornerstone Court West, Suite B
San Diego, CA 92121
Tel: 1.858.829.3082
Fax: 1.858.481.8694
Email: info@bpsbioscience.com

CONTENTS

1. PURPOSE OF THE STUDY 5

2. MATERIALS AND METHODS 6

2.1. MATERIALS 6
2.2. COMPOUNDS 6
2.3. EXPERIMENTAL CONDITIONS 7
2.3.1. Enzymes and Substrates 7
2.3.2. Assay Conditions....7
2.3.3. Data Analysis 8

3. ASSAY RESULTS 9

3.1. SUMMARY OF THE INHIBITORY EFFECTS OF EIGHT COMPOUNDS ON INDIVIDUAL HDAC ACTIVITIES 9
3.2. RESULTS OF THE EFFECTS OF THE COMPOUNDS ON INDIVIDUAL HDAC ACTIVITY 10
3.2.1. HDAC....10
3.2.1.1. HDAC1....10
3.2.1.2. HDAC8....12

4. QUALITY ASSURANCE STATEMENT 14

6042 Cornerstone Court West, Suite B
San Diego, CA 92121
Tel: 1.858.829.3082
Fax: 1.858.481.8694
Email: info@bpsbioscience.com

1. Purpose of the Study

The purpose of the study is to determine the effects of eight compounds from Damanhour University on the enzymatic activities of recombinant human HDAC1 and HDAC8 using an *in vitro* enzymatic assay.

6042 Cornerstone Court West, Suite B
San Diego, CA 92121
Tel: 1.858.829.3082
Fax: 1.858.481.8694
Email: info@bpsbioscience.com

2. Materials and Methods

2.1. Materials

SAHA is purchased from Cayman Chemicals (Ann Arbor, MI, Catalog Number 10009929).
TSA is purchased from Selleck (Houston, TX, Catalog number S1045).
HDAC Assay Buffer (BPS catalog number 50031).
HDAC Assay Developer (BPS catalog number 50030).
HDAC Substrate 3 (BPS catalog number 50037).
HDAC Class 2a Substrate 1 (BPS catalog number 50040).

2.2. Compounds

The test compounds are supplied by Damanhour University.

Compound Name	Compound Supplied	Stock Concentration	Dissolving Solvent	Test Range (µM)	Intermediate Dilution
SK 411	Powder	10 mM	DMSO	10	10 % DMSO in HDAC Assay Buffer
SK 421	Powder	10 mM	DMSO	10	10 % DMSO in HDAC Assay Buffer
SK 431	Powder	10 mM	DMSO	10	10 % DMSO in HDAC Assay Buffer
SK 441	Powder	10 mM	DMSO	10	10 % DMSO in HDAC Assay Buffer
SK 111	Powder	10 mM	DMSO	10	10 % DMSO in HDAC Assay Buffer
SK 112	Powder	10 mM	DMSO	10	10 % DMSO in HDAC Assay Buffer

6042 Cornerstone Court West, Suite B
San Diego, CA 92121
Tel: 1.858.829.3082
Fax: 1.858.481.8694
Email: info@bpsbioscience.com

SK 113	Powder	10 mM	DMSO	10	10 % DMSO in HDAC Assay Buffer
SK 114	Powder	10 mM	DMSO	10	10 % DMSO in HDAC Assay Buffer
*SAHA	Powder	10 mM	DMSO	0.03-0.3-3	10 % DMSO in HDAC Assay Buffer
*TSA	Powder	10 mM	DMSO	1-10-100	10 % DMSO in HDAC Assay Buffer

*Reference compound.

2.3. Experimental Conditions

2.3.1. Enzymes and Substrates

Assay	Catalog #	Enzyme Lot #	Enzyme Used (ng) / Reaction	Substrate
HDAC1	50051	170501-1	7.2	10 µM HDAC Substrate 3 50037
HDAC8	50008	131120-2	15	2 µM HDAC Class2a Substrate 1 50040

2.3.2. Assay Conditions

The compounds were dissolved in DMSO. A series of dilutions of the compounds was prepared with 10% DMSO in HDAC assay buffer and 5µl of the dilution was added to a 50µl reaction so that the final concentration of DMSO is 1% in all of the reactions.

The enzymatic reactions for the HDAC enzymes were conducted in duplicate at 37°C for 30 minutes in a 50µl mixture containing HDAC assay buffer, 5µg BSA, an HDAC substrate (see 2.3.1), a HDAC enzyme (see 2.3.1) and a test compound (see 2.2).

After enzymatic reactions, 50µl of 2 x HDAC Developer was added to each well and the plate was incubated at room temperature for an additional 15 minutes.

6042 Cornerstone Court West, Suite B
San Diego, CA 92121
Tel: 1.858.829.3082
Fax: 1.858.481.8694
Email: info@bpsbioscience.com

Fluorescence intensity was measured at an excitation of 360 nm and an emission of 460 nm using a Tecan Infinite M1000 microplate reader.

2.3.3. Data Analysis

HDAC activity assays were performed in duplicate. The fluorescent intensity data were analyzed using the computer software, GraphPad Prism. In the absence of the compound, the fluorescent intensity (F_t) in each data set was defined as 100% activity. In the absence of HDAC, the fluorescent intensity (F_b) in each data set was defined as 0% activity. The percent activity in the presence of each compound was calculated according to the following equation: %activity = $(F-F_b)/(F_t-F_b)$, where F= the fluorescent intensity in the presence of the compound.

The values of percentage activity were plotted on a bar graph.

6042 Cornerstone Court West, Suite B
San Diego, CA 92121
Tel: 1.858.829.3082
Fax: 1.858.481.8694
Email: info@bpsbioscience.com

3. Assay Results

3.1. Summary of the Inhibitory Effects of Eight Compounds on Individual HDAC Activities

The percentage inhibition of the compounds against HDAC enzymes is summarized in Table 3.1.

Table 3.1. Inhibitory Effects of the Compounds on HDAC Activities

Compounds	Conc. (μM)	% Inhibition	
		HDAC1	HDAC8
SK 411	10	18	12
SK 421	10	13	14
SK 431	10	14	1
SK 441	10	6	14
SK 111	10	9	1
SK 112	10	9	12
SK 113	10	11	10
SK 114	10	36	56
SAHA	0.03	47	-
	0.3	87	-
	3	98	-
TSA	1	-	53
	10	-	89
	100	-	97

6042 Cornerstone Court West, Suite B
San Diego, CA 92121
Tel: 1.858.829.3082
Fax: 1.858.481.8694
Email: info@bpsbioscience.com

3.2. Results of the Effects of the Compounds on Individual HDAC Activity

3.2.1. HDAC

3.2.1.1. HDAC1

Table 3.2.1.1. Data for the Effect of Eight Compounds on HDAC1 Activity

Compounds	Conc. µM	HDAC1 Activity (Fluorescence count)		% Activity		% Inhibition
		Repeat1	Repeat2	Repeat1	Repeat2	
No Compound		7275	7226	100	100	0
SK 411	10	6198	5938	84	80	18
SK 421	10	6286	6424	86	88	13
SK 431	10	6172	6511	84	89	14
SK 441	10	6773	6894	93	95	6
SK 111	10	6681	6661	92	91	9
SK 112	10	6926	6399	95	87	9
SK 113	10	6402	6635	87	91	11
SK 114	10	4867	4839	64	64	36
SAHA	0.03	4074	4147	53	54	47
	0.3	1446	1436	14	13	87
	3	674	682	2	2	98
Background		540	533			

6042 Cornerstone Court West, Suite B
San Diego, CA 92121
Tel: 1.858.829.3082
Fax: 1.858.481.8694
Email: info@bpsbioscience.com

HDAC1 Activity

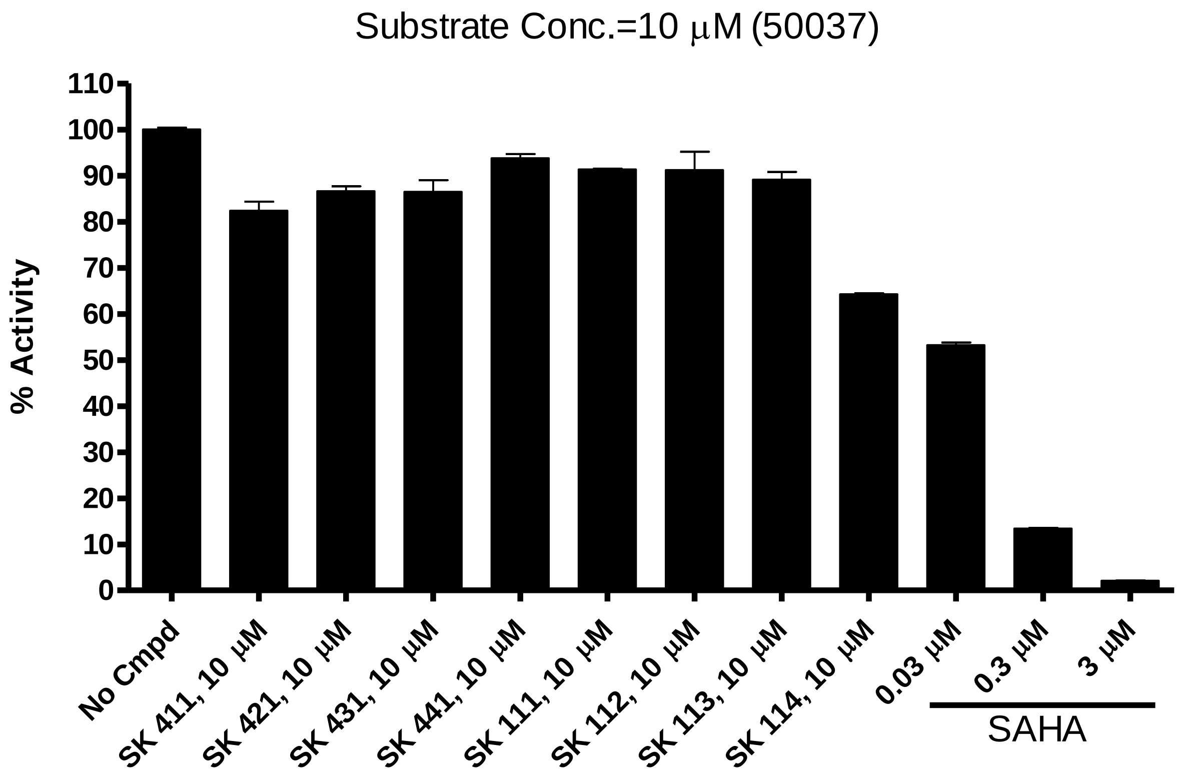

6042 Cornerstone Court West, Suite B
San Diego, CA 92121
Tel: 1.858.829.3082
Fax: 1.858.481.8694
Email: info@bpsbioscience.com

3.2.1.2. HDAC8

Table 3.2.1.2. Data for the Effect of Eight Compounds on HDAC8 Activity

Compounds	Conc. µM	HDAC8 Activity (Fluorescence count)		% Activity		% Inhibition
		Repeat1	Repeat2	Repeat1	Repeat2	
No Compound		23713	23174	101	99	0
SK 411	10	20823	20876	88	89	12
SK 421	10	20935	19774	89	84	14
SK 431	10	22919	23620	98	101	1
SK 441	10	20229	20206	86	86	14
SK 111	10	23107	23215	99	99	1
SK 112	10	20279	21308	86	91	12
SK 113	10	21462	20810	91	88	10
SK 114	10	10300	11252	42	46	56
TSA	1	11432	11605	47	47	53
	10	3472	3451	11	11	89
	100	1489	1491	3	3	97
Background		916	926			

6042 Cornerstone Court West, Suite B
San Diego, CA 92121
Tel: 1.858.829.3082
Fax: 1.858.481.8694
Email: info@bpsbioscience.com

HDAC8 Activity

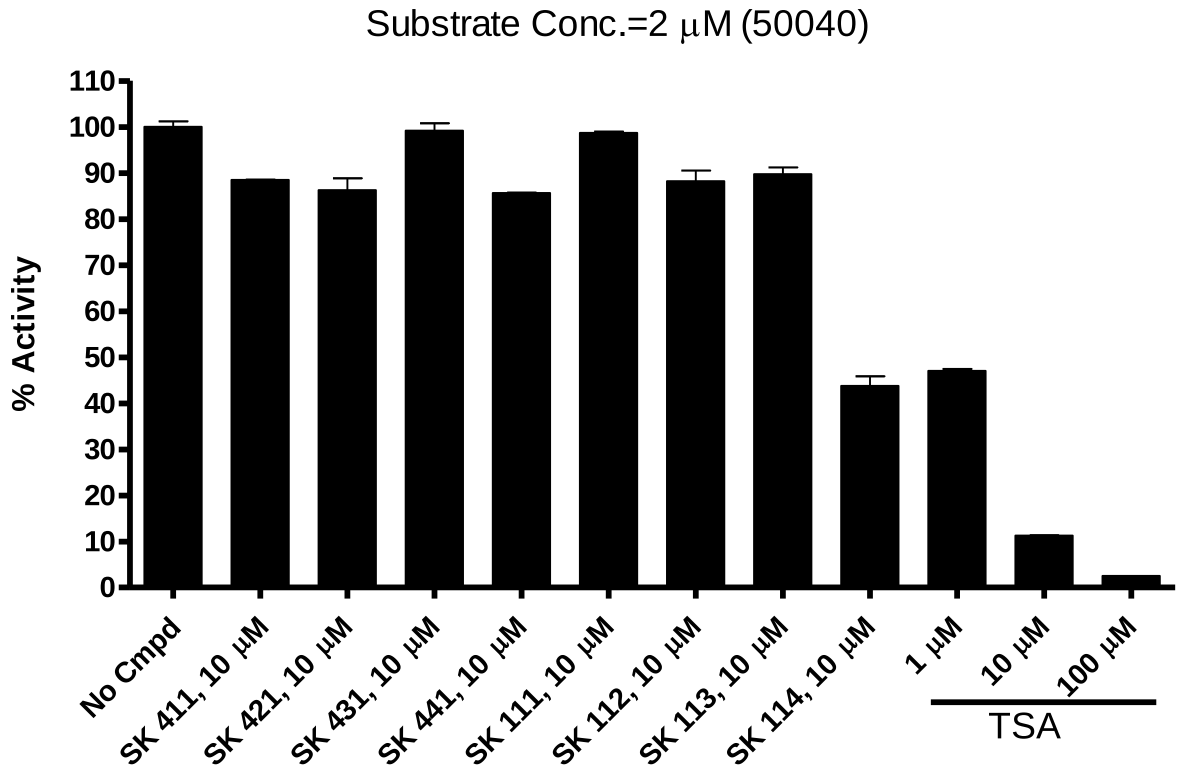

6042 Cornerstone Court West, Suite B
San Diego, CA 92121
Tel: 1.858.829.3082
Fax: 1.858.481.8694
Email: info@bpsbioscience.com

4. Quality Assurance Statement

I certify that the results presented in this report were generated using the materials and methods mentioned and that these results reflect the Raw Data.

Henry Zhu, Ph.D.
President

06/05/2018
Date

6042 Cornerstone Court West, Suite B
San Diego, CA 92121
Tel: 1.858.829.3082
Fax: 1.858.481.8694
Email: info@bpsbioscience.com

Assay Report

Histone Deacetylase (HDAC) Inhibitor Assays

Enzymatic Study of Seven Compounds from Damanhour University

6042 Cornerstone Court West, Suite B
San Diego, CA 92121
Tel: 1.858.829.3082
Fax: 1.858.481.8694
Email: info@bpsbioscience.com

DHU_HDAC_180828

HDAC Inhibitor Assays

Study Sponsor:	Damanhour University
Attention:	Dr. Shaymaa Kassab
Address:	Damanhour University<br>425 Nargess Buildings<br>North Teseen St.<br>New Cairo City<br>Cairo, Egypt 11835
Study Director:	Henry Zhu, Ph.D.
Testing Facility:	BPS Bioscience Inc.<br>6042 Cornerstone Court West, Ste. B<br>San Diego, CA 92121<br>USA
Study Period:	
Report Version:	1
Report Date:	August 28, 2018

BPS Bioscience

6042 Cornerstone Court West, Suite B
San Diego, CA 92121
Tel: 1.858.829.3082
Fax: 1.858.481.8694
Email: info@bpsbioscience.com

Study Director

Kevin Kurtz
Sr. Scientist II.

8-28-18

Date

Henry Zhu, Ph.D.
President

8-28-18

Date

6042 Cornerstone Court West, Suite B
San Diego, CA 92121
Tel: 1.858.829.3082
Fax: 1.858.481.8694
Email: info@bpsbioscience.com

CONTENTS

HISTONE DEACETYLASE (HDAC) INHIBITOR ASSAYS....1

ENZYMATIC STUDY OF SEVEN COMPOUNDS FROM DAMANHOUR UNIVERSITY....1

HDAC INHIBITOR ASSAYS....2

STUDY DIRECTOR....3

1. PURPOSE OF THE STUDY....5

2. MATERIALS AND METHODS....6

2.1 MATERIALS....6
2.2 COMPOUNDS....6
2.3 EXPERIMENTAL CONDITIONS....7
2.3.1 Enzymes and Substrates....7
2.3.2 Assay Conditions....7
2.3.3 Data Analysis....7

3. ASSAY RESULTS....8

3.1. SUMMARY OF THE INHIBITORY EFFECTS OF THE COMPOUND ON INDIVIDUAL HDAC ACTIVITIES....8
3.2. RESULTS OF THE EFFECTS OF THE COMPOUNDS ON INDIVIDUAL HDAC ACTIVITY....9
3.2.1. HDAC2....9
3.2.2. HDAC6....10

4. QUALITY ASSURANCE STATEMENT....11

6042 Cornerstone Court West, Suite B
San Diego, CA 92121
Tel: 1.858.829.3082
Fax: 1.858.481.8694
Email: info@bpsbioscience.com

1. Purpose of the Study

The purpose of the study is to determine the effects of seven compound from Damanhour University on the enzymatic activities of recombinant human HDAC2 and HDAC6 using an in vitro enzymatic assay.

6042 Cornerstone Court West, Suite B
San Diego, CA 92121
Tel: 1.858.829.3082
Fax: 1.858.481.8694
Email: info@bpsbioscience.com

2. Materials and Methods

2.1 Materials

HDAC Assay Buffer (BPS catalog number 50031)
HDAC Assay Developer (BPS catalog number 50030)
HDAC Substrate 3 (BPS number 50037)
SAHA is purchased from Cayman Chemicals (Ann Arbor, MI, Catalog Number 10009929)

2.2 Compounds

The test compounds are supplied by Damanhour University.

Compound I.D.	Compound Supplied	Stock Concentration	Dissolving Solvent	Test Range (μM)	Intermediate Dilution
SK 111	Powder	10mM	DMSO	10	10 % DMSO in HDAC Assay Buffer
SK 112	Powder	10mM	DMSO	10	10 % DMSO in HDAC Assay Buffer
SK 113	Powder	10mM	DMSO	10	10 % DMSO in HDAC Assay Buffer
SK 411	Powder	10mM	DMSO	10	10 % DMSO in HDAC Assay Buffer
SK 421	Powder	10mM	DMSO	10	10 % DMSO in HDAC Assay Buffer
SK 431	Powder	10mM	DMSO	10	10 % DMSO in HDAC Assay Buffer
SK 441	Powder	10mM	DMSO	10	10 % DMSO in HDAC Assay Buffer
SAHA*	Powder	10mM	DMSO	1, 0.1, 0.01	10 % DMSO in HDAC Assay Buffer

*Reference Compound

6042 Cornerstone Court West, Suite B
San Diego, CA 92121
Tel: 1.858.829.3082
Fax: 1.858.481.8694
Email: info@bpsbioscience.com

2.3 Experimental Conditions

2.3.1 Enzymes and Substrates

Assay	Catalog #	Enzyme Lot #	Enzyme Used (ng) / Reaction	Substrate
HDAC2	50002	160701	7.5	10µM HDAC Substrate 3
HDAC6	50006	180709-G	10	10µM HDAC Substrate 3

2.3.2 Assay Conditions

The compounds are dissolved in DMSO. A series of dilutions of the compounds were prepared with 10% DMSO in HDAC assay buffer and 5µl of the dilution was added to a 50µl reaction so that the final concentration of DMSO is 1% in all of reactions.

The enzymatic reactions for the HDAC enzymes were conducted in duplicate at 37ºC for 30 minutes in a 50µl mixture containing HDAC assay buffer, 5µg BSA, an HDAC substrate (see 2.3.1), a HDAC enzyme (see 2.3.1) and a test compound (see 2.2).

After enzymatic reactions, 50µl of 2 x HDAC Developer was added to each well for the HDAC enzymes and the plate was incubated at room temperature for an additional 15 minutes.

Fluorescence intensity was measured at an excitation of 360 nm and an emission of 460 nm using a Tecan Infinite M1000 microplate reader.

2.3.3 Data Analysis

HDAC activity assays were performed in duplicates at each concentration. The fluorescent intensity data were analyzed using the computer software, Graphpad Prism. In the absence of the compound, the fluorescent intensity (F_t) in each data set was defined as 100% activity. In the absence of HDAC, the fluorescent intensity (F_b) in each data set was defined as 0% activity. The percent activity in the presence of each compound was calculated according to the following equation: %activity = $(F-F_b)/(F_t-F_b)$, where F= the fluorescent intensity in the presence of the compound.

The values of percentage activity were plotted on a bar graph.

6042 Cornerstone Court West, Suite B
San Diego, CA 92121
Tel: 1.858.829.3082
Fax: 1.858.481.8694
Email: info@bpsbioscience.com

3. Assay Results

3.1. Summary of the Inhibitory Effects of the Compound on Individual HDAC Activities

The percentage inhibition of the seven compound against HDACs is summarized on Table 3.1.

Table 3.1 Inhibitory Effects of the Compounds on HDAC Activities

Inhibitors	% Inhibition	
	HDAC2	HDAC6
SK 111	9	-
SK 112	8	-
SK 113	8	-
SK 411	6	6
SK 421	7	2
SK 431	3	2
SK 441	5	2
SAHA, 0.01μM	14	46
SAHA, 0.1μM	52	82
SAHA, 1μM	85	97

6042 Cornerstone Court West, Suite B
San Diego, CA 92121
Tel: 1.858.829.3082
Fax: 1.858.481.8694
Email: info@bpsbioscience.com

3.2. Results of the Effects of the Compounds on Individual HDAC Activity

3.2.1. HDAC2

Table 3.2.1. Data for the Effect of the Compounds on HDAC2 Activity

Compounds	HDAC Activity (Fluorescence count)		% Activity		% Inhibition
	Repeat1	Repeat2	Repeat1	Repeat2	
No Compound	10956	10865	100	100	0
SK 111	9847	10070	90	92	9
SK 112	9939	10233	91	94	8
SK 113	10222	9850	93	90	8
SK 411	10112	10480	92	96	6
SK 421	10118	10276	92	94	7
SK 431	10651	10547	98	97	3
SK 441	10652	10114	98	92	5
SAHA, 0.01µM	9558	9311	87	85	14
SAHA, 0.1µM	5465	5504	48	48	52
SAHA, 1µM	1935	2070	14	15	85
Background	464	467			

HDAC2 Activity

Substrate Conc.=10 µM Substrate 3 (50037)

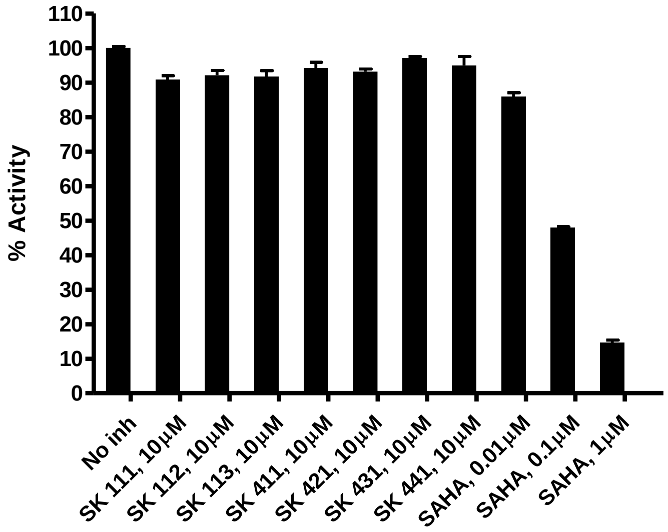

3.2.2. <u>HDAC6</u>

Table 3.2.2. Data for the Effect of the Compounds on HDAC6 Activity

Compounds	HDAC Activity (Fluorescence count)		% Activity		% Inhibition
	Repeat1	Repeat2	Repeat1	Repeat2	
No Compound	10327	10262	100	100	0
SK 411	9311	10067	90	98	6
SK 421	9965	10323	97	100	2
SK 431	10135	10117	98	98	2
SK 441	9870	10295	96	100	2
SAHA, 0.01µM	5983	5613	56	52	46
SAHA, 0.1µM	2027	2416	16	20	82
SAHA, 1µM	752	811	3	3	97
Background	476	483			

HDAC6 Activity

Substrate Conc.=10 µM Substrate 3 (50037)

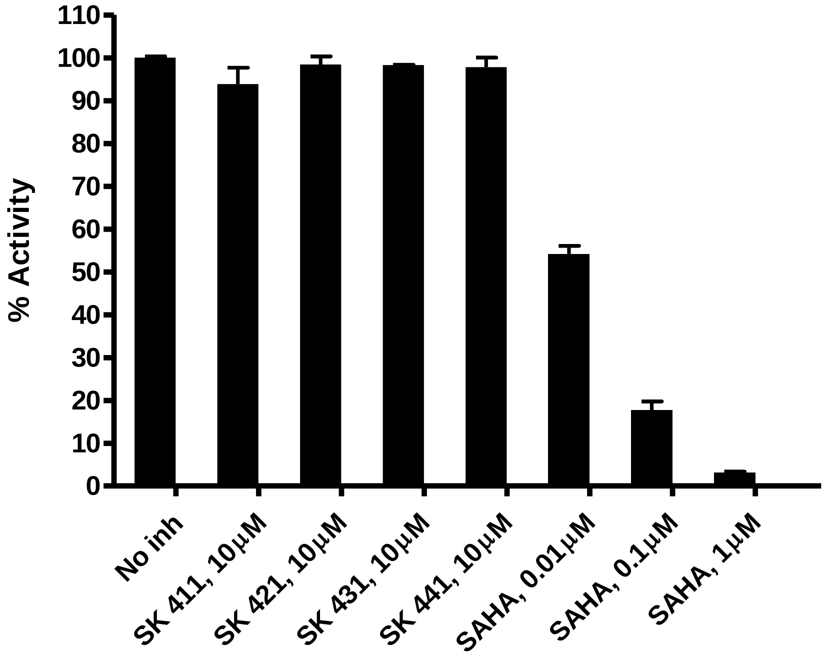

6042 Cornerstone Court West, Suite B
San Diego, CA 92121
Tel: 1.858.829.3082
Fax: 1.858.481.8694
Email: info@bpsbioscience.com

4. Quality Assurance Statement

I certify that the results presented in this report were generated using the materials and methods mentioned and that these results reflect the Raw Data.

Henry Zhu, Ph.D.
President

8-28-18

Date

SK114 cytotoxicity Assay

Aya Alserw

Experimental design

- Based on previous pilot experiment, the highest SK114 conc. (100µM) effectively and solely lowered cell index and induced cell death in 72hrs, thus this experiment was performed with higher concentration range. A serial dilution from 400µM to 3.125µM was added on CCHE-45 cells
- Cells were incubated with the drug for 96hrs
- Experiment was done in technical duplicates

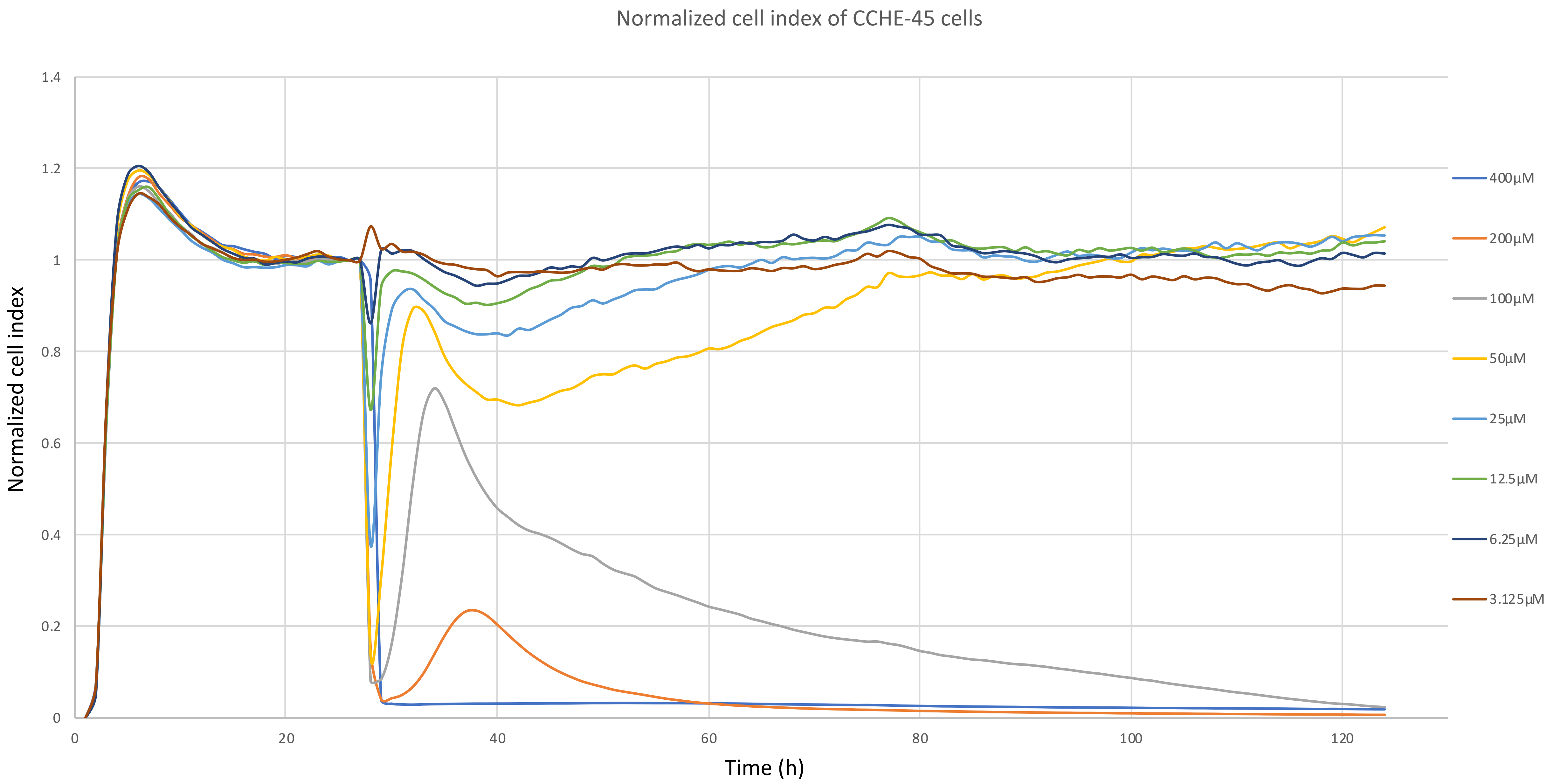
Normalized cell index of CCHE-45 cells
Normalized cell index
Time (h)
1.4
1.2
1
0.8
0.6
0.4
0.2
0
0
20
40
60
80
100
120
400µM
200µM
100µM
50µM
25µM
12.5µM
6.25µM
3.125µM

SK114 IC50

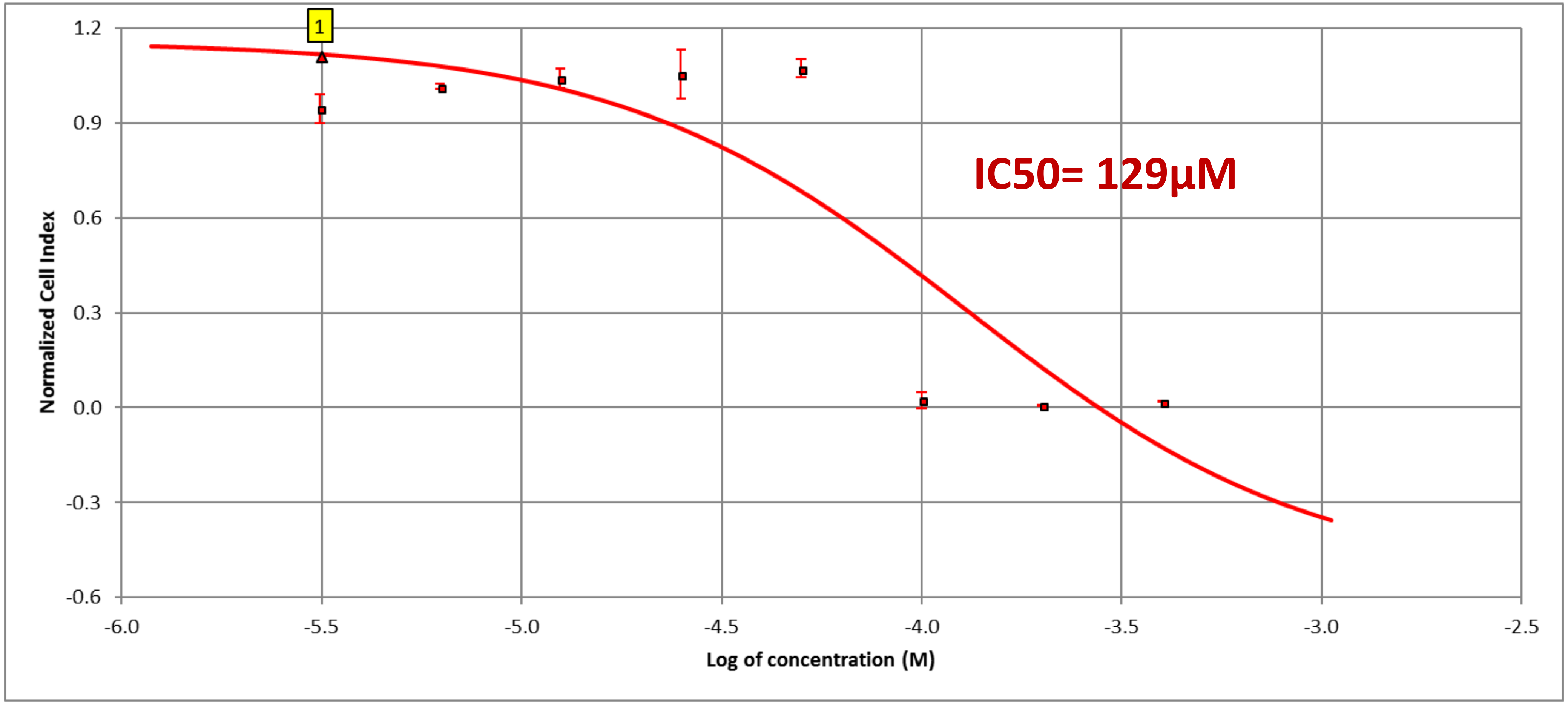
1
IC50= 129µM
Normalized Cell Index
1.2
0.9
0.6
0.3
0.0
-0.3
-0.6
-6.0
-5.5
-5.0
-4.5
-4.0
-3.5
-3.0
-2.5
Log of concentration (M)

Future experiments

- Experiment will be conducted in triplicates
- Western blot and Immunofluorescence will follow

Last trial for CPC

21/12/2018

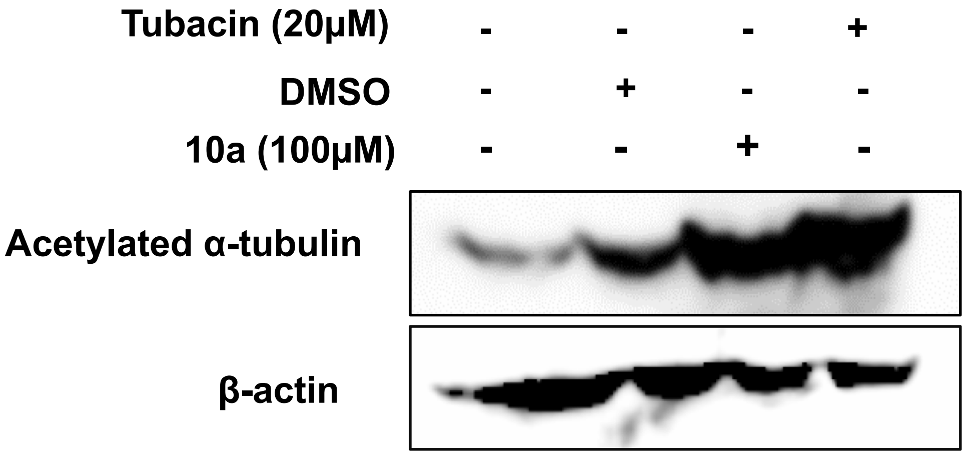
Tubacin (20μM) - - - +
DMSO - + - -
10a (100μM) - - + -
Acetylated α-tubulin
β-actin

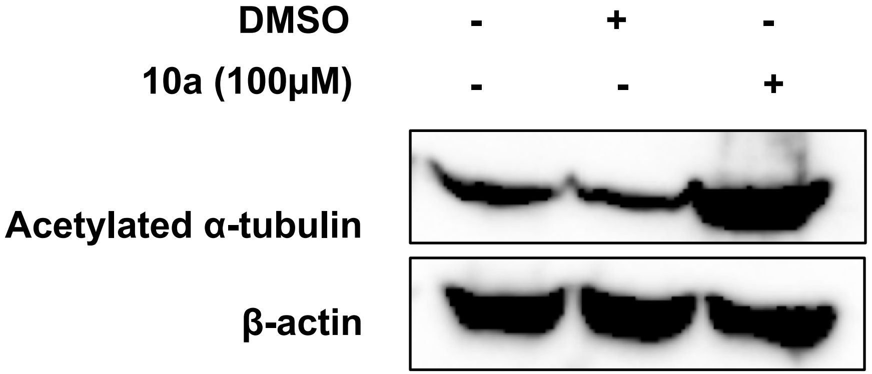
DMSO - + -
10a (100μM) - - +
Acetylated α-tubulin
β-actin
